# DNA Vaccine Delivered by a Needle-Free Injection Device Improves Potency of Priming for Antibody and CD8+ T-Cell Responses after rAd5 Boost in a Randomized Clinical Trial

**DOI:** 10.1371/journal.pone.0059340

**Published:** 2013-04-08

**Authors:** Barney S. Graham, Mary E. Enama, Martha C. Nason, Ingelise J. Gordon, Sheila A. Peel, Julie E. Ledgerwood, Sarah A. Plummer, John R. Mascola, Robert T. Bailer, Mario Roederer, Richard A. Koup, Gary J. Nabel, the VRC 008 Study Team

**Affiliations:** 1 Vaccine Research Center, National Institute of Allergy and Infectious Diseases, National Institutes of Health, Bethesda, Maryland, United States of America; 2 Biostatistics Research Branch, Division of Clinical Research, National Institute of Allergy and Infectious Diseases, National Institutes of Health, Bethesda, Maryland, United States of America; 3 Walter Reed Army Institute of Research, U.S. Military HIV Research Program, Silver Spring, Maryland, United States of America; Istituto Superiore di Sanità, Italy

## Abstract

**Background:**

DNA vaccine immunogenicity has been limited by inefficient delivery. Needle-free delivery of DNA using a CO_2_-powered Biojector® device was compared to delivery by needle and syringe and evaluated for safety and immunogenicity.

**Methods:**

Forty adults, 18–50 years, were randomly assigned to intramuscular (IM) vaccinations with DNA vaccine, VRC-HIVDNA016-00-VP, (weeks 0, 4, 8) by Biojector® 2000™ or needle and syringe (N/S) and boosted IM at week 24 with VRC-HIVADV014-00-VP (rAd5) with N/S at 10^10^ or 10^11^ particle units (PU). Equal numbers per assigned schedule had low (≤500) or high (>500) reciprocal titers of preexisting Ad5 neutralizing antibody.

**Results:**

120 DNA and 39 rAd5 injections were given; 36 subjects completed follow-up research sample collections. IFN-γ ELISpot response rates were 17/19 (89%) for Biojector® and 13/17 (76%) for N/S delivery at Week 28 (4 weeks post rAd5 boost). The magnitude of ELISpot response was about 3-fold higher in Biojector® compared to N/S groups. Similar effects on response rates and magnitude were observed for CD8+, but not CD4+ T-cell responses by ICS. Env-specific antibody responses were about 10-fold higher in Biojector-primed subjects.

**Conclusions:**

DNA vaccination by Biojector® was well-tolerated and compared to needle injection, primed for greater IFN-γ ELISpot, CD8+ T-cell, and antibody responses after rAd5 boosting.

**Trial Registration:**

ClinicalTrials.gov NCT00109629

## Introduction

Immunization with plasmid DNA is a promising technology for gene-based antigen delivery. It has many advantages over microbial vectors, in part because of its simplicity. In particular, there is no pre-existing vector immunity, construction and manufacturing is rapid, and candidate DNA vaccines have been extremely stable and safe [1]. However, DNA vaccine immunogenicity in humans has been less than expected from preclinical studies in mice and monkeys. The basis for this is not fully known, but it is likely that inefficient transfection, particularly through the plasma and nuclear membranes of host cells, is a major factor.

Over the last 10 years the Vaccine Research Center has made a significant effort to evaluate the DNA technology platform for vaccines against several virus diseases including HIV, West Nile virus (WNV), SARS coronavirus, filoviruses, and influenza viruses [2]–[9]. A number of steps have been taken to optimize protein expression including codon modification, altered promoters, translation enhancer motifs, and other changes to the plasmid backbone [10]. A variety of doses have been explored, and early in the program a decision was made to use the needle-free Biojector® device based on published reports of Biojector® delivery improving the antibody response to DNA vaccines in animals [11] and humans [12], [13] compared to delivery by needle and syringe (N/S). In particular, vaccine studies for WNV [6], [8], influenza [9], and HIV [14]–[17] have demonstrated favorable properties of DNA immunization that merit further development. Three doses of a WNV DNA vaccine expressing the prM and E proteins induced substantial neutralizing antibody responses comparable to those seen in horses known to be protected [6], [8]. In the influenza program, a single dose of H5 influenza HA DNA vaccine primed a four-fold increase in HAI antibody titers in >80% of subjects following a single 6 month boost with unadjuvanted inactivated H5N1 vaccine compared to 2 doses of inactivated H5N1 vaccine [9]. This concept is now being evaluated in Phase II studies using seasonal influenza vaccines. In the HIV vaccine development program, DNA primed broad and durable T cell responses and consistent antibody responses following boosting with rAd5 [15]–[18]. This regimen is now being evaluated in the HVTN 505 Phase IIb test-of-concept study to determine efficacy. Given the progression of DNA vaccines into advanced clinical trials it is important to understand how delivery approaches may contribute to their immunogenicity.

We report here the results of a Phase I study comparing Biojector® to N/S delivery of a DNA vaccine in a healthy volunteer population. A factorial design was used to evaluate the effect of pre-existing Ad5 immunity and dose of the rAd5 boost in addition to Biojector® delivery of the DNA. We found that Biojector significantly improved humoral and cellular immunogenicity and that pre-existing Ad5 immunity and booster dose did not significantly affect vaccine-induced immune responses.

## Methods

The protocol for this trial and supporting CONSORT checklist are available as supporting information; see Protocol S1 and Checklist S1.

### Ethics Statement

These studies were approved by the National Institute of Allergy and Infectious Diseases Institutional Review Board, and were performed in accordance with 45 CFR Part 46, U.S. Food and Drug Administration regulations, and principles expressed in the Declaration of Helsinki. All subjects signed written informed consent documents.

### Objectives

To characterize the safety, tolerability, and immunogenicity profile of a DNA prime, rAd5 boost vaccine regimen comparing two different methods of intramuscular DNA administration - needle and syringe vs. a needle-free pressure injection device (Biojector®).

### Participants

Healthy, HIV-negative subjects between the ages of 18 and 50 at the time of enrollment.

### Study Design

VRC 008 was conducted at the National Institutes of Health (NIH) Clinical Center, Bethesda, MD by the Vaccine Research Center (VRC), National Institute of Allergy and Infectious Diseases (NIAID), NIH, Department of Health and Human Services (DHHS). Forty subjects, twenty with low (≤1∶500) and twenty with high (>1∶500) adenovirus serotype 5 antibody (Ad5Ab) titers at screening, were randomized in a 1∶1 ratio to receive the DNA vaccinations intramuscularly (IM) by either needle and syringe or by Biojector® 2000® and in a 1∶1 ratio to receive the booster vaccination at a dose of 10^10^ or 10^11^ particle units (PU). Randomization sequence was obtained by the statistician using computer-generated random numbers and was stratified by Ad5Ab titer as positive or negative to achieve balance across study groups. Within each stratum of Ad5Ab titer, five subjects were assigned to each combination of factors (DNA administration device and rAd5 booster dose) completely at random. The DNA administration device became known to both clinicians and subjects after completion of an electronic enrollment in the study database, while the dose of rAd5 vaccine remained blinded to all except the statistician and pharmacist until after the safety data collection following the rAd5 vaccine boost was completed.

All rAd5 vaccinations were administered IM by needle and syringe. DNA vaccine was given at Weeks 0, 4 and 8, followed by one injection of rAd5 vaccine at Week 24. Subjects self-reported for solicited reactogenicity parameters on 5-day diary cards following each injection. Local reactogenicity of the DNA injections was further documented by clinician assessments and photographs 3 days after injection. Laboratory and clinical follow-up continued through Week 42. The Division of AIDS 2004 table was used for grading severity of unsolicited adverse events (AEs), which were also coded using the Medical Dictionary for Regulatory Activities (MedDRA) for preparing summary data. There were no significant changes to the study design after trial commencement except for the addition of a long-term contact at Week 94, as part of the response to comments made by the Food and Drug Administration (FDA) on the protocol design. The study was fully accrued and completed as designed. The primary objective related to safety of the vaccination regimens and secondary objectives related to immune responses at 4 weeks after the 3^rd^ DNA vaccine and 6 weeks after the rAd5 vaccine boost were the basis for the sample size. Other secondary objectives were to determine Ad5 neutralizing antibody titers at 4 weeks after rAd5 boost and social impact of participating in an HIV-1 vaccine clinical trial.

### Vaccine

The study vaccines, developed by the VRC, NIAID, NIH, were VRC-HIVDNA016-00-VP, composed of 6 closed, circular DNA plasmids that encode for HIV-1 Gag, Pol and Nef (from clade B) and Env glycoprotein from clade A, clade B, and clade C combined in equal proportions (16.67% each by weight) in phosphate buffered saline (PBS) [4] and VRC-HIVADV014-00-VP, composed of 4 recombinant non-replicating adenoviral serotype 5 vectors that encode for HIV-1 Gag/Pol polyproteins (from clade B) and Env glycoprotein from clade A, clade B, and clade C, combined in a 3∶1:1∶1 ratio, respectively, in a final formulation buffer (FFB) [19], [20].

### Peptides

Peptides (15-mers overlapping by 11) matching the sequences of the HIV-specific antigens expressed by the vaccines were used at >70% purity. They were pooled according to antigen (EnvA, EnvB, EnvC, Gag, Pol, Nef)., and were used at a final concentration of 2.5 µg/ml to stimulated vaccine-induced T cells *in vitro*.

### Enzyme-linked Immunospot Assays (ELISpot)

The frequency of antigen/vaccine-specific cells was determined as previously described [3]. Cryopreserved PBMCs were stimulated overnight by peptide pools representing the individual vaccine antigens. IFN-γ ELISpot was performed using a commercial kit (BD Biosciences), read on a CTL ELISpot image analyzer (Cellular Technology Ltd; Cleveland, OH), and expressed as mean spot-forming cells (SFC) per million PBMC.

### Flow Cytometric Analysis and Intracellular Cytokine Staining (ICS)

Cryopreserved PBMCs were stimulated by peptide pools for 6 hours with brefeldin A. Permeabilized fixed cells were evaluated by flow cytometry for expression of CD3, CD8, CD4, and IFN-γ and/or IL-2, then analyzed using FlowJo software (TreeStar; Ashland, OR) as previously described [3].

### Measurement of Antibody Responses

Standardized research ELISAs were performed to delineate the antibody response to viral antigens encoded within the vaccine. End-point titers of antibodies were determined using 96-well Immulon2 (Dynex Technologies) plates coated with a preparation of purified recombinant HIV proteins derived from the same sequences as the vaccine antigens [3]. End-point titer was calculated as the most dilute serum concentration that gave an optical density reading of >0.2 above background. Subjects were screened via a commercial EIA (Abbott Laboratories HIV-1/HIV-2 rDNA) and Western blot (Mayo Laboratory, Genetic Systems Western blot kit by BioRad Laboratories, Inc).

Serum neutralizing antibody levels were measured using single round replication-defective Env-pseudoviruses and an engineered cell line that expresses luciferase upon viral infection. The methods and virus strains were previously described [21], [22].

### HIV-1 Diagnostic Testing

The Abbott HIVAB ™ HIV-1/HIV-2 rDNA EIA kit was used for diagnostic testing. For reactive results, Western blot analyses were done at Mayo Laboratory using the GS HIV-1 Western Blot (BioRad Laboratories, Redmond, WA). The AMPLICOR HIV-1 MONITOR Test ver.1.5 (Roche Molecular Systems, Indianapolis, IN), was used for HIV RNA PCR testing regardless of EIA result at all testing time points. An exploratory analysis to assess vaccine-induced sero-positivity/sero-reactivity (VISP/R) [23] was performed with alternative peptide-based diagnostic assays including the SELECTEST [24] and multiple versions of HIV diagnostic tests produced by BioRad according to the manufacturer instructions and published methods.

### Data Analysis and Statistics

The statistical methods followed the same conventions that were used for the predecessor studies [19], [25] and were done post hoc. Measures of positive T-cell response are defined by both a statistical test and a minimum magnitude threshold. Specifically, for ELISpot, a positive response is defined as at least 59 SFC per million PBMC and a non-background corrected mean that is at least 4 fold greater than the mean negative stimulation for the sample. For ICS, a positive response was defined as one with both a p-value of <0.01 from a Fisher’s Exact Test and a background-subtracted magnitude that exceeded a pre-specified threshold. The pre-specified threshold was based on validation of negative samples and was allowed to vary for different peptides. For CD4+ cells, a value of.045 was used for all peptides; for CD8+ cells, the threshold was.07 for Env C,.058 for Gag B, and.045 for all other peptides. For ELISA, a positive response is defined as any measure with end-point titer ≥30. All comparisons of proportions between arms are done using Fisher’s Exact Test; paired comparisons within individuals were done using Wilcoxon Signed Rank tests (post-DNA response compared to rAd5 vector boosting).

## Results

### Study Conduct and Population

**Figure 1:**
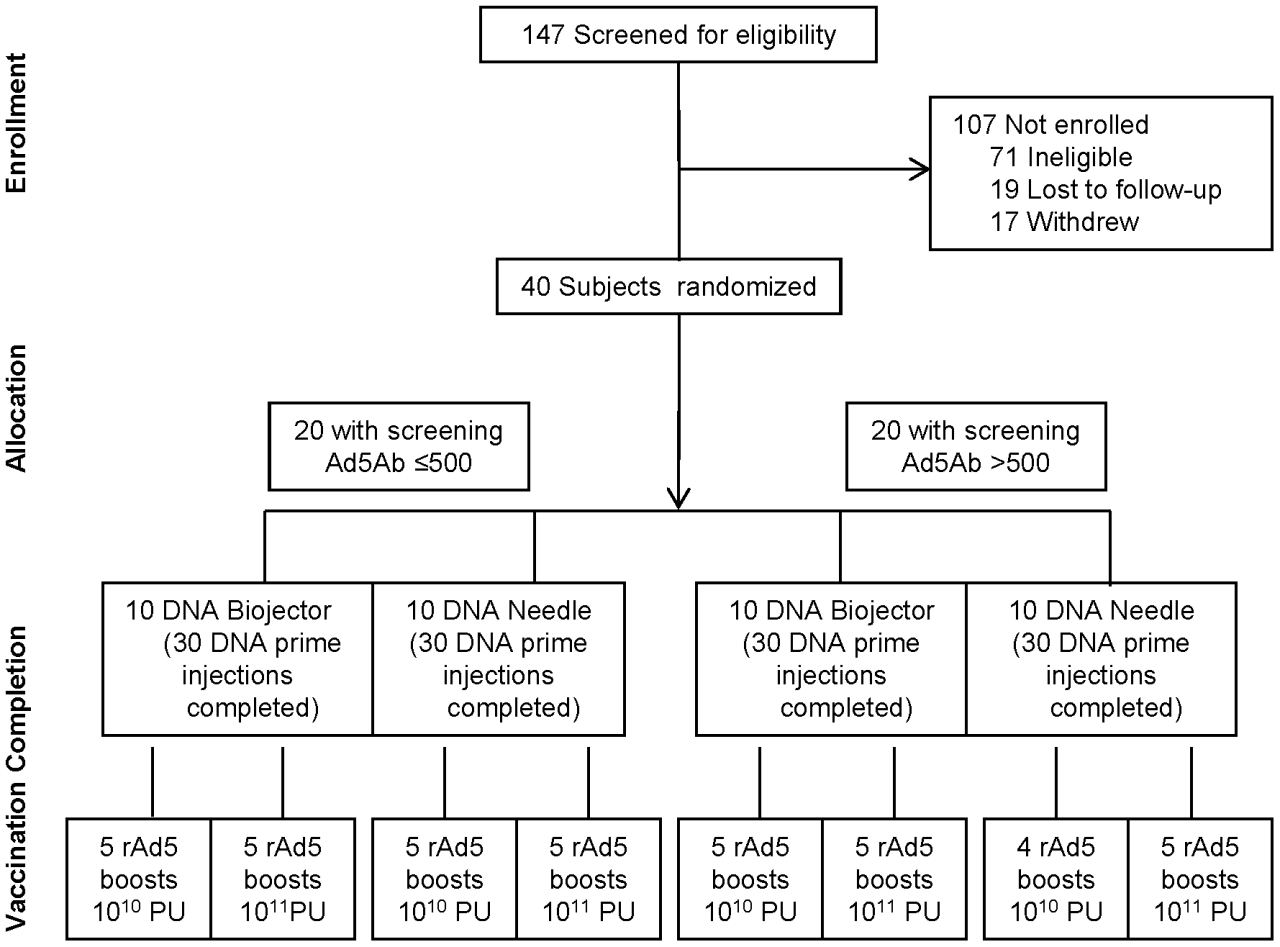
Schematic diagram of study design and completion of vaccination schedule. The CONSORT diagram indicates the number of subjects screened to complete enrollment of 40 subjects in a factorial study design. Subjects were stratified by pre-existing Ad5 neutralizing antibody reciprocal titer of ≤500 or >500 then randomized to receive DNA priming by Biojector® or needle and syringe (N/S). In addition, 50% of each subgroup was boosted with rAd5 at 10^10^ PU and the other 50% received 10^11^ PU.

Forty subjects were enrolled between May and September 2005, final study injection was administered in February 2006, and the last long-term contact was August in 2007. Participant demographics are shown in Table 1. A balance of subjects with “low” and “high” pre-existing Ad5 antibody in each vaccination schedule was achieved. The 20 subjects in the “low (≤1∶500) Ad5 Ab” group included 16 with undetectable (<12) Ad5 neutralizing titer at screening, and 4 with relatively low titers (48, 87, 106 and 107). The 20 in the “high Ad5 Ab” group included 18 with titers >1000 and 2 with titers of 583 and 691. Enrollment, randomization, and follow-up is shown in Figure 1; 39 of 40 participants completed all 4 study vaccinations and 36 completed the protocol through the planned clinical follow-up for 42 weeks. One subject in the group with high Ad5Ab titer at screening, who was randomized to needle administration of DNA vaccine and rAd5 10^10^ PU, chose to not receive the rAd5 booster vaccination.

**Table 1:** Baseline Characteristics of Participants.

Category	Characteristic	Low Ad5Ab Titer at Screening (N = 20)	High Ad5Ab Titer at Screening (N = 20)	Overall (N = 40)
GENDER – no. (%)	Male	14 (70)	12 (60)	26 (65)
	Female	6 (30)	8 (40)	14 (35)
RACE – no. (%)	Asian	0	2 (10)	2 (5)
	Black or African American	2 (10)	2 (10)	4 (10)
	White	18 (90)	16 (80)	34 (85)
ETHNICITY – no. (%)	Non-Hispanic/Latino	20 (100)	19 (95)	39 (97.5)
	Hispanic/Latino	0	1 (5)	1 (2.5)
AGE – mean [std. dev.]		30.5 [8.8]	28.1 [7.4]	29.3 [8.1]
BMI – mean [std. dev.]		25.2 [3.5]	26.3 [3.9]	25.8 [3.7]

### Vaccine Safety

There were no adverse experiences requiring an IND safety report. With close, prospective scrutiny of the vaccination sites, a small skin lesion, described as a papule or scab, was commonly observed by the study team during clinical evaluation after DNA injection by Biojector® [38/60 (63.3%) Biojector® DNA injections], but less frequently noted [11/60 (18%); p<.001] on the self-reported diary card completed by study participants; all resolved without treatment. When present, these lesions were primarily observed on days 2–4 following vaccination and were not observed by clinicians or subjects after the 60 N/S DNA vaccine injections. After rAd5 injections, there were 4 reactogenicity events that met study criteria for prompt review by the safety monitoring team. These were grade 3 fever (maximum temperature 39.9°C) within the first day after injection, and 3 episodes of erythema or induration with maximum diameter >9 cm for which the onset 3 to 5 days post injection and duration averaged 4 days. The overall reactogenicity of the DNA vaccine by Biojector® as compared to N/S injection and of rAd5 vaccinations at 10^10^ PU dose as compared to 10^11^ PU dose is shown in Tables 2 and 3. The reactogenicity events resolved without sequelae and were consistent with earlier Phase I experience with the study vaccines [4], [19]. After the rAd5 vaccine, 2/19 (10.5%) at the 10^10^ PU dosage as compared to 11/20 (55%; p = .006) at the 10^11^ PU dosage had a pattern of moderate to severe systemic reactogenicity; which except for one case had acute onset in the first 24 hours after injection and frequently was accompanied by fever. There was no evident difference in the incidence of moderate to severe reactogenicity by pre-existing Ad5 antibody titer [7/20 (35%) in low titer group compared to 6/19 (32%) in high titer group].

**Table 2:** Worst Severity of Local Reactogenicity Following DNA and rAd5 Vaccinations.

Local Symptoms Intensity	Biojector® DNA (N = 20)	Needle DNA (N = 20)	All DNA (N = 40)	rAd5 10^10^ (N = 19)	rAd5 10^11^ (N = 20)	All rAd5 (N = 39)
	number (%) of Subjects					
Pain/Tenderness						
None	0	5 (25)	5 (12.5)	3 (15.8)	1 (5)	4 (10.3)
Mild	20 (100)	15 (75)	35 (87.5)	15 (78.9)	16 (80)	31 (79.5)
Moderate	0	0	0	1 (5.3)	3 (15)	4 (10.3)
Severe	0	0	0	0	0	0
Swelling						
None	4 (20)	20 (100)	24 (60)	16 (84.2)	17 (85)	33 (84.6)
Mild	16 (80)	0	16 (40)	3 (15.8)	1 (5)	4 (10.3)
Moderate	0	0	0	0	2 (10)	2 (5.1)
Severe	0	0	0	0	0	0
Redness						
None	6 (30)	18 (90)	24 (60)	14 (73.7)	17 (85)	31 (79.5)
Mild	14 (70)	2 (10)	16 (40)	4 (21.1)	2 (10)	6 (15.4)
Moderate	0	0	0	1 (5.3)	1 (5)	2 (5.1)
Severe	0	0	0	0	0	0
Any Local Symptom						
None	0	5 (25)	5 (12.5)	3 (15.8)	1 (5)	4 (10.3)
Mild	20 (100)	15 (75)	35 (87.5)	14 (73.8)	14 (70)	28 (71.8)
Moderate	0	0	0	2 (10.5)	5 (25)	7 (17.9)
Severe	0	0	0	0	0	0

**Table 3:** Worst Severity of Systemic Reactogenicity Following DNA and rAd5 Vaccinations.

Systemic Symptoms Intensity	Biojector® DNA (N = 20)	Needle DNA (N = 20)	All DNA (N = 40)	rAd5 10^10^ (N = 19)	rAd5 10^11^ (N = 20)	All rAd5 (N = 39)
	number (%) of Subjects					
Malaise						
None	13 (65)	9 (45)	22 (55)	9 (47.4)	3 (15)	12 (30.8)
Mild	7 (35)	8 (40)	15 (37.5)	9 (47.4)	10 (50)	19 (48.7)
Moderate	0	3 (15)	3 (7.5)	1 (5.3)	7 (35)	8 (20.5)
Severe	0	0	0	0	0	0
Myalgia						
None	16 (80)	15 (75)	31 (77.5)	6 (31.6)	3 (15)	9 (23.1)
Mild	4 (20)	5 (25)	9 (22.5)	11 (57.9)	9 (45)	20 (51.3)
Moderate	0	0	0	2 (10.5)	8 (40)	10 (25.6)
Severe	0	0	0	0	0	0
Headache						
None	16 (80)	10 (50)	26 (65)	11 (57.9)	4 (20)	15 (38.5)
Mild	4 (20)	7 (35)	11 (27.5)	7 (36.8)	10 (50)	17 (43.6)
Moderate	0	3 (15)	3 (7.5)	1 (5.3)	6 (30)	7 (17.9)
Severe	0	0	0	0	0	0
Chills						
None	19 (95)	19 (95)	38 (95)	12 (63.2)	9 (45)	21 (53.8)
Mild	1 (5)	1 (5)	2 (5)	6 (31.6)	6 (30)	12 (30.8)
Moderate	0	0	0	1 (5.3)	5 (25)	6 (15.4)
Severe	0	0	0	0	0	0
Nausea						
None	18 (90)	19 (95)	37 (92.5)	15 (78.9)	14 (70)	29 (74.4)
Mild	2 (10)	1 (5)	3 (7.5)	3 (15.8)	5 (25)	8 (20.5)
Moderate	0	0	0	1 (5.3)	1 (5)	2 (5.1)
Severe	0	0	0	0	0	0
Temperature						
None	20 (100)	19 (95)	39 (97.5)	17 (89.5)	9 (45)	26 (66.7)
Mild	0	1 (5)	1 (2.5)	2 (10.5)	8 (40)	10 (25.6)
Moderate	0	0	0	0	2 (10)	2 (5.1)
Severe	0	0	0	0	1 (5)	1 (2.6)
Any Systemic Symptom						
None	12 (60)	7 (35)	19 (47.5)	3 (15.8)	2 (10)	5 (12.8)
Mild	8 (40)	9 (45)	17 (42.5)	14 (73.7)	7 (35)	21 (53.8)
Moderate	0	4 (20)	4 (10)	2 (10.5)	10 (50)	12 (30.8)
Severe	0	0	0	0	1 (5)	1 (2.6)

### Vaccine-specific Antibody Responses

**Figure 2:**
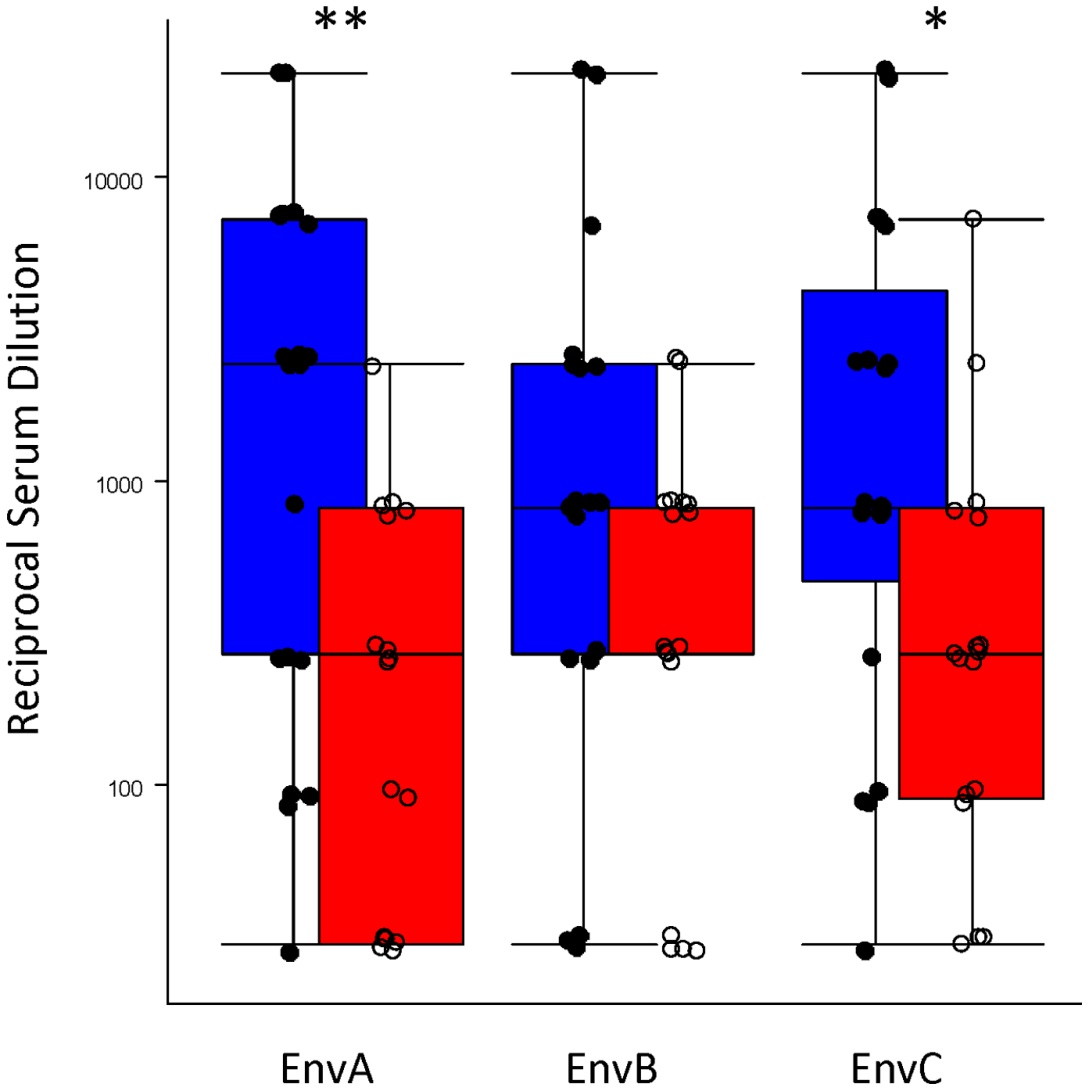
Env-specific antibody response is improved by DNA priming with Biojector®. Antibody response was measured by ELISA against HIV-1 envelope (Env) proteins matching the vaccine antigen. Data from 4 weeks post rAd5 boost is shown as individual data points and boxplots showing the median, 25^th^ and 75^th^ quartiles. Blue represents Biojector®-primed subjects and red represents subjects primed by N/S. Subjects from both rAd5 dose levels are plotted together because there was not a statistically significant effect from the booster dose. * = p<.05; ** = p<.01; *** = p<.001 (Wilcoxon Rank Sum test).

Antibody responses as measured by the research ELISA showed similar responses against EnvA, EnvB and EnvC subtypes. All 19 subjects primed with DNA by Biojector® had a positive antibody response that was on average 10-fold higher (median 2430 EnvA, range 30–50,000) 4 weeks post rAd5 boost than the 15 of 17 subjects primed with DNA by N/S who had positive responses (median 180 EnvA, range 15–2430) (Figure 2). There was no significant neutralizing activity against Tier 2 HIV isolates induced, which is consistent with prior studies [14]. The increased magnitude of the HIV-specific antibody response was reflected in the frequency and duration of VISP/R. Serology for HIV by the Abbot kit was positive at one or more timepoints in 36 of 40 participants through week 42, while HIV PCR remained negative in all cases (Table 4). At 4 weeks after the 3rd DNA vaccination, 6/19 (32%) of the subjects in the Biojector® group and 0/19 (0%; p = .02) in the N/S group had a vaccine-induced reactive EIA (two subjects have missing data). At 6 weeks after rAd5 administration (Week 30), among these 38 subjects, 19/19 (100%) in the Biojector® group and 17/19 (89.5%) in the N/S group had a reactive EIA. With regard to HIV-1 Western blot results, in the Biojector® group, there were 8 positive, 7 indeterminate and 4 unreadable, while in the N/S group there was 3 positive, 13 indeterminate and 1 unreadable test.

**Table 4:** Vaccine-Induced Sero-Positivity/Sero-Reactivity (VISP/R) through Study Week 42.

DNA administration device	rAd5 dose	Number of Subjects	EIA Negative (−)	EIA Positive (+)	WB Negative (−)	WBUninterpretable	WBIndeterminate	WBPositive (+)	*WBN/A	RNA PCRNegative (−)	RNA PCRPositive (+)
Needle	10^10^	10	3	7	0	0	6	1	3	10	0
Needle	10^11^	10	0	10	0	1	7	2	0	10	0
Biojector	10^10^	10	1	9	0	4	4	1	1	10	0
Biojector	10^11^	10	0	10	0	0	3	7	0	10	0

Results are counted as positive (or reactive) if these were the results at any time from after first vaccination through study week 42.

* Western blot (WB) was done only if the EIA was positive (reactive). HIV-uninfected status was confirmed by negative RNA PCR.

### Peptide-based Diagnostic Serology

Because of the high frequency of VISP/R and ongoing development of this vaccine platform, ancillary studies were done exploring the utility of peptide-based recombinant diagnostic test kits. The SELECTEST [24] and multiple versions of the GS HIV-1/HIV-2 PLUS O EIA (PLUS O, BioRad Laboratories) were evaluated. While these assays had excellent sensitivity for detecting HIV-infected individuals, the frequency of VISP/R was much lower. Using week 30 samples (six weeks after rAd5 boosting) only 1 subject out of 38 demonstrated VISP/R with either PLUS O or the SELECTEST screening.

### Vaccine-induced T-cell Responses

**Figure 3:**
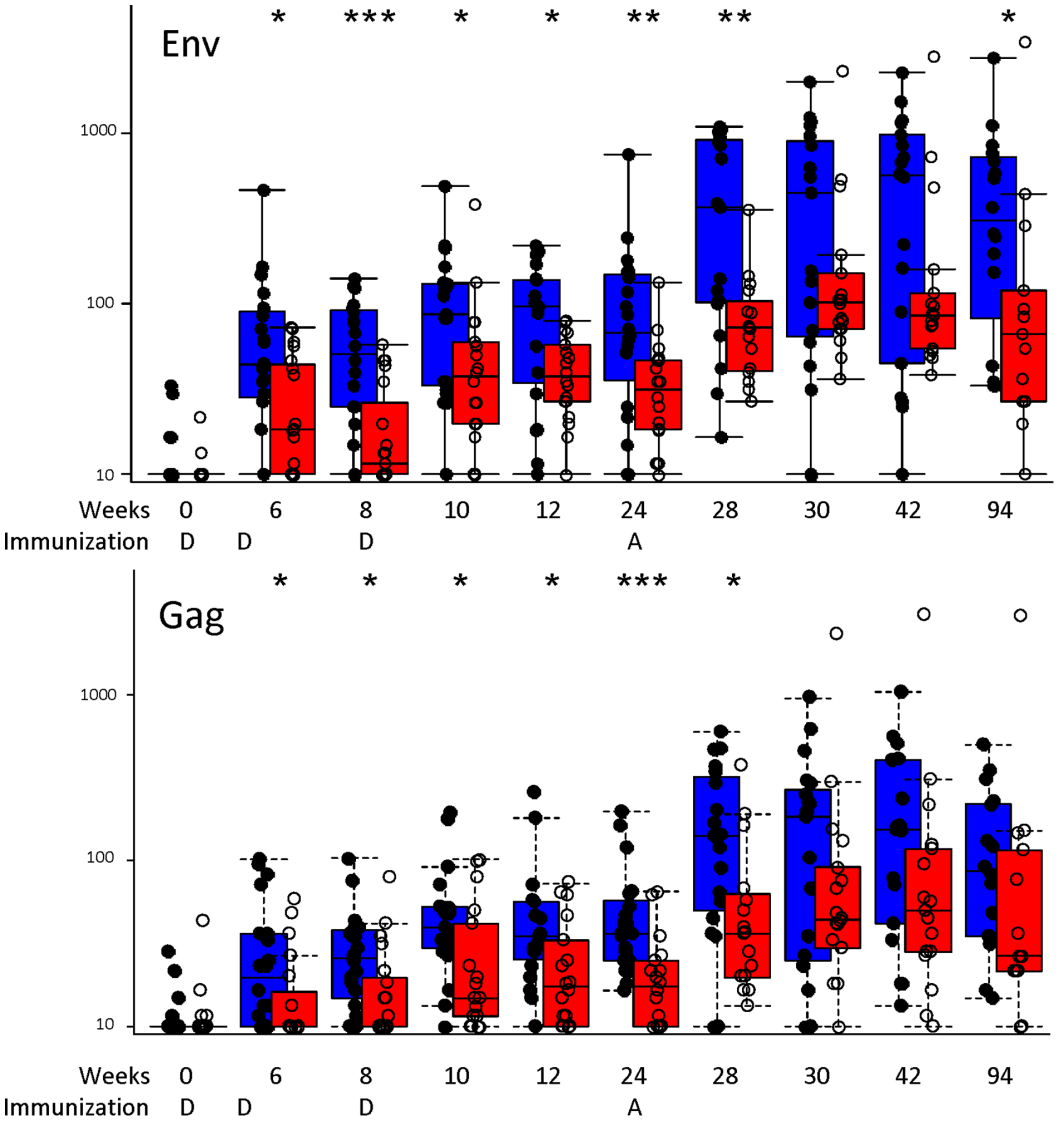
IFN-γ ELISpot responses are improved by DNA priming with Biojector®. The boxplots represent a side-by-side comparison of the median magnitude, 25^th^ and 75^th^ quartiles, for IFN-γ ELISpot responses [spot-forming units per million peripheral blood mononuclear cells (PBMCs)] in Biojector®- (blue) and N/S- (red) primed groups at baseline (Day 0), after two DNA injections (Weeks 6 and 8), post prime (Weeks 10, 12 and 24) and post rAd5 boost (Weeks 28, 30, 42, and 94). The response to EnvA and Gag peptide pools are shown. DNA “D” (weeks 0, 4, and 8) and rAd5 “A” (week 24) study injection timepoints and the scale in days are noted on the X-axis. Subjects from both rAd5 dose levels are plotted together. * = p<.05; ** = p<.01; *** = p<.001 (Wilcoxon Rank Sum test).

After the DNA prime, there was a modest, but not statistically significant, higher cumulative median ELISpot response (sum of Gag+Pol+Nef+highest Env responses), in the Biojector® compared to N/S groups. However, post rAd5 boost (Week 28), the cumulative median ELISpot response was significantly higher (p = 0.02) in the Biojector® compared to N/S primed groups. The frequency of IFN-γ ELISpot responses for any peptide pool at 4 weeks post rAd5 boost was 17/19 (89%) for the Biojector® group and 13/17 (76%; p = .73) for the N/S group (Figure 3). There was one individual with a high pre-existing background EnvA-specific ELISpot response in the N/S primed group.

CD4 T-cell responses post-boost were not statistically different in frequency (13/19 Biojector® vs. 10/17 N/S; p = .73) or magnitude. The one subject with high pre-existing EnvA ELISpot response, also had a high background pre-existing EnvA-specific CD4 T-cell response by ICS. The Biojector®-primed group had a slightly higher magnitude of CD4 T-cell responses by ICS to all peptide pools after the 3^rd^ DNA immunization (p = 0.03) that was not sustained (Figure S1).

**Figure 4:**
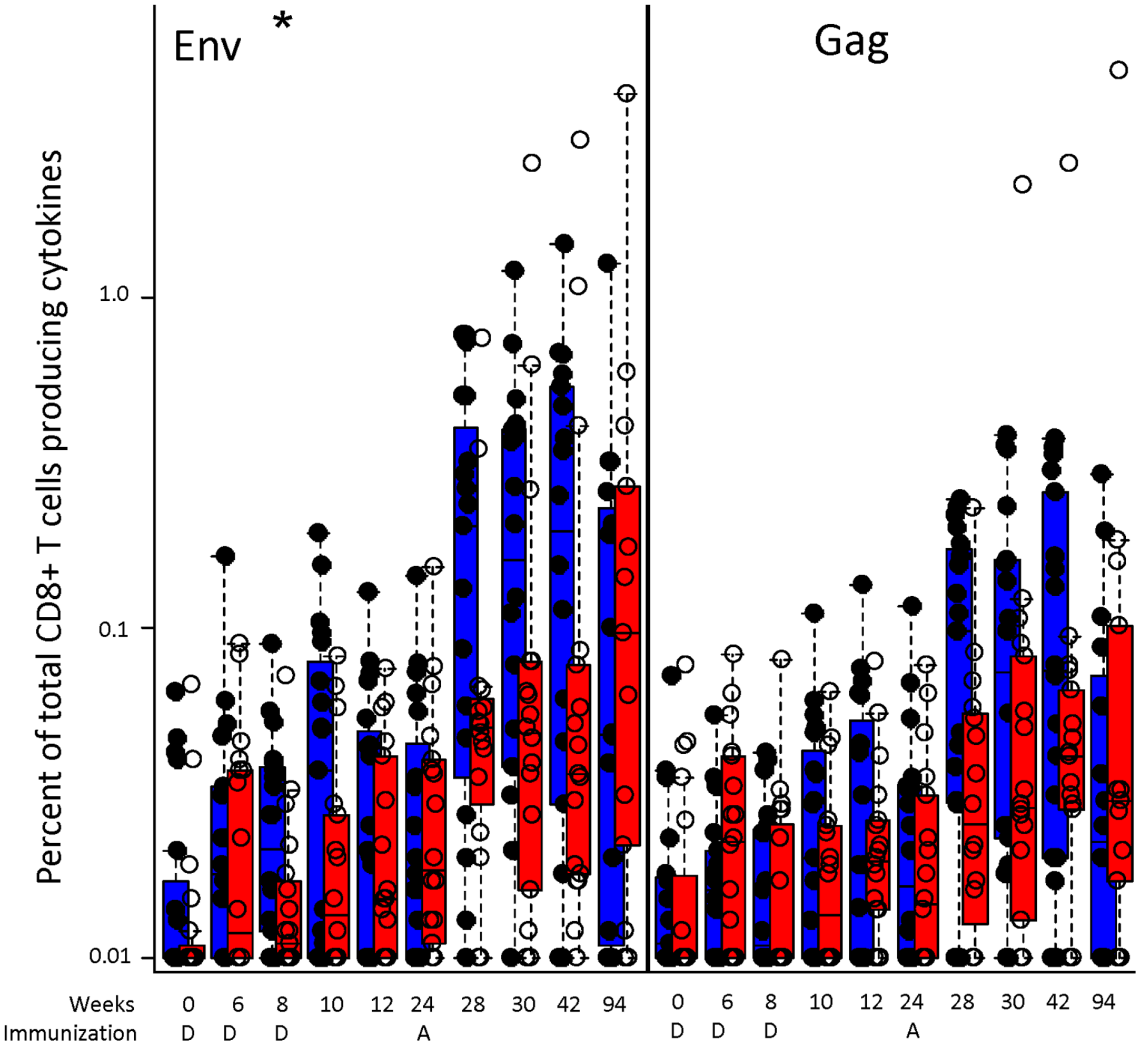
Intracellular cytokine production in CD8 T cells is improved by Biojector® delivery. The boxplots represent a side-by-side comparison of the median magnitude, 25^th^ and 75^th^ quartiles, for CD8 T cell intracellular cytokine staining in Biojector®- (blue) and N/S- (red) primed groups at baseline (Day 0), after two DNA injections (Weeks 6 and 8), post prime (Weeks 10, 12 and 24) and post rAd5 boost (Weeks 28, 30, 42, and 94). The response to EnvA and Gag peptide pools are shown. DNA (weeks 0, 4, and 8) and rAd5 (week 24) study injection timepoints are noted on the X-axis as in Figure 3. Subjects from both rAd5 dose levels are plotted together. * = p<.05; ** = p<.01; *** = p<.001 (Wilcoxon Rank Sum test).

CD8 T-cell responses post-boost displayed a similar pattern as the ELISpot responses with a higher frequency of responders among the Biojector®-primed subjects (16/19 Biojector® vs, 8/17 N/S; p = .03) and a higher magnitude at 4 weeks post rAd5 boost (p = 0.03). At 18 months after the rAd5 boost (d658) the responses were sustained, but more variable, and at that time point there was not a statistical difference between the two DNA delivery approaches (Figure 4).

## Discussion

The lack of vector-specific immunity, ease of manufacturing, and stability of plasmid DNA makes it an ideal vaccine platform. The current study helps to elucidate whether delivery by Biojector® is a factor in the immunogenicity elicited by DNA vaccines. We compared DNA priming by Biojector® vs. N/S followed by rAd5 boosting using the candidate HIV vaccine currently being evaluated in a Phase IIb clinical trial. We found that Biojector® delivery of DNA makes a significant contribution to the immunogenicity of this product. In addition, we found that despite the high frequency and magnitude of immune responses, using peptide/recombinant-based HIV enzyme immunoassay assays as opposed to bead-based methods, the problem of VISP/R could be largely avoided.

Historically, administration of injectable vaccines has been primarily accomplished with a needle and syringe (N/S). Mass production makes these supplies economical and they are a standard commodity in virtually all health care settings. There is little risk if sterile disposable supplies are used and disposed of properly, although at times, an acknowledged problem in some developing countries has been the reuse of non-sterile needles or syringes with transmission of blood-borne pathogens [26]. In all settings, the risk of needle-stick injuries and proper needle disposal remains a concern.

Needle-free injection systems are an alternative to N/S injections of vaccines. The Biojector® used in these studies ejects fluid through a small orifice under pressure to deliver liquid vaccines parenterally. While deposition may be controlled to be primarily intramuscular (IM), subcutaneous, or intradermal, IM injection delivers the injectate to all layers of the skin and subcutaneous tissues as it is propelled into muscle in a conical distribution. It has been speculated that needle-free injection devices may improve immunogenicity because of broader dispersion of the injectate than N/S [27]. A study in guinea pigs showed that Biojector® delivery compared to N/S increased the uptake of DNA plasmids in muscle and skin cells near the injection site [28]. Vaccine delivery with needle-free devices has also been reported to have an association with increased local inflammation and may enhance immunogenicity through recruitment of immunocompetent inflammatory cells [12], [13], [29]–[32]. Although the basic mechanisms by which Biojector® delivery improves the potency of DNA vaccination are unknown, it is consistent with increased transfection frequency and greater antigen production. This could be related to the ballistic nature of the injection or due to the dispersal pattern that essentially increases the number of cells exposed to DNA molecules compared to needle injection in which much of the injectate pools in the needle track.

DNA has been delivered by other needle-free devices in clinical trials. The Powderject™ system uses compressed helium to project DNA in the form of dry powder through the skin. Another device, the “gene gun”, was developed exclusively for DNA delivery. It uses compressed helium to shoot nanoparticle-sized gold balls coated with DNA into the skin. DNA immunization by either device has elicited immune responses, but they have not been compared directly to N/S delivery, and neither device is currently licensed. Biojector® is a hand-held, portable, easy to use, FDA-approved device for IM delivery of clinical products, including injections for children. Other approaches are being developed to improve DNA vaccine transfection efficiency. Electroporation has been shown to significantly improve the immunogenicity of DNA vaccines in nonhuman primates [33], although the results in human studies to date have been modest [34].

In this study, Biojector® delivery of DNA caused a slightly greater reactogenicity than N/S delivery but the events were generally mild, well tolerated, and often unnoticed by the subject. Importantly, delivery of DNA vaccines by the needle-free Biojector® device was associated with improved antibody, IFN-γ ELISpot, and CD8+ T cell responses post boosting with rAd5. This was associated with subtle differences in CD4 and CD8 T cell responses post DNA priming that were significantly amplified post rAd5 boosting as previously reported [14]. These data support continued clinical evaluation of DNA vaccines using Biojector® delivery, and suggest that transfection efficiency is a key factor in improving the potency of DNA vaccines.

## Supporting Information

Figure S1 Intracellular cytokine production in CD4 T cells. The boxplots represent a side-by-side comparison of the median magnitude, 25^th^ and 75^th^ quartiles, for CD4 T cell intracellular cytokine staining in Biojector®- (blue) and N/S- (red) primed groups at baseline (Day 0), after two DNA injections (Weeks 6 and 8), post prime (Weeks 10, 12 and 24) and post rAd5 boost (Weeks 28, 30, 42, and 94). The response to EnvA and Gag peptide pools are shown. DNA (weeks 0, 4, and 8) and rAd5 (week 24) study injection timepoints are noted on the X-axis as in Figure 3. Subjects from both rAd5 dose levels are plotted together. * = p<.05; ** = p<.01; *** = p<.001 (Wilcoxon Rank Sum test).
(PPTX)

Checklist S1 **CONSORT Checklist.**
(DOC)

Protocol S1 **Trial Protocol.**
(PDF)
