# Supplementary material for: DNA Vaccine Delivered by a Needle-Free Injection Device Improves Potency of Priming for Antibody and CD8+ T-Cell Responses after rAd5 Boost in a Randomized Clinical Trial
Source: PLoS One. 2013 Apr 8;8(4):e59340. doi: 10.1371/journal.pone.0059340 (PMC3620125; doi:10.1371/journal.pone.0059340)
Supplement: Figure S1 — Intracellular cytokine production in CD4 T cells. The boxplots represent a side-by-side comparison of the median magnitude, 25th and 75th quartiles, for CD4 T cell intracellular cytokine staining in Biojector®- (blue) and N/S- (red) primed groups at baseline (Day 0), after two DNA injections (Weeks 6 and 8), post prime (Weeks 10, 12 and 24) and post rAd5 boost (Weeks 28, 30, 42, and 94). The response to EnvA and Gag peptide pools are shown. DNA (weeks 0, 4, and 8) and rAd5 (week 24) study injection timepoints are noted on the X-axis as in Figure 3. Subjects from both rAd5 dose levels are plotted together. * = p<.05; ** = p<.01; *** = p<.001 (Wilcoxon Rank Sum test). (PPTX) [file pone.0059340.s001.pptx]

## Slide 1
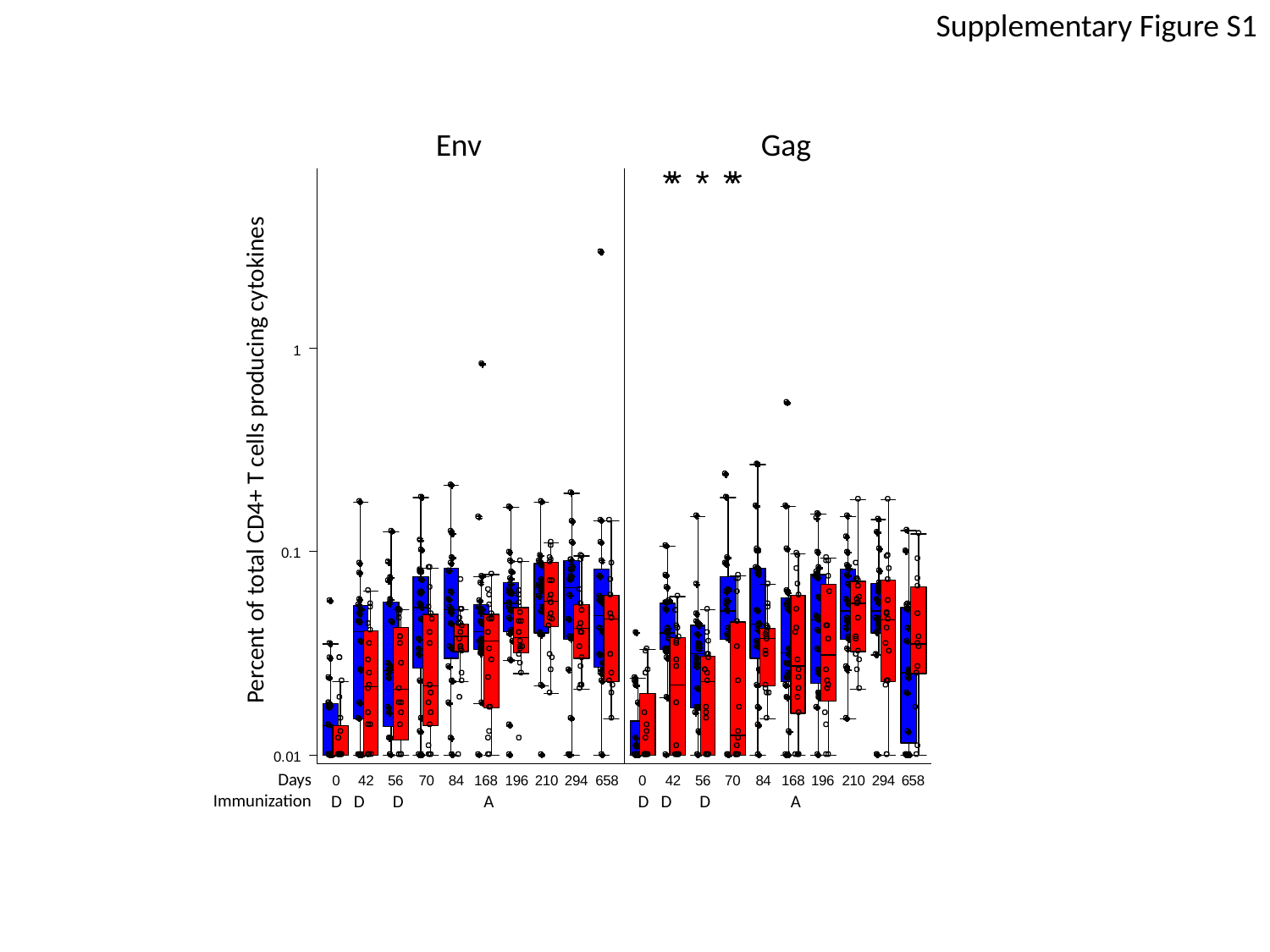

Supplementary Figure S1
Env
Gag
Percent of total CD4+ T cells producing cytokines
Days
Immunization
D D D A
D D D A
