# Supplementary material for: DNA Vaccine Delivered by a Needle-Free Injection Device Improves Potency of Priming for Antibody and CD8+ T-Cell Responses after rAd5 Boost in a Randomized Clinical Trial
Source: PLoS One. 2013 Apr 8;8(4):e59340. doi: 10.1371/journal.pone.0059340 (PMC3620125; doi:10.1371/journal.pone.0059340)
Supplement: Protocol S1 — Trial Protocol. (PDF) [file pone.0059340.s003.pdf]

Version 3.0
November 9, 2005

VACCINE RESEARCH CENTER

Protocol VRC 008
(05-I-0148)

A Phase I Clinical Trial of a Prime-Boost HIV-1Vaccination Schedule: Multiclade DNA Vaccine, VRC-HIVDNA016-00-VP, Followed by Multiclade Adenoviral Vector Vaccine, VRC-HIVADV014-00-VP, in Uninfected Adult Volunteers

Vaccines Provided by
Vaccine Research Center/NIAID/NIH, Bethesda, MD

Clinical Trial Sponsored by:
National Institute of Allergy and Infectious Diseases (NIAID)
Vaccine Research Center (VRC)
Bethesda, Maryland

IND Sponsored by:
National Institute of Allergy and Infectious Diseases
Division of AIDS (DAIDS)
Bethesda, Maryland

BB IND 12326 - *held by DAIDS*

Principal Investigator:
Andrew Catanzaro, M.D.
Vaccine Research Center, National Institute of Allergy and Infectious Diseases (NIAID)
National Institutes of Health (NIH)
Bethesda, MD 20892

IRB Initial Review Date: March 21, 2005
VRC Protocol: 008

TABLE OF CONTENTS

Page
Précis 5

1. INTRODUCTION AND RATIONALE 7

1.1 HIV-1: Etiology, Disease Course, and Epidemiology 7
1.2 Rationale for Development of a Prime-Boost Regimen 7
1.3 Previous Human Experience with VRC-HIVDNA016-00-VP and a Similar HIV DNA Plasmid Vaccine Developed by VRC 8
1.4 Previous Human Experience with VRC-HIVADV014-00-VP........................ 12
1.5 Rationale for Evaluating Devices for IM Injection of DNA Vaccine 15
1.6 Considerations Related to Pre-Existing Antibody Titer to Adenovirus Serotype 5 17
1.7 Measures of Immunogenicity 18

2. BACKGROUND ON VACCINE.. 18

2.1 HIV-1 DNA Vaccine Plasmids in VRC-HIVDNA016-00-VP........................ 18
2.2 Adenoviral Vectors in VRC-HIVADV014-00-VP........................ 20
2.3 Preparation of the Bulk Plasmid and Final Product........................ 21
2.3.1 VRC-HIVDNA016-00-VP 21
2.3.2 VRC-HIVADV014-00-VP........................ 21
2.4 Preclinical Studies........................ 21
2.4.1 Preclinical Biodistribution Studies in Rabbits........................ 22
2.4.2 Preclinical Toxicology Studies of Prime-Boost Regimens in Rabbits 22
2.4.3 Preclinical Safety and Immunogenicity of DNA Vaccination with Administration by Biojector or Needle and Syringe 24
2.4.4 Immune Responses to VRC-HIVDNA009-00-VP Prime Followed by VRC-HIVADV014-00-VP Boost in Cynomolgus Macaques (VRC-02-035)........................ 25
2.5 Release Criteria........................ 25

3. STUDY OBJECTIVES.. 26

3.1 Primary Objective 26
3.2 Secondary Objectives........................ 26
3.3 Exploratory Objectives 26

4. STUDY DESIGN.. 27

4.1 Study Population........................ 27
4.1.1 Inclusion Criteria........................ 28
4.1.2 Exclusion Criteria........................ 29
4.2 Schedule of Clinical Procedures and Laboratory Assays 31
4.2.1 Screening........................ 31
4.2.2 Day 0 through Week 42 Clinical Follow-Up and Week 94 Follow-up........................ 32
4.3 Monitoring for HIV Infection........................ 35
4.4 Intercurrent HIV Infection........................ 36
4.5 Concomitant Medications........................ 36
4.6 Criteria for Withdrawal of a Subject from Injection Schedule........................ 36
4.7 Criteria for Stopping Study (as Amended November 9, 2005) 37

5. SAFETY AND ADVERSE EVENT REPORTING 38

5.1 Adverse Events 38
5.2 Serious Adverse Events (SAE) 39
5.3 Adverse Event Reporting to the IND Sponsor 39
5.4 Adverse Event Reporting to the Institutional Review Board 40
5.5 Serious Adverse Event Reporting to the Institutional Biosafety Committee 41

6. STATISTICAL CONSIDERATIONS 41

6.1 Overview 41
6.2 Objectives 41
6.3 Endpoints 41
6.3.1 Safety 41
6.3.2 Immunogenicity 42
6.3.3 Social Impacts 42
6.4 Sample Size and Accrual 42
6.4.1 Randomization of Treatment Assignments 42
6.4.2 Power Calculations for Safety 43
6.4.3 Sample Size Calculations for Immunogenicity 44
6.5 Statistical Analysis 45
6.5.1 Analysis Variables 45
6.5.2 Baseline Demographics 46
6.5.3 Safety Analysis 46
6.5.4 Immunogenicity Analysis 46
6.5.5 Social Impact analysis 47
6.5.6 Interim analyses 47

7. PHARMACY PROCEDURES 47

7.1 Study Agents for Prime-Boost Regimen 47
7.1.1 DNA 6-Plasmid Vaccine, VRC-HIVDNA016-00-VP 47
7.1.2 Adenoviral Vector Vaccine, VRC-HIVADV014-00-VP 48
7.2 Study Agent Administration 49
7.2.1 Administration of VRC-HIVDNA016-00-VP by Biojector 49
7.2.2 Administration of VRC-HIVDNA016-00-VP by Needle and Syringe 49
7.2.3 Administration of VRC-HIVADV014-00-VP by Needle and Syringe 49
7.3 Study Agent Labeling 50
7.4 Procedures to Preserve Blinding 50
7.5 Study Agent Accountability 50
7.5.1 Documentation 50
7.5.2 Disposition 50

8. HUMAN SUBJECT PROTECTIONS AND ETHICAL OBLIGATIONS 51

8.1 Informed Consent 51
8.2 Risks and Benefits 51
8.2.1 Risks 51
8.2.2 Benefits 53
8.3 Institutional Review Board 53
8.4 Protocol Registration 53
8.5 Subject Confidentiality 54

8.6 Plan for Use and Storage of Biological Samples 54
8.7 Subject Identification and Enrollment of Study Participants 54
8.7.1 Participation of Children 54
8.8 Compensation 54
8.9 Safety Monitoring 55

9. ADMINISTRATIVE AND LEGAL OBLIGATIONS 55

9.1 Protocol Amendments and Study Termination 55
9.2 Study Documentation and Storage 55
9.3 Study Monitoring, Data Collection and Data Monitoring 56
9.3.1 Study Monitoring 56
9.3.2 Data Collection 56
9.4 Language 56
9.5 Policy Regarding Research-Related Injuries 56

10. REFERENCES 57

APPENDICES

I Study Informed Consent Form 61
II Contact Information 79
III Schedule of Evaluations 81
IV Table for Grading Severity of Adverse Events 84
V Skin Biopsy Informed Consent Form 86

Précis

Protocol VRC 008: A Phase I Clinical Trial of a Prime-Boost HIV-1Vaccination Schedule: Multiclade DNA Vaccine, VRC-HIVDNA016-00-VP, Followed by Multiclade Adenoviral Vector Vaccine, VRC-HIVADV014-00-VP, in Uninfected Adult Volunteers

Study Design: This is a Phase I randomized study to examine safety and tolerability of, as well as immune response to, a schedule of 3 HIV DNA plasmid vaccinations followed by one HIV adenoviral vector vaccine (rAd) booster. The hypotheses are that: 1) this regimen will be safe for human administration and elicit immune responses to HIV-1; 2) Biojector and needle/syringe are both safe to use for IM injection of the DNA vaccine and 3) subjects with both low and high pre-existing adenovirus serotype 5 antibody (Ad5Ab) titer will have a boost in immune response to HIV-1 peptides following the Ad booster vaccination. In this study equal numbers of subjects with high and low Ad5Ab titers will be randomized to receive DNA vaccinations by either needle and syringe (N/S) or by Biojector and then to receive either 10^{10} PU or 10^{11} PU rAd booster vaccination in a factorial design. The primary objective is to evaluate the safety and tolerability in humans of the prime-boost vaccination regimen. Secondary objectives are related to evaluation of the immunogenicity of the DNA vaccine when administered by N/S or Biojector, the immunogenicity of the Ad vaccine at two different doses in subjects with high and low pre-enrollment titers of Ad5Ab, the development of adenovirus serotype 5 neutralizing antibody and the social impact of participating in an HIV-1 vaccine trial. Exploratory evaluations of the immunogenicity of the prime-boost regimen are also planned. The preliminary results may serve as the basis for designing studies to provide more definitive answers to questions about method of administration and effect of pre-enrollment Ad5Ab titer on safety of and immune response to the rAd booster vaccination.

Product Description: VRC-HIVDNA016-00-VP is composed of 6 closed, circular DNA plasmids that are each 16.67% (by weight) of the vaccine. Each of the 6 plasmids in this vaccine expresses a single gene product. Plasmids VRC 4401, VRC 4409 and VRC 4404 are designed to express clade B HIV-1 Gag, Pol and Nef, respectively. VRC 5736, VRC 5737, and VRC 5738 are designed to express HIV-1 Env glycoprotein from clade A, clade B, and clade C, respectively. Vaccine vials will be supplied at 4 mg/mL. DNA vaccinations will be 1 mL of vaccine administered intramuscularly using either N/S or the Biojector 2000 Needle-Free Injection Management System®. VRC-HIVADV014-00-VP is a recombinant product composed of four non-replicating adenoviral vectors (in a 3:1:1:1 ratio) that code for HIV-1 Gag/Pol polyproteins from clade B and HIV-1 Env glycoproteins from clades A, B, and C. All rAd injections will be administered by N/S.

Subjects: Forty healthy adult volunteers, 18 to 50 years old; 20 subjects with low Ad5Ab (≤1:500) and 20 subjects with high Ad5Ab (>1:500).

Study Plan: Forty subjects will be randomized in a 1:1 ratio to receive the same vaccination schedule but by two different methods of intramuscular administration (N/S or Biojector), as shown in the schema. The rAd boost will also be randomized to be either 10^{10} PU or 10^{11} PU in 1:1 ratio. The rAd boost dosage will be blinded until 6 weeks of safety and immunogenicity evaluations after the rAd boost are completed for all subjects.

**VRC 008**		**6-plasmid DNA prime vaccinations (At least 21 days between vacs.)**				**rAd booster vaccination**	
**Subject Description**	**Number**	**DNA Vac Device**	**Day 0**	**Day 28±7**	**Day 56±7**	**Number**	**Day 168 (-7, +14 days)**
Subjects with pre-enrollment low Ad5Ab titer (≤1:500)	10	Biojector	4 mg	4 mg	4 mg	5	10^{10} PU
						5	10^{11} PU
	10	N/S	4 mg	4 mg	4 mg	5	10^{10} PU
						5	10^{11} PU
Subjects with pre-enrollment high Ad5Ab titer (>1:500)	10	Biojector	4 mg	4 mg	4 mg	5	10^{10} PU
						5	10^{11} PU
	10	N/S	4 mg	4 mg	4 mg	5	10^{10} PU
						5	10^{11} PU

Sample Size: 40 subjects:

Biojector DNA vaccine primes: N = 20
Needle and Syringe DNA vaccine primes: N=20
10^{10} PU rAd booster vaccination: N=20
10^{11} PU rAd booster vaccination: N=20

Study Duration: Each subject will require 42 weeks on study to complete the prime-boost regimen and clinical follow-up. A long-term follow-up one year later (week 94) will be encouraged to allow HIV testing, long-term immunology evaluation and interview about significant health changes. However, subject may opt for long-term contact to occur by telephone, e-mail or mail for the interview only.

Study Endpoints: The primary endpoint is safety of the prime-boost vaccination regimen; secondary endpoints are cellular immune responses through 4 weeks after the 3rd DNA vaccine and through 6 weeks after the rAd booster, the Ad5 neutralizing antibody titer through 4 weeks after the rAd booster, and social impact of participating in an HIV vaccine study. Exploratory analyses will include assays for cellular and humoral immunity at intervals between Day 0 and Week 42 and at Week 94 for subjects who opt to return for a long-term follow-up blood draw.

1. INTRODUCTION AND RATIONALE

1.1 HIV-1: ETIOLOGY, DISEASE COURSE, AND EPIDEMIOLOGY

The Centers for Disease Control and Prevention (CDC) estimate that in the United States, 850,000 to 950,000 people are living with human immunodeficiency virus (HIV) infection and approximately 25% are unaware of their infection [1]. Worldwide, the rate of new HIV infections continues to increase. Although new acquired immunodeficiency syndrome (AIDS) diagnoses and deaths have fallen significantly in developed countries since the advent of highly active antiretroviral therapy (HAART), in the developing world the HIV/AIDS epidemic continues to accelerate. The global impact of the epidemic is staggering. According to the Joint United Nations Programme on HIV/AIDS and the World Health Organization, as of the end of 2002, 40-42 million people were estimated to be living with HIV/AIDS, with 95% of the global total residing in the developing world [2, 3]. Worldwide there will be an estimated 2.5-3.5 million deaths due to HIV/AIDS in 2003 [2] and there have been as many as 30 million deaths as a result of HIV infection since the beginning of the epidemic [3]. Beyond the human tragedy of HIV/AIDS, the costs of the epidemic pose a significant impediment to the economic growth and political stability of many countries. In developing countries and in segments of the U.S. population, anti-HIV therapies are frequently beyond financial reach. Accordingly, effective, low-cost tools for HIV prevention, such as a vaccine, are urgently needed to bring the HIV epidemic under control. For this reason, the Vaccine Research Center (VRC) and Division of AIDS (DAIDS) at the National Institute of Allergy and Infectious Diseases (NIAID) of the National Institutes of Health (NIH) are committed to the development of safe, effective vaccines to prevent HIV infection and AIDS worldwide.

The use of multivalent vaccines, containing a defined mixture of immunogens from a number of prevalent subtypes might be a feasible approach to achieve broadly-protective HIV vaccines. The World Health Organization UNAIDS HIV Vaccine Advisory Committee has recommended that candidate HIV vaccines be designed based upon the strains prevalent in the country in which trials are to be conducted [4]. The Vaccine Research Center, NIAID, NIH and the World Health Organization-Joint United Nations Programme on HIV/AIDS organized a meeting focused on the genetic diversity of HIV and strategies to develop vaccine candidates. A consensus was reached that generation of multiclade candidate vaccines is a high international scientific priority [5]. This approach is the foundation for the multiclade design of the prime-boost vaccination regimen development strategy. The DNA vaccine,VRC-HIVDNA016-00-VP, as well as the adenoviral vector vaccine, VRC-HIVADV014-00-VP encode *gag* and *pol* gene sequences from clade B as well as more diverse *env* genes from clades A, B and C. The DNA vaccine also encodes a clade B *nef* gene sequence. Clades A, B and C together represent the viral subtypes responsible for about 90% of new HIV infections in the world [6].

1.2 RATIONALE FOR DEVELOPMENT OF A PRIME-BOOST REGIMEN

Combination modality regimens using a deoxyribonucleic acid (DNA) vaccine prime followed by a viral vector boost have shown promise in non-human primate models of HIV infection. A prime-boost regimen with the same investigational vaccines that will be used in this Phase I study has been evaluated in a non-human primate model and shows promising immunogenicity. These results are summarized in protocol Section 2.4. Through a series of previous preclinical studies with similar predecessor vaccines, the constructs used in the DNA vaccine and the rAd

vaccine have been refined for improved *in vivo* expression and immunogenicity.

Prime-boost vaccination regimens have the potential for raising high levels of immune responses in non-human primate models of HIV infection. Published examples of this strategy include DNA vaccine priming followed by a recombinant viral vector boost with a modified vaccinia Ankara (rMVA) [7] or a replication-deficient recombinant adenoviral vector vaccine (Ad5) [8]. These studies have shown that the regimen can attenuate a pathogenic SHIV infection in rhesus macaques, most likely by the generation of a $CD8^+$ cytotoxic T lymphocyte (CTL) response.

Merck Research Laboratories has published preclinical studies of priming for HIV-1 specific immunity using an adjuvant-formulated DNA vaccine followed with Ad5 vaccine boost. This generates levels of T-cell immune response that are comparable to those in naive animals receiving multiple high doses of Ad5 HIV-1 vaccines [9].

A prime-boost regimen has also shown promise in a preclinical model for prevention of Ebola virus infection in a study sponsored by the VRC, NIH and the Special Pathogens Branch, CDC. Cynomolgus macaques immunized with a combination DNA plasmid (a mixture of four DNA plasmids encoding glycoproteins from three Ebola strains and nucleoprotein from one strain) and boosted with a replication-deficient adenoviral vector encoding the glycoprotein resisted lethal viral challenge [10].

These preclinical studies suggest that DNA plasmid vaccine priming, followed by replication-defective adenoviral vector vaccine boost, can elicit potent and protective T-cell immune responses. This prime-boost vaccine strategy may be successful in humans to control HIV-1 and other viral infections.

1.3 Previous Human Experience with VRC-HIVDNA016-00-VP and a Similar HIV DNA Plasmid Vaccine Developed by VRC

The DNA vaccine that will be administered in VRC 008 is VRC-HIVDNA016-00-VP. It is composed of 6 closed, circular deoxyribonucleic acid (DNA) plasmids that are each 16.67% (by weight) of the vaccine. DNA plasmids VRC 4401, VRC 4409 and VRC 4404 are designed to express clade B HIV-1 Gag, Pol and Nef, respectively. DNA plasmids VRC 5736, VRC 5737, and VRC 5738 are designed to express HIV-1 Env glycoprotein from clade A, clade B, and clade C, respectively. This DNA vaccine was designed to incorporate the safety features of previous VRC HIV-1 vaccines, while improving the immunogenicity.

The rationale in development of VRC-HIVDNA016-00-VP was to separate the *gag, pol* and *nef* genes into separate plasmids, rather than having one plasmid that produces a fusion protein immunogen, as was the case with the 4-plasmid vaccine, VRC-HIVDNA009-00-VP. Small changes to the plasmids are expected to enhance the immunogenicity of the protein products produced *in vivo*. In addition to splitting the genes into separate plasmids there are two other changes in the plasmid construction. These are: 1) a change in the promoter incorporated into these plasmids and 2) a 68 amino acid addition to the *gag* gene in the VRC 4401 (Gag protein only) plasmid as compared to the VRC 4306 (Gag-Pol-Nef fusion protein) plasmid that was in VRC-HIVDNA009-00-VP. Preclinical data with VRC-HIVDNA016-00-VP indicate that the separate *gag, pol* and *nef* plasmids generate more consistent and stronger immune responses to the Gag, Pol and Nef immunogens that are produced. The clades A, B and C Env plasmids in VRC-HIVDNA016-00-VP are identical to the three Env plasmids in VRC-HIVDNA009-00-VP except that the promoter known as CMV/R has been used rather than the CMV promoter. The

CMV/R promoter used is identical to one currently under evaluation in a DNA plasmid Ebola vaccine (BB-IND 11294).

The VRC, NIAID, NIH in collaboration with DAIDS, NIAID, NIH has previously sponsored clinical trials with two different multiclade HIV-1 DNA vaccines. These are a multiclade 4-plasmid DNA vaccine (VRC-HIVDNA009-00-VP) and the multiclade 6-plasmid DNA vaccine (VRC-HIVDNA016-00-VP) that will be used in this study. The table below shows the status as of May 2005 of the studies in which these vaccines have been administered as single agents in healthy subjects.

Multiclade HIV DNA Vaccine Experience in Uninfected Subjects as of May 2005

<table>
<tr><th>HIV DNA vaccine formulation</th><th>Study</th><th>Dose (mg)</th><th># active doses planned (subjects in active arm)</th><th># active doses to date (subjects in active arm)</th><th>Comment</th></tr>
<tr><td rowspan="3">VRC-HIVDNA009-00-VP
4 plasmids (multiclade):
clade B gag-pol-nef,
clade A env,
clade B env,
clade C env.</td><td>VRC 004 (BB-IND 10681)</td><td>2
4
8</td><td>15 (5)
60 (20)
45 (15)</td><td>15 (5)
60 (20)
44 (15)</td><td>Vaccinations completed and study unblinded;
also included 10 placebo subjects (29 placebo injections)</td></tr>
<tr><td>HVTN 052 (BB-IND 10681)</td><td>4</td><td>300 (120)</td><td>≥292 (120)</td><td>Of 540 blinded doses (300 active and 240 placebo) planned, 8 total were not given (still blinded).</td></tr>
<tr><td>RV156 (BB-IND 10681)</td><td>4</td><td>45 (15)</td><td>~30 (15)</td><td>First enrollment January 2005; also includes 15 placebo subjects (45 placebo injections)</td></tr>
<tr><td rowspan="2">VRC-HIVDNA016-00-VP
6 plasmids (multiclade):
clade B gag,
clade B pol,
clade B nef,
clade A env,
clade B env,
clade C env.</td><td>VRC 007 (BB-IND 11750)</td><td>4</td><td>45 (15)</td><td>44 (15)</td><td>Vaccinations completed; open label; no placebos.</td></tr>
<tr><td>VRC 008</td><td>4</td><td>120 (40)</td><td>0</td><td>Initiated May 16, 2005</td></tr>
<tr><td colspan="6">Summary: A dose range of 2 mg to 8 mg was evaluated for the 4-plasmid DNA vaccine. The majority of experience is with the 4 mg dose. This dose range was well tolerated.
Phase I studies (uninfected subjects) include plans for 630 vaccine injections in 230 subjects.
As of May 5, 2005, there have been 190 uninfected subjects who have received one or more vaccine injections.

Note: Experience with a clade B single plasmid vaccine, with the 4-plasmid DNA vaccine in combination with an IL2/Ig adjuvant and with the 4-plasmid DNA vaccine in HIV-infected subjects is not shown. These studies together include more than 50 vaccinees to date.</td></tr>
</table>

VRC 004: Protocol VRC-004 (03-I-0022) is a Phase I randomized, controlled, double-blinded dose escalation study to evaluate safety, tolerability, dose and immune response of a multiclade HIV plasmid DNA vaccine identified as VRC-HIVDNA009-00-VP. This study opened to accrual in November 2002 and was fully enrolled with 50 healthy HIV-negative subjects in August 2003. Forty subjects received vaccine injections and 10 subjects received placebo injections. The three-injection schedule (administered at Weeks 0, 4 and 8), was completed in 5

of 5 subjects randomized to 2 mg vaccine injections, 20 of 20 subjects randomized to 4 mg vaccine injections, 14 of 15 subjects randomized to 8 mg vaccine injections and 9 of 10 subjects randomized to placebo injections. The unblinded final study results indicate that the vaccine injections were as well tolerated as the placebo injections.

In the vaccine groups, there were three adverse events possibly related to vaccine that required expedited reporting to the IND Sponsor. These were a grade 3 asymptomatic neutropenia with onset 27 days after 3rd vaccination (4 mg group), a grade 3 urticaria with onset 4 days after 3rd vaccination (4 mg group) and a grade 2 maculopapular rash with onset 27 days after 2nd vaccination (8 mg group). All resolved without sequelae. Other factors in the subject with urticaria included concomitant bladder infection, yeast infection and multiple antibiotics. The maculopapular rash resulted in discontinuation from the vaccination schedule after the 2nd injection and it was clinically consistent with either a drug eruption or a viral exanthem. Informed consents for protocols using this or similar HIV DNA vaccines should note the risk of neutropenia, maculopapular rash and urticaria. The diary cards indicate that vaccine injections were well tolerated. No subjects reported severe symptoms on diary cards. Most subjects (80-100% per group), including placebo recipients, reported at least one local symptom (pain/tenderness, induration or erythema) at some point in the 7 days after an injection. Most subjects (70-80% per group), including placebo recipients, also reported at least one systemic symptom in the 7 days after an injection. No vaccine recipients reported fever. Chills and nausea were infrequent in all subjects (0-20% per dose group). Headache and myalgia were reported in 20-50% per dose group. Malaise was the most common systemic symptom, occurring in 50-60% of vaccinees and 40% of placebo recipients at least once in the 7 days following a study injection. During the study the most frequently recorded laboratory adverse events included asymptomatic hyperglycemia and hypoglycemia. The unblinded data show that placebo recipients had higher incidence of both hyperglycemia and hypoglycemia and these data support the clinical impression that variations in blood glucose are unrelated to study vaccinations.

Preliminary immunogenicity data through Week 12 from the VRC 004 study, when sorted by treatment assignment indicate that $CD4^+$ responses were detected in nearly 100% of recipients at all dose levels. $CD8^+$ responses were detected in nearly half. The greatest responses (in frequency and magnitude) were generally observed as directed against Env. The immunogenicity responses to the Gag, Pol and Nef are weak to absent. There is a trend to greater responses in the 4 mg and 8 mg dose compared to the 2 mg dose, although not statistically significant. There was a statistically significant increase in the response after 3 injections compared to 2 injections at both the 4 mg and 8 mg dose levels, although there is no way to determine if this was due to the 3rd injection or simply a maturation of the response following the 2nd injection. Definitive responses are first detectable with the 4 mg and 8 mg dose at the 6-week time point (2 weeks after the second injection). When compared with the 8 mg dose, the 4 mg dose offers the combination of a good safety profile, greater ease of administration, and approximately equivalent cellular immunogenicity.

Fourteen of 35 vaccinees in the 4 mg and 8 mg groups had a positive ELISA at one or more points between Week 8 and Week 52 when tested by a commercial HIV antibody test. The optical density (O.D.) of the ELISA results usually decreased over time; six subjects were ELISA positive by the commercial assay at Week 52.

HVTN 052: The investigational vaccine, VRC-HIVDNA009-00-VP, is now undergoing further

evaluation in clinical trials sponsored by DAIDS, NIAID at extramural sites. The HIV Vaccine Trials Network (HVTN) is conducting a Phase IB study, HVTN 052 (BB-IND 10681), to evaluate the safety and immunogenicity of VRC-HIVDNA-009-00-VP in two- versus three-injection regimens in 180 participants (120 vaccine/60 control). The schedule being compared is 0, 4 and 8 weeks versus 0 and 8 weeks, and all injections are given by Biojector. The study opened for enrollment in December 2003 and accrual was completed on October 19, 2004. As of May 2005, all 180 participants are beyond Week 8; 8 of the 540 planned injections were not administered. The study remains blinded.

Local reactions of pain and/or tenderness at the injection site were reported by 158 (88%) of the participants. Mild erythema and/or induration were reported by 36% of the participants. All local reactions were mild or moderate, except in one participant, who experienced an episode of severe injection site pain that started 30 minutes after the first vaccination. The pain was mild by the following day and resolved by day 4.

Sixty percent of the participants experienced at least one mild or moderate systemic symptom (malaise, myalgia, headache, nausea, vomiting, chills or arthralgia); the majority of the symptoms (83%) were mild. One participant reported a severe migraine headache.

At 16 days after the 2nd injection, a 21-year-old male had an elevated alanine aminotransferase (ALT) (221 U/L), aspartate aminotransferase (AST) (523 U/L), and creatinine phosphokinase (CPK) (30727 U/L). All other laboratory and clinical evaluations were within normal limits and the subject reported feeling well. He reported exercising strenuously three times in the previous week, had consumed alcohol on 3 of the previous 5 evenings, and had taken 1115 mg acetaminophen with codeine four days prior to the visit. The lab values returned to normal limits within a week. The elevated CPK, AST and ALT were attributed to exercise and assessed as unrelated to the study vaccine. To date, HVTN 052 also supports the finding in VRC 004 that the 4 mg dosage of the vaccine is well tolerated and safe in healthy volunteers.

VRC 007: VRC 007 (04-I-0254) is the first Phase I study of the 6-plasmid DNA vaccine, VRC-HIVDNA016-00-VP. This open-label study enrolled 15 subjects between August 17, 2004 and October 28, 2004. Fourteen of the 15 subjects received 3 intramuscular injections of a 4 mg dose of vaccine administered by Biojector; one subject was lost to follow-up after two vaccinations. The last study vaccination was administered on December 22, 2004. This summary represents interim results through May 4, 2005. No subjects reported fever following vaccination. Reactogenicity was none to mild except that two subjects reported moderate injection site pain and one subject reported moderate nausea and malaise. The only adverse event requiring expedited reporting to the IND sponsor was a grade 3 generalized urticaria. The subject had reported starting an antihistamine about 2 weeks after first vaccination but reported at that time that the reason was latex allergy. While being screened for the rollover booster study, VRC 010, it was learned that the subject had experienced generalized urticaria around the time of the second vaccination when the supply of antihistamine ran out. As of May 2005 the subject has chronic urticaria that are well controlled by antihistamine. Evaluation is ongoing. The etiology is unknown but at this time the chronic urticaria are assessed as possibly related to study vaccine. To date, there have been two moderate (grade 2) adverse events possibly attributed to vaccine. These were intermittent dizziness of 2 days duration beginning 13 days after the second vaccination in one subject (this subject received the third vaccination without recurrence of symptoms) and asymptomatic hypoglycemia in another subject, first noted at the follow-up visit that was 14 days after the third vaccination. The last safety evaluation of the subject lost to

follow-up was by telephone one day after the second vaccination; at that time the subject reported no side effects from the vaccination. The last study visit for all subjects is projected to occur in June 2005.

An unexpected local injection site reaction for this DNA vaccine has been observed. Mild cutaneous lesions (0.5-1.0 cm diameter) at the vaccination site occurred after 4 of 44 (9%) vaccinations administered; these occurred in 3 of 15 (20%) subjects. Subjects were routinely asked to call if they experience any unusual problem after study vaccinations. The vaccination site cutaneous lesions did not alarm subjects enough to prompt them to contact the VRC Clinic prior to their next regularly-scheduled visit. In retrospect, three subjects reported that they experienced skin lesions that started as a small papule or vesicle within 3 days after vaccination. After a few days the papule or vesicle unroofed and a scab formed. There was surrounding mild erythema and mild induration. After the scab came off, the skin healed without treatment. None of the cutaneous lesions were associated with pustular exudates, fever, rash or urticaria. They did not appear to be either a local infection or an allergic reaction.

The first three cutaneous lesions were discovered at the first post-vaccination clinic visit (days 14 ± 3 Day); at that time they were largely resolved. The fourth cutaneous lesion was examined in the clinic while still in an active stage and it was biopsied at post-vaccination day 6. This biopsy demonstrated a microscopic subcutaneous and dermal perivascular lymphocytic infiltrate. The infiltrate was composed almost exclusively of CD3 positive cells, including both $CD4^+$ and $CD8^+$. There were rare eosinophils present and rare giant cells noted. The process appeared to be primarily a subcutaneous and dermal response to vaccination with cutaneous manifestations.

The reason these reactions have been seen in VRC 007 and not studies evaluating other DNA vaccines delivered intramuscularly by Biojector is not known. Whether these reactions correlate with the strength of the vaccine-induced immune response is also not yet known. Eight of the 14 subjects who remained in follow-up have had a vaccine-induced positive HIV ELISA by a commercial test at one or more timepoints; this includes all three subjects who had a cutaneous lesion. Preliminary immunogenicity data from VRC 007 (6-plasmid DNA) suggests the Env-specific T cell responses are similar to those seen in VRC 004 (4-plasmid DNA), and now the Gag- and Nef-specific responses are also present.

A rollover protocol, VRC 010, will offer willing and eligible VRC 007 subjects the option of receiving an adenoviral vector boost at the 10^{10} PU (particle units) dosage, as part of the initial evaluation of safety and immunogenicity of a DNA vaccine prime-adenoviral vector boost vaccine regimen for prevention of HIV infection. Protocol VRC 008 will provide better understanding of the occurrence, development and etiology of the vaccine-associated skin lesions. VRC 008 includes a plan to prospectively obtain baseline and post-vaccination photos of vaccination sites and when subjects consent, to also obtain skin biopsies of skin lesions. The optional skin biopsies will document whether the histology is consistent with the initial impression that the skin lesions are primarily an immune process or whether there is evidence of allergic, infectious or foreign body local reaction.

1.4 PREVIOUS HUMAN EXPERIENCE WITH VRC-HIVADV014-00-VP

The recombinant adenoviral vector product VRC-HIVADV014-00-VP (rAd) is a replication-deficient, combination vaccine containing four recombinant serotype 5 adenoviral vectors. These vectors contain gene sequences that code for clade B HIV-1 Gag and Pol as well as clade A,

clade B, and clade C Env protein. *In vivo* expression by these vectors produces immunogens that induce an immune response against HIV. The envelope genes were chosen as representative primary isolates from each of the three clades.

The Vaccine Research Center, NIAID, NIH in collaboration with the Division of AIDS (DAIDS), NIAID, NIH has sponsored three other intramural clinical trials (VRC 006, VRC 009 and VRC 010) and two extramural clinical trials (HVTN 054 and HVTN 057) in which this candidate vaccine is administered as either a single dose vaccine or as a booster vaccination to a DNA vaccine. All clinical follow-up visits for VRC 006 (04-I-0172) are complete, HVTN 057 has completed all study injections and follow-up visits are ongoing, while VRC 009 (05-I-0081), VRC 010 (05-I-0140) and HVTN 054 are open to accrual.

VRC 006: The Vaccine Research Center (VRC) is conducting VRC 006 (04-I-0172), "A Phase I Clinical Trial to Evaluate the Safety and Immunogenicity of a Recombinant Multiclade HIV-1 Adenoviral Vector Vaccine, VRC-HIVADV014-00-VP, in Uninfected Adult Volunteers." This is a randomized, placebo-controlled, double-blinded, dose escalation study to examine safety, tolerability and immune response following a single injection of VRC-HIVADV014-00-VP at a dose of 10^9 PU, 10^{10} PU, or 10^{11} PU. Each group includes 12 subjects (10 vaccine; 2 placebo). VRC 006 was initiated on July 19, 2004, the study completed enrollment of 36 subjects on November 10, 2004 and the last study visit occurred on April 27, 2005. The NIAID Intramural Data and Safety Monitoring Board (DSMB) reviewed the preliminary safety data through 14 days of follow-up prior to each dose escalation. The blinded data indicate that the vaccine appears to be safe for healthy subjects at the three dose levels evaluated. The 10^9 and 10^{10} PU dose levels are associated with less reactogenicity than the 10^{11} PU dose level. In both the 10^9 and 10^{10} PU dose groups the local and systemic parameters recorded on the 5-day diary card were none to mild in severity and none of the subjects experienced fever. In the 10^{11} PU dose group, four subjects reported fever on Day 1 (3 mild and 1 moderate in severity). Each of the four subjects with fever also reported moderate headache on Day 1 and three of these subjects also reported at least one other moderate systemic parameter (malaise, myalgia, chills). Two subjects without fever reported at least one moderate systemic symptom (malaise, myalgia, nausea). One subject in the 10^{11} PU dose group reported moderate injection site pain; injection site reactogenicity was otherwise none or mild.

As of April 27, 2005, there has been one grade 4 (potentially life-threatening) and three grade 2 (moderate) adverse events that are possibly related to vaccination. The study remains blinded to vaccine vs. placebo injection assignments. The grade 4 adverse event was a seizure that occurred 64 days after study injection in a healthy subject in the 10^{11} PU dose group who had a history of a single seizure three years prior to study enrollment. Following the review of past medical records and test results and given the history of a prior seizure and timing of event more than 2 months after study injection, the seizure was assessed as unrelated to study agent. The grade 2 adverse events possibly related to study agent include: 1) asymptomatic neutropenia noted 21 days after study injection in a subject known to sometimes have asymptomatic low neutrophil counts prior to enrollment; 2) diarrhea (duration one day) in a different subject on the third day after study injection and 3) steatohepatitis (fatty liver) diagnosed after extensive evaluation to identify the cause of a persistent grade 1 ALT (alanine aminotransferase) elevation that was noted starting 25 days after the study vaccination in a clinically asymptomatic subject. A hepatology consultant reported an impression that the condition likely existed prior to study enrollment. Contributing factors to the persistent grade 1 ALT may be alcohol consumption and

recent weight gain. A diagnosis of steatohepatitis is overall considered to be a grade 2 condition, but the liver function tests remained at grade 1 severity for about 5 months and then was normal range at last study visit; a repeat ultrasound showed fatty liver was still present.

The VRC 008 study proposes to use both the 10^{10} PU and the 10^{11} PU dose for the booster vaccinations. Although more reactogenicity has been observed with the 10^{11} PU dose, it appears to be a well-tolerated dose and analgesic/antipyretic nonprescription medications may be self administered for relief of the short-term symptoms. A protocol-specified interim immunogenicity analysis is in progress to compare the placebo and three dosage groups. The blinded immunogenicity data suggest a dose effect with increasing immune response at higher doses. The number of subjects with vaccine-induced ELISA at study week 12 by commercial HIV-antibody assay increased from 3 in the 10^9 PU group, to 6 in the 10^{10} PU group, and to 9 in the 10^{11} PU group among the 12 subjects (two placebo and ten vaccine recipients) per group. Preliminary immunogenicity data from VRC 006 suggests the majority of vaccinees develop both $CD4^+$ and $CD8^+$ Env-, Gag- and Pol-specific T cell responses. VRC 008 will be one of the first two studies of this prime-boost regimen and at this early stage of development for the rAd booster vaccine it is important to continue to evaluate different dosages to continue to gather data on dose-related immunogenicity and reactogenicity.

HVTN 054: This is the second Phase I study of the rAd vaccine as single agent in uninfected adult subjects. This blinded, dose escalation study was submitted to BB-IND 11661 in December 2004 and opened to accrual in April 2005. It is designed to enroll two groups of 24 subjects with low Ad5Ab titer (<1:12), who will be randomized to rAd or placebo in a 5:1 ratio. The first group of vaccinees will receive 10^{10} PU rAd and the second group will receive 10^{11} PU rAd.

HVTN 057: HVTN 057 (BB-IND 11894) is the first Phase I study to administer the adenoviral vector vaccine, VRC-HIVADV014-00-VP, as a booster vaccination. In this blinded Phase I study a single boost at 10^{10} PU (or a placebo) is administered to 70 participants (60 active; 10 placebo) who completed the injection regimen with VRC-HIVDNA009-00-VP (or placebo) in HVTN 052. The rAd boost is given at an interval of 6-9 months after the subject's first injection in HVTN 052. The first participant was enrolled on November 22, 2004 and on April 20, 2005 the last enrollment and study injection was completed. As of May 13, 2005 the still blinded reactogenicity results indicated local pain and/or tenderness reported by 55 (78.6%) participants, with maximal severity moderate, reported for 3 (4.2%) participants. Erythema and/or induration no greater than 25 cm^2 was reported for 10 (14.3%) participants, of whom 7 (10.0%) reported the erythema and/or induration as <10 cm^2. Systemic reactogenicity did not exceed moderate: 18 (25.7%) reported mild symptoms and 6 (8.5%) reported moderate symptoms. The most commonly reported mild symptoms were malaise and/or fatigue in 20 (28.5%) participants, myalgia in 15 (21.4%) participants, and headache in 10 (14.2%) participants. The most commonly reported moderate symptoms were headache in 7 (10.0%) participants, malaise and/or fatigue in 6 (8.5%) participants, and myalgia in 5 (7.5%) participants. Six (8.5%) participants reported grade 1 fever, none of which exceeded 38.5 ^{o}C.

VRC 009: VRC 009 is the second Phase I study of rAd as a booster vaccine. It is an open label study designed to enroll subjects who completed three vaccinations with 4 mg or 8 mg of VRC-HIVDNA009-00-VP in VRC 004 (03-I-0022) to receive a 10^{10} PU rAd booster vaccination. The first enrollment into this study occurred January 28, 2005 and as of May 5, 2005 eight subjects

were enrolled; four from the 4 mg DNA group and four from the 8 mg DNA group in VRC 004. The mean boost interval to date is 91 weeks [range 79-104 weeks] from first DNA prime vaccination. There have been no serious adverse events. All 8 subjects had mild pain at the injection site and 5 of 8 subjects had at least one mild or moderate symptom (malaise, myalgia, headache or chills). Seven of the 8 subjects have reached the first HIV ELISA testing timepoint and all have shown a vaccine-induced antibody by the commercial ELISA test method.

VRC 008 and VRC 010 (for VRC 007 rollover subjects) together will provide the first safety and immunogenicity data for the prime-boost regimen using the 6-plasmid DNA vaccine, VRC-HIVDNA016-00-VP for the priming vaccinations and VRC-HIVADV014-00-VP for the booster vaccination. The first booster injection of a subject primed with the 6-plasmid DNA vaccine occurred on May 4, 2005 when the first VRC 007 rollover subject was enrolled into VRC 010.

1.5 Rationale for Evaluating Devices for IM Injection of DNA Vaccine

The Vaccine Research Center, NIAID and Division of AIDS, NIAID have evaluated the safety and immunogenicity of three investigational DNA plasmid vaccines for HIV-1 and one investigational DNA plasmid Ebola vaccine. The HIV vaccine, VRC-HIVDNA009-00-VP, is now being evaluated in extramural trials. To date the DNA vaccines have been administered by a needleless delivery device (Biojector 2000), which is manufactured by Bioject Corporation (Portland, OR). The needleless design of the Biojector offers a safety advantage in eliminating the potential for accidental needle sticks and illicit reuse of disposable needles and syringes. The Biojector has the disadvantage of being more expensive to use than standard needle and syringe and requiring special training to use properly. A preclinical study in non-human primates (described in Section 2.4) indicates that the immunogenicity and systemic safety of VRC-HIVDNA016-00-VP are similar whether administered by either needle and syringe or by Biojector. The published literature, reviewed below, suggests that the Biojector is associated with more injection site reactogenicity than a standard needle and syringe for intramuscular injections. In the VRC's experience with the Biojector, occasional small 1-2 mm cutaneous lacerations, sometimes associated with subsequent ecchymoses, have been noted that are not seen with needle and syringe injections. In the context of future international Phase II and Phase III studies it is important to evaluate whether the DNA injections should be administered by needle and syringe. If equivalent from an immunological standpoint, this method of administration may reduce cost and ensure consistent intramuscular administration at numerous clinical sites. Recently there was the unexpected observation of mild skin lesions following 9% of the DNA vaccine injections in VRC 007 (04-I-0254). For planning future studies, it may also be helpful to obtain preliminary information through VRC 008 about whether any skin lesions are observed following IM injection of VRC-HIVDNA016-00-VP by needle and syringe.

Needle and syringe injections have been the principal method of vaccine administration worldwide as they are economical and suitable for use in various environments [11]. However, in developing countries there are community risk concerns associated with conventional needle and syringe use. Improper injection practices, an acknowledged problem in developing countries, can result in the use of nonsterile needles or syringes with transmission of blood-borne pathogens across a patient population. In a 1999 literature review, it was reported that more than 50% of all injections administered in 14 of 19 developing countries were unsafe [12]. In all clinical settings, there is a risk of needle stick injuries for health care workers and the burden of proper needle disposal. However, the risk of a needle and syringe as an injection device for an individual

patient is minimal when a sterile disposable needle and syringe are used and disposed of properly. A monitored study site participating in a clinical trial can be supplied with the resources needed to ensure proper use and disposal of needles and syringes.

Needle-free injection systems have been used as an alternative to needle and syringe injections of vaccines. Needle-free devices eject pressurized fluid through a small orifice in the nozzle, resulting in percutaneous delivery of the liquid vaccine with intramuscular, subcutaneous, or intradermal deposition [13]. Foremost among the advantages of pressurized injection systems as a method of vaccine administration is the inherent increase in safety afforded by a needle-free device, both to vaccinees (by ensuring that a nonsterile device is not used) and to healthcare workers (by eliminating needle-stick injuries). Some needle-free injection systems have been a valuable public health instrument in developing countries for mass vaccination campaigns, and may be important globally in the event of a bioterrorist attack [13].

Animal and human studies, involving different vaccines and routes of administration, have been conducted to evaluate the safety and immunogenicity of needle-free devices in comparison to standard needle and syringe dispensation. Regardless of the vaccine injected, a consistent finding in several studies has been an increase in local inflammation in those individuals vaccinated via needle-free injection devices [11, 14-17]. The increase in local reactogenicity may reflect the greater distribution of injectate and the minor tissue injury associated with these systems [15, 16]. The Department of Safety Assessment, Merck Research Laboratories published a study that included comparison of needle and syringe to Biojector for intramuscular (IM) administration of DNA plasmid vaccines to guinea pigs. Six weeks after IM injection by either method, the vast majority of the DNA plasmid was in the muscle (hamstrings) and skin near the injection site, with skin showing higher copy counts than muscle. Low levels of DNA plasmids were also detectable in draining lymph nodes. In the early timepoints (1-7 days) low systemic exposure could also be detected. Biojector delivery, as compared to needle injection, increased the uptake of DNA plasmids in both muscle and the skin near the injection site, as well being associated with slightly more dispersion to distal sites. Neither method was associated with integration of DNA plasmid into host cellular DNA [18].

The wider dispersion pattern of the injectate observed with needle-free injection devices, such as Biojector, and the resultant local inflammatory response may also enhance immunogenicity through increased antigen presentation and recruitment of immune-competent inflammatory cells [16, 19]. For example, it was reported that in rabbits immunized with Biojector, significantly improved antibody response to a *Pf*CSP DNA malaria vaccine as compared to needle and syringe administration [20]. Some human studies have also reported increased antibody response following use of the Biojector device. In a study evaluating trivalent influenza vaccine administered by needle and syringe or by needle-free jet injectors (either VitaJet subcutaneously or Biojector intramuscularly), the Biojector group had higher post-vaccination antibody titers to one of the three influenza antigens [11]. In another study, subjects vaccinated with HAVRIX by Biojector had a significant increase in anti-hepatitis A virus antibody geometric mean titers and seroconversion rates when compared with those vaccinated by needle and syringe [15].

In the first human study (N=20) of the *Pf*CSP DNA malaria vaccine four groups of 5 study subjects were sequentially enrolled to receive three injections intramuscularly by needle and

syringe at 4 dose levels that ranged between 20 μg and 2.5 mg per injection. The vaccine was safe and well tolerated when delivered by needle and syringe. A majority of subjects developed antigen-specific CTL responses with more responses in the higher dose groups, but none of the subjects had detectable antibodies to the *Pf*CSP. On the basis of preclinical evaluation of safety and immunogenicity when different injection devices were used for administration, different injection devices were evaluated in a subsequent human clinical trial (N=21). The three injection methods were: needle IM, Biojector IM, and Biojector with 70% of the dose administered IM and 30% administered intradermally (ID). Each of the three groups included 5 vaccinees (2.5 mg dose) and 2 control subjects. The schedule included injections of 1 mL total volume at weeks 0, 4 and 8. The IM/ID administration plan required 4 injections at each time point. Adverse events were generally mild and limited to the injection site with Biojector injections associated with about twice as many adverse events per injection. However, study subjects preferred the Biojector injections [16]. Regardless of the method of administration, anti-*Pf*CSP-specific antibodies could not be detected in the human clinical trial samples, although antigen-specific immune responses as assessed by IFN-γ enzyme-linked immunospot (ELISPOT) were detected in all vaccinees [16, 21]. Experience with this DNA vaccine indicates that intramuscular injection by needle and syringe does not result in different systemic safety concerns when a needleless injection device is used to deliver it intramuscularly.

1.6 CONSIDERATIONS RELATED TO PRE-EXISTING ANTIBODY TITER TO ADENOVIRUS SEROTYPE 5

An important concern in the development of adenoviral vector vaccine is the effect of pre-existing neutralizing antibodies to adenoviruses in the adult population on the response to an adenoviral vector vaccine. The utility of adenoviral vector vaccines in humans may be limited when a high titer of neutralizing Ad antibody is present at the time of vaccination [22]. The adenoviral vector in this study is a replication defective adenovirus of serotype 5. Over fifty distinct human adenovirus serotypes have been defined worldwide based on type specific neutralizing antibody assays. These cause a variety of common infections including respiratory infections [23]. Seroepidemiological surveys to assess adenovirus in various U.S. and European populations suggest that adenovirus serotype (Ad)1, Ad2, and Ad5 are most common and present in over half of children tested [22, 24, 25]. Adenovirus serotype 5 antibody was detected by ELISA in one study of young adults (median age of 27), 55% of which had neutralizing titers in excess of 1:20 [26]. Data on other non-U.S. populations have suggested that similar or higher rates of seroprevalence will be found in populations targeted for vaccination especially in the developing world [27].

Studies in humans and animals suggest that pre-existing immunity to adenovirus serotype 5 may attenuate the immune responses to Ad5-based adenoviral vectors [28]. The use of higher titer vaccine and/or prime-boost strategies may overcome this potential difficulty [29].

The effect of pre-existing Ad5 immunity on the safety and tolerability of Ad5 vector vaccines is an important concern for which more data are needed. The MRKAd5 vaccine experience was reviewed at the AIDS Vaccine 2004 Conference (Lausanne, Switzerland) [30]. It was noted that more than 400 subjects have received one of the investigational MRKAd5 HIV vaccines. Fever was more common in subjects with low (≤1:200) Ad5 Ab titer and was more common at the 1 x 10^{11} viral particle (VP) dose, but the Ad5-based vaccines were generally well tolerated. Preexisting anti-Ad5 neutralizing antibody was also noted to “dampen” the immunogenicity of

the Ad5 vector vaccine but this could be overcome at higher dose levels. Therefore, it is important to evaluate safety and immunogenicity of rAd5 vector vaccines in the setting of both low and high pre-existing Ad5Ab titers using different dosages of the vaccine.

The VRC has evaluated a set of samples from subjects who were screened to enroll in investigational vaccine studies at the VRC Clinic. In these samples, 52 of 85 (61%) subjects had Ad5Ab titers ≤1:500. In a comparison of 9 samples evaluated by both a 50% neutralization assay used by Merck [31] and a 90% neutralization assay that will be used by VRC in this study [32], the VRC titers were about 3-fold higher than the titers measured by the Merck assay. On the basis of this preliminary population data and comparison with what is known about the MRKAd5 vaccine experience, the definition of a "low antibody" titer in this study has been set at ≤1:500 in the 90% neutralization assay used by VRC.

VRC 008 is designed to enroll approximately equal numbers of subjects with high and low pre-existing Ad5 antibody in order to gain preliminary information in healthy adults on whether the safety and immunogenicity response to the VRC adenoviral vector vaccine is different in these two groups of subjects. Much larger studies will be needed to definitively answer these questions; however, this exploratory evaluation can help in selecting sample size and booster dosage to use in future Phase II and Phase III studies of the adenoviral vector vaccine.

1.7 MEASURES OF IMMUNOGENICITY

This Phase I study will provide a preliminary assessment of the immunogenicity of VRC-HIVDNA016-00-VP and VRC-HIVADV014-00-VP in a prime-boost regimen by employing intracellular cytokine staining (ICS) and ELISPOT assays that evaluates CTL responses, as well as assays that evaluate HIV-specific antibody responses. The ICS assay is based upon previously published methods [33] and quantitates the frequency of $CD4^+$ and $CD8^+$ cells that produce interleukin-2 or interferon-gamma, in response to pools of overlapping peptides representing HIV antigens (Gag, Pol, Nef or Env) from specific HIV clades. These clade-specific peptides will also be used to detect T-cell responsiveness by an ELISPOT assay modified from a previously published method [34]. Antibody responses to HIV-specific antibodies will be evaluated using an enzyme-linked immunosorbent assay (ELISA) [35]. The ability of the vaccine to elicit neutralizing antibody against HIV-1 strains from clades A, B, and C will be evaluated by a flow cytometric assay that measures the capacity of sera to block single round infection of individual PBMC [36]. The pre-existing and post-vaccination presence of adenovirus serotype 5 neutralizing antibody in study volunteers will be evaluated from frozen serum samples using a previously published luciferase transgene detection method [32]. Other assays may also be completed from stored samples at a later date if further elucidation of immunogenicity is of interest.

2. BACKGROUND ON VACCINE

2.1 HIV-1 DNA VACCINE PLASMIDS IN VRC-HIVDNA016-00-VP

VRC-HIVDNA016-00-VP, a six-component multiclade plasmid DNA vaccine, expressing Gag, Pol and Nef proteins from clade B HIV-1 and Env glycoproteins from clades A, B and C, is intended for use as a preventive HIV-1 vaccine. The vaccine has been designed to elicit immune responses against several proteins from a variety of HIV-1 strains. The manufacturer of each

plasmid DNA drug substance for preclinical safety studies and clinical trial material, from the establishment of the master cell bank (MCB) through final product, is Vical Incorporated (San Diego, CA). Non-GMP material for preclinical immunological studies was manufactured by Althea Technologies, Inc. (San Diego, CA) by a similar process.

The drug substances for VRC-HIVDNA016-00-VP are six closed circular plasmid DNA macromolecules, VRC 4401, VRC 4409, VRC 4404, VRC 5736, VRC 5737 and VRC 5738 combined in equal concentrations (mg/mL). VRC 4401 encodes for the clade B HIV-1 Gag structural core protein that encapsidates the viral RNA and exhibits highly conserved domains. VRC 4409 encodes for clade B polymerase (Pol), which is also highly conserved, and VRC 4404 encodes for clade B Nef, an accessory protein against which a vigorous T-cell response is mounted in natural infection. The DNA plasmid expressing HIV-1 Pol has been modified to reduce potential toxicity through the incorporation of changes in the regions affecting the protease, reverse transcriptase, and integrase activities. Two amino acids in the myristoylation site in the HIV-1 *nef* gene were deleted to abrogate MHC class I and CD4+ down-regulation by the Nef protein [37, 38]. No modifications were made to the amino acid sequence of Gag. The other three plasmids express synthetic versions of modified, truncated envelope glycoproteins (gp145) from three strains of HIV-1: VRC 5736 (clade A), VRC 5737 (clade B) and VRC 5738 (clade C). The sequences used to create the DNA plasmids encoding Env are derived from three HIV-1 CCR5-tropic strains of virus. These genes have been modified to improve immunogenicity, which has been demonstrated in mice [39] and monkeys. The vaccine will potentially elicit immune responses to a broad range of HIV-1 strains.

Plasmids containing Gag, Pol, Nef and Env complementary DNA (cDNA) were used to subclone the relevant inserts into plasmid DNA expression vectors that use the CMV/R promoter and the bovine growth hormone polyadenylation sequence. All the plasmids expressing the HIV-1 genes were made synthetically with sequences designed to disrupt viral RNA structures that limit protein expression by using codons typically found in humans, thereby increasing gene expression. The translational enhancer region of the CMV immediate early region 1 enhancer was substituted with the 5′-untranslated HTLV-1 R-U5 region of the human T-cell leukemia virus type 1 (HTLV-1) long terminal repeat (LTR) to optimize gene expression further. The DNA expression vectors are similar to those used for other candidate vaccines currently undergoing evaluation in clinical studies by the VRC and DAIDS/NIAID/NIH.

The DNA plasmids have been produced in bacterial cell cultures containing a kanamycin selection medium. In all cases, bacterial cell growth is dependent upon the cellular expression of the kanamycin resistance protein encoded by a portion of the plasmid DNA. Following growth of bacterial cells harboring the plasmid, the plasmid DNA is purified from cellular components. The Gag plasmid (VRC 4401) is 5886 nucleotide pairs in length and has an approximate molecular weight of 3.9 MDa; the Pol plasmid (VRC 4409) is 7344 nucleotide pairs in length and has an approximate molecular weight of 4.8 MDa; the Nef plasmid (VRC 4404) is 5039 nucleotide pairs in length and has an approximate molecular weight of 3.3 MDa; the clades A, B, and C Env plasmids (VRC 5736, 5737, and 5738) are 6305, 6338 and 6298 nucleotides in length, respectively, and have an approximate molecular weight of 4.2 MDa.

The plasmid and host *E. coli* strain used in the production of the vaccine are characterized in accordance with the relevant sections of the "Points to Consider in the Production and Testing of New Drugs and Biologicals Produced by Recombinant DNA Technology" (1985), the "Supplement: Nucleic Acid Characterization and Genetic Stability" (1992), "Points to Consider

in Human Somatic Cell Therapy and Gene Therapy" (1991, 1998), and "Points to Consider on Plasmid DNA Vaccines for Preventive Infectious Disease Indications" (1996).

2.2 ADENOVIRAL VECTORS IN VRC-HIVADV014-00-VP

VRC-HIVADV014-00-VP is a replication-deficient, combination vaccine containing four recombinant adenoviral vectors. These vectors contain gene sequences that code for clade B HIV-1 Gag and Pol as well as clade A, clade B, and clade C Env proteins. *In vitro* expression by these vectors produces immunogens that induce an immune response against HIV. The envelope genes were chosen as representative primary isolates from each of the three clades.

The process for constructing the four VRC-HIVADV014-00-VP recombinant adenoviral vectors is based upon a rapid vector construction system (AdFAST™, GenVec, Inc.) used to generate adenoviral vectors that express the four HIV antigens gp140(A), gp140(B)dv12, gp140(C) and GagPol(B) driven by the cytomegalovirus (CMV) immediate-early promoter. Manufacturing is based upon production in a proprietary cell line (293-ORF6), yielding adenoviral vectors that are replication deficient. The vectors are purified using CsCl centrifugation. The product is formulated as a sterile liquid injectable dosage form for intramuscular injection.

The GV11 adenoviral backbone was chosen to reduce the risk of replication-competent adenovirus (RCA) generation during clinical production. The GV11 backbone contains deletions of two essential regions, E1 and E4, as well as a partial E3 deletion that render the vaccine product replication-deficient. The generation of RCA would require two independent recombination events in a single adenovirus genome, predicted to be an extremely rare event [40].

The Ad_{GV} (HIV).11D vectors contain HIV-1 antigen open reading frame (ORF) expression cassettes inserted to replace the deleted adenovirus E1 gene region. Other deleted adenovirus regions include a partial E3 and all of E4, which has been replaced with a transcriptionally inert spacer element (T1S1) that enhances production of the adenoviral vectors [41].

The 293-ORF6 cell line used to propagate these E1, E4 and partial E3 deleted vectors was developed at GenVec, Inc. These cells were constructed by stably transforming 293 cells (which are of human embryonic kidney origin) with an inducible E4-ORF6 expression cassette. This enables the cells to efficiently complement the E1-, E4-, and partial E3-deleted adenoviral vectors, provide increased transgene capacity and greatly reduce the potential to generate replication-competent adenovirus. The particular clone that has given rise to the cell line is the A232 clone. All references to the 293-ORF6 cell line refer to cells derived from the original A232 clone. This replication-deficient adenoviral vector system has been used to produce TNFerade, a TNF-alpha gene-based product [42]. An assay for replication-competent adenovirus is performed in the final release testing for all vectors; RCA has not been observed in this packaging system during the manufacture of multiple gene-based products.

The four vaccine adenoviral vectors are generated by introducing a DNA plasmid consisting of the adenoviral genome into the 293-ORF6 cells. The adenoviral vector in the lysate from the transfected cells is serially passaged to expand the titer of adenoviral vector. The identity and integrity of the passages is verified by polymerase chain reaction (PCR) and expression of the HIV-1 gene is confirmed by Western Blot analysis. Purified adenoviral vector is produced by infecting the 293-ORF6 cells with the adenoviral vector in the lysate; after the infection of the cells is complete, the material is collected and the vector is purified from the cells. The four

vaccine adenoviral vectors are purified using a cesium chloride (CsCl) gradient centrifugation process. CsCl is removed by dialyzing the virus preparation against the final formulation buffer (VRC-DILUENT013-DIL-VP). Purified adenoviral vector serves as a vector bank for subsequent production of the four vaccine adenoviral vectors. This vector bank is tested for sterility, mycoplasma and other adventitious agents prior to its being used for manufacturing of clinical supplies.

2.3 Preparation of the Bulk Plasmid and Final Product

2.3.1 VRC-HIVDNA016-00-VP

Bulk plasmid DNA is manufactured at Vical Incorporated (San Diego, CA). One source of each plasmid (supplied by VRC) is used to prepare a Master Cell Bank (MCB) for each plasmid. Vical Incorporated formulates the bulk DNA for each of the components at 4 mg/mL in phosphate buffered saline (PBS). The six plasmids are mixed to form the final bulk vaccine product. Sterile filtration and fill operations are conducted under aseptic conditions in a Class 100 environment. Vical Incorporated fills and performs release testing for the VRC-HIVDNA016-00-VP with the exception of expression, which is tested by the VRC or a subcontractor.

Clinical trial material is tested as bulk plasmid DNA and final product. VRC, or a subcontractor, conducts the gene expression testing on the filled VRC-HIVDNA016-00-VP product and bulk VRC 4401, VRC 4409, VRC 4404, VRC 5736, VRC 5737 and VRC 5738. Upon confirming gene expression, VRC releases the product. Final product meeting all test specifications is released for use in the proposed clinical study.

2.3.2 VRC-HIVADV014-00-VP

The investigational vaccine, VRC-HIVADV014-00-VP, is manufactured by Genvec, Inc. (Gaithersburg, MD) at a contract manufacturer, Molecular Medicine (San Diego, CA). DNA plasmids produced by the Vaccine Research Center, NIAID, NIH (Bethesda, MD) are used to construct the adenoviral vector clinical seed stock. The Phase I clinical production for each adenoviral vector is performed by Molecular Medicine from clinical seed stock produced by Bioreliance (Rockville, MD).

The multiclade adenoviral vector vaccine product, VRC-HIVADV014-00-VP, is a 3:1:1:1 ratio of the adenoviral vectors that encode for HIV-1 Gag/Pol polyprotein from clade B and HIV-1 Env glycoproteins from clades A, B, and C, respectively. Final product meeting all test specifications will be released for use in the proposed clinical study. Vials are filled to 1.2 mL volume with 1×10^{10} PU/mL or 1×10^{11} PU/mL.

The final formulation buffer (FFB) is custom manufactured by BioWhittaker (Frederick, MD). The FFB is composed of sodium chloride, Tris buffer, trehalose•$2H_2O$ (low endotoxin), magnesium chloride•$6H_2O$, monooleate (Tween 80) and water for injection (WFI).

2.4 Preclinical Studies

Preclinical studies to evaluate safety and immunogenicity of VRC-HIVDNA016-00-VP and similar DNA plasmid vaccines alone or followed by the rAd vaccine are summarized in this section. More detailed summaries of preclinical studies done with each vaccine are available in the Investigator's Brochure for each study vaccine.

2.4.1 Preclinical Biodistribution Studies in Rabbits

GLP biodistribution studies of several VRC DNA plasmid vaccines administered IM by Biojector to rabbits have been completed. These include VRC-4302 single plasmid vaccine, VRC-HIVDNA006-00-VP 6-plasmid vaccine, VRC-EBODNA012-00-VP 3-plasmid vaccine, and VRC-SRSDNA015-00-VP single plasmid. A consistent finding by PCR evaluation from the timepoints evaluated [these ranged from Study Day (SD) 8 through SD 61] is that the vector is primarily localized to the subcutis at the injection site. The magnitude of positive signal observed in tissues from the early timepoint was greatly diminished at the later time points, indicating eventual clearance of the test article.

A GLP biodistribution study was completed with the adenoviral vector vaccine, VRC-HIVADV014-00-VP, administered IM by needle and syringe in rabbits. The distribution profile consisted of the test article present at the injection site subcutis (5/10 animals, SD 9; 2/10 animals, SD 61), muscle (4/10 animals, SD 9), spleen (10/10 animals, SD 9; 6/10 animals, SD 61; 5/10 animals, SD 91) and liver (9/10 animals, SD 9; 2/10 animals, SD 61), with a sporadic finding in bone marrow (1/10 animals, SD 9). The number of copies present in positive tissues and the number of animals with positive tissues decreased considerably between SD 9 and SD 61 and continued to decrease between SD 61 and SD 91.

2.4.2 Preclinical Toxicology Studies of Prime-Boost Regimens in Rabbits

The objective of the study, which was conducted under GLP by Gene Logic, Inc. (Gaithersburg, MD), was to assess the potential toxicity of VRC-HIVADV014-00-VP when administered alone or as a boost for New Zealand White rabbits vaccinated with the 4-plasmid DNA vaccine, VRC-HIVDNA009-00-VP.

For the VRC-HIVDNA009-00-VP DNA prime/VRC-HIVADV014-00-VP rAd boost study, ten animals/gender were dosed via intramuscular injection. DNA vaccine (4 mg) and PBS control were administered four times (SD 1, 22, 43, and 64). Dosage was divided into 2 intramuscular injections spaced approximately 1 inch apart (0.5 mL/injection site; dose volume for each injection was not adjusted for body weight) for each time point. Injections were administered into the thigh muscle using a Biojector 2000 needle-free injection management system (Biojector 2000) on alternate sides for each time point. Adenoviral vector vaccine (VRC-HIVADV014-00-VP) at 10^{11} PU (1 mL volume) or the diluent control (VRC-DILUENT013-DIL-VP) injections (1 mL volume) were divided into two 0.5 mL injections for administration with a needle and syringe into the hind thigh muscle approximately 1 inch apart per day of dosing (SD 85 and 106). Injections were administered on alternate sides for each time point. A 1 mL volume was administered regardless of body weight for the DNA and adenoviral vector vaccines and their respective controls. One half of the animals (5/gender) were sacrificed on study day 108 and the remainder on study day 120. Injections were administered at a shaved/marked site. The sites were re-shaved and re-marked as needed in order to visualize the injection site.

Parameters evaluated included mortality, clinical signs of toxicity, Draize (local reactogenicity) observations, body weights, body weight changes, food consumption, ophthalmologic examinations, clinical pathology (chemistry, hematology and coagulation), body temperatures, gross pathology, organ weights, and histopathology. Observations were also made of motor function and behavior during the study.

All animals survived to sacrifice and necropsy. No prime-boost treatment-related observations

were made with regard to morbidity/clinical observations and ophthalmology. However, possible prime-boost treatment effects were seen with body weights and changes, particularly in treated females. Differences began to be noted as early as study day 36, but became statistically significantly different from control females on study days 71, 78, 92, 99, and 108 for body weights and days 85-92 for body weight changes in prime-boost treated females. These animals continued to gain weight over the course of the study, but did not gain as much weight as the controls.

In the prime-boost regimen, vaccination with the DNA prime administered by Biojector resulted in Draize observations of minimal to moderate edema and erythema increasing in frequency and severity with repeated dosing. These observations also occurred in the control animals but to a lower amount and lesser degree. These findings were consistent with previous toxicology studies performed with DNA vaccination alone. Boost (adenoviral vector delivered by needle and syringe) injections did not increase the frequency or severity (minimal erythema and/or edema in a few treated animals) of the Draize observations seen at earlier timepoints (after priming doses).

In prime-boost animals there were histopathological findings of inflammation in the perineural tissue surrounding the sciatic nerve (near the injection site). There were chronic inflammatory cells (small macrophages and lymphocytes) in the connective tissue around the sciatic nerve and in adjacent lymphatics and blood capillaries. This inflammation was likely the result of drainage of the distal injection sites toward proximal lymph nodes. The injection site reactions decreased in frequency and severity in the recovery sacrifice animals compared to the immediate sacrifice animals receiving the prime-boost regimens, demonstrating the reversibility of the injection site reactions.

In the prime-boost treated animals, fever was seen in treated males and females in the 24 hours subsequent to the initial treatment, but only in the first 3 hours after the second adenoviral vector boost (only in treated females). These fevers resolved by 48 hours after the initial treatment and 24 hours after the second (treated females only) adenoviral vector boost.

Food consumption was also decreased compared to controls in the 24 hours to 48 hours following each adenoviral vector vaccination, but resolved, and did not result in differences in body weights or changes in males or females inoculated with adenoviral vector alone or treated males in the prime-boost regimen. Significant differences in body weight changes in treated females were noted in treated females in the prime-boost regimen (although these differences began prior to exposure to adenoviral vector as discussed above).

There were additional observations, particularly in clinical chemistries and hematology parameters, which were unclear in their relationship to treatment. They either remained within the historical normal range for the species, even though there were statistically significant differences from matched control animals on study, or they were outside of the normal range and different from the control animals on study. They were <u>not</u> consistent between genders or across timepoints. None of these findings appeared to correlate with clinical observations or gross or histopathological findings. Of note was the finding of statistically significant (from matched controls on study) elevated triglycerides on the day subsequent to the initial adenoviral vector boost in treated males (mean was between 3-4 times the ULN), but not treated females, receiving the prime-boost regimen. There were no differences between groups in this parameter after the second adenoviral vector boost, in either gender. At day 86, several differences (statistical and

not within normal historical range) were noted in Group 4 hematological values, including lower platelets and neutrophils (females), lower lymphocytes (males) and higher neutrophils (males). Lack of corresponding data in the opposite sex and over multiple timepoints makes any correlation to the vaccine unclear.

2.4.3 Preclinical Safety and Immunogenicity of DNA Vaccination with Administration by Biojector or Needle and Syringe

A non-GLP non-human primate immunogenicity study comparing delivery via the Biojector 2000 to injection with needle and syringe of the HIV 6-plasmid DNA vaccine (VRC-HIVDNA016-00-VP) was conducted by Beth Israel Deaconess Medical Center, Harvard Medical School (Boston, MA) and the Vaccine Research Center (Bethesda, MD). A contract facility, BioQual, Inc. (Rockville, MD), maintained the primates and provided technical assistance with the study. The study title is: "*Evaluation of Delivery Methods of DNA Vaccination in the Non Human Primate Model*" and study authors are: Srinivas Rao, D.V.M., Ph.D., Norman Letvin, M.D., John Mascola, M.D.

This study was designed to compare the immunological response to an HIV vaccine when administered by the Biojector 2000 or a needle and syringe. Animals were also evaluated by a trained veterinary technician or an attending veterinarian at daily intervals. Detailed evaluation of the animal and the injection site was performed when the animals were anesthetized.

Well-characterized plasmid DNA (Althea Technologies, Inc., San Diego, CA) expressing HIV-1 clade B Gag, Pol, Nef proteins and clades A, B and C Env were used for the immunizations. The plasmids express the same proteins as those contained in the 6-plasmid vaccine, VRC-HIVDNA016-00-VP.

Groups of outbred Cynomolgus macaques (6 monkeys per group) were vaccinated with 4 mg of the DNA vaccine (1 mL injectate into quadriceps muscle) using either needle and syringe or the Biojector 2000 at weeks 0, 4 and 8.

Blood was collected for immunologic assays at two-week time intervals for the first 12 weeks, and at four-week intervals through week 24. IFN-γ ELISPOT assays, intracellular cytokine staining (ICS) assays (week 6) and ELISA assays (week 12) were performed to assess the magnitude of vaccine-elicited cellular and humoral immune responses.

The ELISPOT and ICS results were similar for monkeys vaccinated by Biojector and needle and syringe. T cell responses to clade B Gag, Pol, Nef, and Env were detected starting at 6 weeks in both groups. Low ELISA antibody titers were detected in both groups of animals. There were no significant differences between monkeys vaccinated by Biojector and those vaccinated by needle and syringe.

Animals did not show any obvious adverse clinical effect (e.g., favoring the injected limb, redness, abscess formation or induration) with either method of injection. Complete blood counts (performed monthly) demonstrated values generally within normal range for Cynomolgus macaques in the study laboratory.

Vaccination with the Biojector 2000 and needle/syringe did not demonstrate significant differences in the HIV-1 specific antibody and cellular responses in Cynomolgus macaques when the injectate was delivered by the IM route. The results of this study showed that vaccine delivered by needle and syringe elicited similar immunological responses to those delivered by the Biojector 2000. Needle and syringe and Biojector injections appeared to be similarly well tolerated by the study animals as indicated by veterinary observation and complete blood count data.

2.4.4 Immune Responses to VRC-HIVDNA009-00-VP Prime Followed by VRC-HIVADV014-00-VP Boost in Cynomolgus Macaques (VRC-02-035)

Non-GLP studies were conducted at Beth Israel Deaconess Medical Center, Harvard Medical School (Boston, MA) and the Vaccine Research Center (Bethesda, MD) to investigate the magnitude and breadth of cellular and humoral immune responses in Cynomolgus macaques that were elicited by different DNA prime/rAd boost immunization regimens using the DNA plasmid and rAd vaccines intended for use in human trials. GLP-grade DNA plasmid expressing clade B Gag-Pol-Nef fusion protein [produced by Althea Technologies, Inc. (San Diego CA)] and the multiclade A, B and C HIV-1 Env plasmids contained in VRC-HIVDNA009-00-VP (BB-IND 10681) were used for the prime immunization. GMP-grade VRC-HIVADV014-00-VP was used as the rAd boost. Groups of six outbred adult Cynomolgus macaque monkeys were immunized in this study. This summary describes results for the group that was immunized with 8 mg of the DNA vaccine delivered IM at weeks 0, 4, and 8 by Biojector, and 10^{11} PU of the rAd vaccine construct delivered IM by needle and syringe at week 38. In each case, plasmid vaccine was delivered as two 0.5 ml injections in the quadriceps muscles using a No. 3 Biojector syringe. Cynomolgus macaques receiving DNA prime/rAd boost immunizations elicited potent and broad cellular immune responses simultaneously to all viral antigens. In addition, they elicited potent antibody responses to these envelope antigens and Gag antigen as measured by ELISA. Monkeys that received the DNA plasmid vaccine prime and rAd boost generated responses to clades A, B and C Env peptide pools in all six animals following the DNA prime immunizations, as well as following the rAd boost.

Five of six animals developed antibody responses to all three envelope antigens. One animal developed antibody responses only to clades A and C envelope antigens. All six monkeys had strong Env antibody responses after adenovirus boost. These data demonstrate that the clinical rAd product is immunogenic, when given with a DNA prime, and induces cellular immune responses against clades A, B, and C Env, as well as Gag and Pol, and antibody responses against clades A, B, and C Env, as well as Gag. Adenoviral vector boosting increases the immune responses several fold.

2.5 RELEASE CRITERIA

Vical Incorporated will perform release testing for VRC-HIVDNA016-00-VP. The Vaccine Research Center (VRC) will test gene expression.

GenVec, Inc will perform release testing for VRC-HIVADV014-00-VP. The safety testing performed prior to release of the manufactured lots of adenoviral vectors includes, but is not limited to, the verification of sterility and absence of contaminating organisms by testing for

mycoplasma and endotoxin, determination of the level of replication-competent adenovirus (RCA), determination of host cell DNA and protein levels and genetic structural integrity.

Additional viral safety testing is performed on the Master Vector Banks and the final product. This includes, but is not limited to, tests for *in vivo* and *in vitro* inapparent adventitious virus, HIV-1, HIV-2, human T-cell lymphotropic virus (HTLV) I, HTLV II, cytomegalovirus (CMV), hepatitis B, hepatitis C, Epstein-Barr virus, adeno-associated virus (AAV), parvovirus B19, human herpes virus (HHV)-6, HHV-7, HHV- 8, and fluorescent product enhanced reverse transcriptase (F-PERT; a test for retroviruses). In addition to safety testing, the level of HIV-1 gene expression is also quantified from manufactured lots of the four adenoviral vectors by transfection and Western Blot analysis.

3. STUDY OBJECTIVES

3.1 PRIMARY OBJECTIVE

- To evaluate the safety and tolerability in humans of VRC-HIVDNA016-00-VP at a dose of 4 mg administered intramuscularly using two different methods, either needle and syringe or Biojector followed by a booster vaccine of VRC-HIVADV014-00-VP at a dose of 10^{10} PU or 10^{11} PU administered intramuscularly using a needle and syringe

3.2 SECONDARY OBJECTIVES

- To evaluate whether VRC-HIVDNA016-00-VP at a dose of 4 mg administered intramuscularly using two different methods, either needle and syringe or Biojector, shows a positive Gag, Pol or Nef response, as well as a positive Env response as assessed by HIV-specific peptide pool-stimulated T cell responses in the 4 weeks after the third dose of vaccine.
- To evaluate the immunogenicity of VRC-HIVADV014-00-VP by cellular responses (intracellular cytokine staining and ELISPOT) through 6 weeks after the booster vaccination when administered intramuscularly at 10^{10} PU or 10^{11} PU by needle and syringe to subjects with Ad5Ab titers >1:500 and ≤1:500 who previously received three injections of VRC-HIVDNA016-00-VP.
- To evaluate adenovirus serotype 5 neutralizing antibody titers at 4 weeks after the VRC-HIVADV014-00-VP booster injection.
- To monitor the social impact of participating in an HIV-1 vaccine clinical trial.

3.3 EXPLORATORY OBJECTIVES

- To evaluate the immunogenicity of the prime-boost regimen as indicated by intracellular cytokine staining, ELISPOT, vaccine antigen-specific ELISA, neutralization assays and other immunological assays at intervals beginning with the first VRC-HIVDNA016-00-VP injection through 18 weeks after the VRC-HIVADV014-00-VP booster injection.
- To evaluate the long-term immunogenicity of the prime-boost regimen in subjects who agree to have blood drawn at about Week 94.

4. STUDY DESIGN

This is a Phase I randomized study to examine safety and tolerability of and immune response to a schedule of 3 DNA plasmid vaccinations (administered by either needle and syringe or Biojector) followed by one adenoviral vector (rAd) booster vaccination (administered at either 10^{10} or 10^{11} PU dose). The hypotheses are that: 1) this regimen will be safe for human administration and elicit immune responses to HIV-1; 2) Biojector and Needle/Syringe are both safe to use for IM injection of the DNA vaccine and 3) subjects with both low and high pre-existing Ad5Ab titer will have a boost in immune response to HIV-1 peptides following the rAd booster vaccination. Forty subjects, half with high and half with low Ad5Ab titers will be randomized in a 1:1 ratio to receive DNA vaccinations by either needle and syringe (N/S) or by Biojector and also randomized in a 1:1 ratio to receive a booster vaccination with either 10^{10} PU or 10^{11} PU rAd vaccine. Safety of the vaccine regimen will be evaluated at scheduled study visits and by study subject report.

The study will provide safety data on the following number of subjects per vaccination type:

Biojector DNA vaccine primes: N = 20
Needle and Syringe DNA vaccine primes: N=20
10^{10} PU rAd booster vaccination: N=20
10^{11} PU rAd booster vaccination: N=20

Specimens to evaluate immunogenicity will be taken at baseline and at specified time points. The HIV-1 Gag-, Pol-, Nef-, and Env-specific immune responses will be assessed by cellular immune function assays and humoral immunity assays. The 40 study subjects will require 42 weeks on study to complete the prime-boost regimen and follow-up. The rAd booster vaccination dosage will be blinded until 6 weeks of safety and immunogenicity evaluations after the rAd booster vaccination are completed for all subjects. The schema is shown in the table that follows:

VRC 008		6-plasmid DNA prime vaccinations (at least 21 days between vacs.)				rAd booster vaccination	
**Subject Description**	**Number**	**DNA Vac Device**	**Day 0**	**Day 28±7**	**Day 56±7**	**Number**	**Day 168 (-7, +14 days)**
Subjects with pre-enrollment low Ad5Ab titer (≤1:500)	10	Biojector	4 mg	4 mg	4 mg	5	10^{10} PU
						5	10^{11} PU
	10	N/S	4 mg	4 mg	4 mg	5	10^{10} PU
						5	10^{11} PU
Subjects with pre-enrollment high Ad5Ab titer (>1:500)	10	Biojector	4 mg	4 mg	4 mg	5	10^{10} PU
						5	10^{11} PU
	10	N/S	4 mg	4 mg	4 mg	5	10^{10} PU
						5	10^{11} PU

4.1 STUDY POPULATION

All study activities will be carried out at the National Institutes of Health. Forty healthy, HIV-

negative volunteers will be recruited through IRB-approved advertising and will be screened through VRC 000 (02-I-0127), a screening protocol for healthy volunteers who are interested in participating in HIV vaccine clinical trials, to confirm eligibility requirements for participation. The screening and education process required prior to enrollment should ensure that subjects comprehend the purpose and details of the study. This Phase I study to establish safety of the prime-boost vaccination schedule in healthy individuals will be limited to adults who are 18-50 years old at the time of enrollment.

Prior to signing the VRC 008 informed consent, eligible volunteers will take a short "Assessment of Understanding" quiz to test understanding of this vaccine study. Incorrect answers will be explained to the volunteer and they will sign the informed consent document only after the study coordinator is satisfied with their understanding of the study.

4.1.1 Inclusion Criteria

***A participant must meet all of the following criteria*:**

1. 18 to 50 years old.
2. Available for clinical follow-up through Week 42 of the study.
3. Able to provide proof of identity to the satisfaction of the study clinician completing the enrollment process.
4. Complete an Assessment of Understanding prior to enrollment and verbalize understanding of all questions answered incorrectly.
5. Able and willing to complete the informed consent process.
6. Willing to receive HIV test results and willing to abide by NIH guidelines for partner notification of positive HIV results.
7. Willing to donate blood for sample storage to be used for future research.
8. Willing to discuss HIV infection risks and amenable to risk reduction counseling.
9. In good general health without clinically significant medical history.
10. Physical examination and laboratory results without clinically significant findings and a body mass index (BMI) less than 40 within the 28 days prior to enrollment.

Laboratory Criteria within 28 days prior to enrollment:

11. Hemoglobin ≥ 11.5 g/dL for women; ≥13.5 g/dL for men.
12. White blood cells (WBC) = 3,300-12,000 cells/mm^3.

13. Differential either within institutional normal range or accompanied by site physician approval.

14. Total lymphocyte count ≥ 800 cells/mm^3.

15. Platelets = 125,000 – 550,000/mm^3.
16. Alanine aminotransferase (ALT) ≤ 1.25 x upper limit of normal.

17. Serum creatinine ≤ upper limit of normal.

18. Normal urinalysis defined as negative glucose, negative or trace protein, and no clinically significant blood in the urine.

19. Negative Food and Drug Administration (FDA)-approved HIV blood test.

20. Negative Hepatitis B surface antigen.

21. Negative anti-HCV (hepatitis C virus antibody) and negative HCV PCR.

Female-Specific Criteria:

22. Negative β-HCG (human chorionic gonadotrophin) pregnancy test (urine or serum) on day of enrollment for women presumed to be of reproductive potential.

23. A female participant must meet any of the following criteria:

    No reproductive potential because of menopause [one year without menses] or because of a hysterectomy, bilateral oophorectomy, or tubal ligation,

    or

    Participant agrees to be heterosexually inactive at least 21 days prior to enrollment and through Week 42 of the study,

    or

    Participant agrees to consistently practice contraception at least 21 days prior to enrollment and through Week 42 of the study by one of the following methods:
    - condoms, male or female, with or without a spermicide
    - diaphragm or cervical cap with spermicide
    - intrauterine device
    - contraceptive pills or patch, Norplant, Depo-Provera or other FDA-approved contraceptive method
    - male partner has previously undergone a vasectomy for which there is documentation.

4.1.2 Exclusion Criteria

A volunteer will be excluded if one or more of the following conditions apply:

Women:

1. Woman who is breast-feeding or planning to become pregnant during the 42 weeks of study participation.

Volunteer has received any of the following substances:

2. HIV vaccine in a prior clinical trial.
3. Immunosuppressive medications or cytotoxic medications or inhaled corticosteroids within the past six months (with the exception of corticosteroid nasal spray for allergic rhinitis or topical corticosteroids for an acute uncomplicated dermatitis).
4. Blood products within 120 days prior to HIV screening.
5. Immunoglobulin within 60 days prior to HIV screening.
6. Investigational research agents within 30 days prior to initial study vaccine administration.
7. Live attenuated vaccines within 30 days prior to initial study vaccine administration.
8. Medically indicated subunit or killed vaccines, e.g. influenza, pneumococcal, or allergy treatment with antigen injections, within 14 days of study vaccine administration.
9. Current anti-tuberculosis prophylaxis or therapy.

Volunteer has a history of any of the following clinically significant conditions:

10. Serious adverse reactions to vaccines such as anaphylaxis, hives, respiratory difficulty, angioedema, or abdominal pain.
11. Autoimmune disease or immunodeficiency.
12. Asthma that is unstable or required emergent care, urgent care, hospitalization or intubation during the past two years or that requires the use of oral or intravenous corticosteroids.
13. Diabetes mellitus (type I or II), with the exception of gestational diabetes.
14. History of thyroidectomy or thyroid disease that required medication within the past 12 months.
15. Serious angioedema episodes within the previous 3 years or requiring medication in the previous two years.
16. Hypertension that is not well controlled by medication or is more than 145/95 at enrollment.
17. Bleeding disorder diagnosed by a doctor (e.g. factor deficiency, coagulopathy, or platelet disorder requiring special precautions) or significant bruising or bleeding difficulties with IM injections or blood draws.

18. Syphilis infection that is active or a positive serology due to a syphilis infection treated less than six months ago.

19. Malignancy that is active or treated malignancy for which there is not *reasonable* assurance of sustained cure or malignancy that is likely to recur during the period of the study.

20. Seizure disorder other than: 1) febrile seizures under the age of two, 2) seizures secondary to alcohol withdrawal more than 3 years ago, or 3) a singular seizure not requiring treatment within the last 3 years.

21. Asplenia, functional asplenia or any condition resulting in the absence or removal of the spleen.

22. Psychiatric condition that precludes compliance with the protocol; past or present psychoses; past or present bipolar disorder; disorder requiring lithium; or within five years prior to enrollment, history of a suicide plan or attempt.

23. Any medical, psychiatric, social condition, occupational reason or other responsibility that, in the judgment of the investigator, is a contraindication to protocol participation or impairs a volunteer's ability to give informed consent.

24. A subject with 3 or more of the 5 health risk factors noted below will be excluded:
    · Current smoker (or quit smoking less than 28 days prior to enrollment)
    · BMI >35
    · Fasting low density lipoprotein (LDL) > 159 mg/dL <u>or</u> fasting cholesterol >239 mg/dL
    · Systolic blood pressure >140 mm Hg or diastolic blood pressure >90 mm Hg
    · Fasting blood glucose >125 mg/dL

    <u>Note</u>: The fasting blood tests require 8 hours fast prior to the blood draw. The results used for eligibility screening must be from tests completed no more than 12 weeks (84 days) prior to day of enrollment. The individual criteria for BMI (inclusion item 10) and blood pressure (exclusion item 16) must also be met.

4.2 SCHEDULE OF CLINICAL PROCEDURES AND LABORATORY ASSAYS

Evaluation of the safety of this vaccine will include laboratory studies, medical history, physical assessment by clinicians, and subject self-assessment recorded on a diary card. Potential adverse reactions will be further evaluated prior to continuing the immunization schedule. Blood tests for immune responses will be performed at the Vaccine Research Center. The study schedule is described in Section 4.2.2 and presented in the form of a Table in Appendix III. Total blood volume drawn from each subject will not exceed the NIH Clinical Center Guidelines of 450 mL in any 6-week period.

4.2.1 <u>Screening</u>

Screening for this study will be completed through the Vaccine Research Center's Screening Protocol, VRC 000 (NIH 02-I-0127). The evaluations and sample collection that will be included in VRC 000 screening are a medical history, physical exam, complete blood count with

differential, prothrombin time (PT), partial thromboplastin time (PTT), chemistry panel (including fasting glucose, cholesterol and LDL), quantitative immunoglobulins, rapid plasma reagin (RPR), hepatitis B surface antigen, anti-hepatitis C antibody, HCV PCR, anti-dsDNA, HIV ELISA/Western Blot, HIV PCR, T-cell subsets, adenovirus serology, urinalysis, pregnancy test (for females of reproductive potential), and questions regarding sexual behavior and other practices. The adenovirus serology used for randomization, as well as the fasting glucose, cholesterol and LDL, used in the eligibility screening must be from within the 12 weeks (84 days) prior to enrollment. Any test that has a specific eligibility requirement must be done within the window needed to meet study eligibility. Risk status for HIV infection will be determined by a series of questions designed to identify risk factors. Storage samples of peripheral blood mononuclear cells (PBMCs) and serum will also be collected. General eligibility for clinical trials will be dependent on results of laboratory tests and answers to the interview questions. Informed consent documents for vaccine trials will be reviewed, and counseling relating to the potential risks of becoming pregnant during this trial and avoiding HIV infection will be provided. An Assessment of Understanding of VRC 008 is completed on the day the subject is scheduled to enroll in VRC 008.

4.2.2 Day 0 through Week 42 Clinical Follow-Up and Week 94 Follow-up

Day 0 is defined as the day of VRC 008 enrollment and first injection. VRC 008-specific eligibility is reviewed on Day 0 as part of the enrollment process. Subjects will begin the prime-boost schedule on the day of enrollment with the first DNA vaccination. Pregnancy test results for women of reproductive potential must be negative and be obtained on each injection day prior to the study injection. Day 0 evaluations prior to the first injection are the baseline for subsequent safety assessments. Refer to the itemized list in this section and the table in Appendix III for details on when each type of evaluation must be completed.

The schedule for DNA vaccinations is Day 0, Day 28 ± 7, Day 56 ± 7 (with at least 21 days between injection days). The rAd booster vaccination will be scheduled as close to Day 168 as possible, with a -7 days to +14 days window permitted for scheduling.

All VRC-HIVDNA016-00-VP vaccinations will be administered into the deltoid muscle at a 4 mg dose using either a needle and syringe or Biojector needle-free injection system according to randomization assignment. Neither clinic staff nor subjects will know in advance the device randomization that is assigned to the sequential enrollees in the study. The injection device to be used will be known to both clinic staff and the subject after completing the subject's enrollment into the study.

All VRC-HIVADV014-00-VP vaccinations will be administered into the deltoid muscle using a needle and syringe. Subjects will be randomly assigned to either 10^{10} PU or 10^{11} PU rAd boost dose. This dose assignment will be blinded to clinic staff and subjects until all subjects have completed the safety and immunogenicity evaluations that are 6 weeks after the booster injection.

The Protocol Statistician will prepare the randomization plan for the DNA vaccination injection device and the rAd vaccination booster dose and provide it to the Site Pharmacy where the injections will be prepared.

Prior to each DNA vaccination, a photo of the deltoid area to be injected will be taken.

Following each study injection (DNA vaccine or rAd vaccine), subjects will be observed for a minimum of 30 minutes. Vital signs (temperature, blood pressure, pulse and respiratory rate) will be completed between 30 and 45 minutes post-immunization and the injection site will be inspected for evidence of local reaction. Subjects will be given a "Diary Card" on which to record temperature and symptoms daily for 5 days.

Follow-up for DNA vaccinations: Subjects will have a clinic visit at 3(±1) days following each DNA injection. These visits will include interim history, vital signs, lymph node exam, and examination of the vaccination site. Erythema, induration or skin lesions will be documented by measurement of perpendicular diameters. A photograph of the vaccination site will be taken whether or not there are any findings. If there is evidence of a skin lesion, the visit at 7(±1) days after vaccination will also be a clinic visit; otherwise follow-up will be first by telephone with a clinic visit scheduled if the subject reports changes suggesting a skin lesion may be forming. Attempt will be made to photograph the vaccination site at the same distance each time with similar lighting conditions. A tag with metric ruler, subject number, date, and visit identifiers will be placed below the vaccination site prior to photographing. The Diary Card will be collected at the first clinic visit after each DNA vaccination when it is complete.

At 14(±3) days after each DNA injection, study subjects will be evaluated at a clinic visit that includes interim history, vital signs, lymph node exam, examination of the vaccination site, and collection of urine and blood samples. A photograph will be taken if there is a skin lesion or need to document resolution of any findings that were present at a prior visit.

Following a DNA vaccine injection, a skin biopsy is an optional assessment if a skin lesion appears to be forming. Skin biopsies are included in this study for research purposes to better understand the etiology (i.e., distinguish between immune, allergic, infectious or foreign body reaction) of skin lesions sometimes associated with the DNA vaccine. Subjects may refuse to have a skin biopsy. The preferred timepoint for obtaining a skin biopsy is at the earliest visit after a vaccination where there is evidence of a skin lesion. This is expected to be 3(±1) day post-vaccination in most cases, but may be earlier or later depending upon individual circumstances. Subjects will not be asked to have more than two skin biopsies of DNA vaccination site skin lesions. However, if a skin biopsy is recommended for clinical care purposes, additional skin biopsies may be done with subject consent.

The follow-up visit at Week 12 (±7) days includes interim history, vital signs, lymph node exam, and collection of blood samples.

Follow-up for rAd vaccinations: The first follow-up will be performed by telephone on the first or second day following the rAd injection. A clinic visit will occur if indicated by the telephone interview. Events reported in the telephone interview that will require a clinic visit include rash, urticaria (hives), fever of 38.7˚C (Grade 2) or higher, or significant impairment in the activities of daily living (ADL). At 14(±3) days after rAd injection, study subjects will be evaluated at a clinic visit. This visit will include interim history, vital signs, lymph node exam and examination of the vaccination site. The Diary Card will be collected.

Other follow-up visits are Week 28(±7 days), Week 30(±7 days) and Week 42(±14 days). Note that if the rAd injection is delayed to Days 176 through 182 then the Week 28 and Week 30 visits may be adjusted further, if needed to keep the interval after the rAd injection close to 4 weeks and 6 weeks post injection, respectively. The schedule of follow-up visits is shown in the

table in Appendix III. Subjects are followed in the clinic until Week 42(±14 days). This will be about 18 weeks of follow-up after the rAd booster vaccine.

At intervals throughout the study subjects will have blood drawn for immunologic assays. Any cells, serum or plasma not used will be stored for future virological and immunological assays. Subjects will also be interviewed at the final clinical visit regarding social harms, including problems with employment, travel, immigration, access to insurance, medical or dental care, and negative reactions from family, friends, and co-workers. Study visit procedures and tests through last clinic visit are as follows:

- "VRC 008 Assessment of Understanding" Quiz (Day 0)
- Signature of study participation informed consent form for VRC 008 (Day 0)
- Clinical evaluations: vital signs and weight (every visit day); axillary lymph node exam (vaccination visits and follow-up visits through 4 weeks after each vaccination); targeted physical exam on any visit if indicated by interim complaints or laboratory findings.
- Interim medical history (every visit).
- Counseling on HIV and avoidance of pregnancy (Day 0; offered every subsequent visit)
- Study injections (refer to study schema)
- Post-injection vital signs and assessment of injection site at 30 to 45 minutes after a study injection
- Diary Card: Baseline on day of injection; 5-day diary card for self-assessment by subject following each injection. The diary card will include the parameters: unusually tired/feeling unwell, muscles aches (at other than injection site), headache, chills, nausea, and pain/tenderness at injection site. Subjects will also record highest measured temperature, measurement of perpendicular diameters for redness and swelling at injection site and note if there is evidence of a skin lesion at the vaccination site. The diary cards are collected at the earliest clinic visit after each injection when the card is complete.
- Photograph of DNA vaccination site: required prior to the vaccination for baseline and at 3±1 days following each DNA injection regardless of presence or absence of vaccination site findings. Continue to photograph vaccination site at subsequent visit until resolution of any skin lesion or other significant findings.
- DNA vaccination site skin biopsy: optional assessment of DNA vaccination sites with skin lesion. Limited to two per subject unless medically indicated for clinical care purposes.
- Serum or urine pregnancy test, for females of reproductive potential (vaccination visits and last clinic visit)
- HLA (Week 2)
- CBC, differential, platelet count (Day 0 and Weeks 2, 4, 6, 8, 10, 12, 24, 26, 28, 30 and 42)
- Creatinine and ALT (Day 0 and Weeks 2, 4, 6, 8, 10, 12, 24, 26, 28, 30 and 42)
- Urinalysis (Day 0 and Weeks 2, 4, 6, 8, 10, 12, 24 and 26)
- T cell FACS for CD4/CD8 (Day 0 and Weeks 12, 24, 30 and 42)

- HIV testing: ELISA (also Western blot if serology is positive) and HIV PCR (Day 0 and Weeks 12, 24, 30 and 42)
- HIV specific antibody assays (Day 0 and Weeks 8, 12, 24, 28 and 42). Note: The assays will not be performed immediately, but rather completed using frozen samples at a later date.
- Intracellular cytokine staining (ICS) and ELISPOT assays (Day 0 and Weeks 6, 8, 10, 12, 24, 28, 30 and 42). Note: The assays will not be performed immediately, but rather completed using frozen samples at a later date. PBMC and plasma for storage will be saved from the blood collected for these assays.
- Social Impact Questionnaire (Week 42). The Social Impact questionnaire will include parameters: personal relationships, travel or immigration, employment, education, medical or dental, health insurance, life insurance, housing, military/other government agency and other.
- Serum for archiving (Day 0 and Weeks 4, 6, 8, 10, 12, 24, 28, 30 and 42)
- Adenovirus Serology (Day 0 and Weeks 24, 28 and 42)

Week 94 Long-term Follow-up:

After the Week 42 clinic visit, subjects will be contacted one year later. Subjects will be encouraged to return for a clinic visit at Week 94 (± 28 days) to be interviewed about any interval life-threatening adverse events, persistent or significant disability/incapacity, non-elective hospitalizations, new chronic diseases requiring ongoing medical management or medication or outcomes of any pregnancies (including if there were any congenital anomalies/birth defects), as well as to have HIV testing (ELISA with Western blot if positive and HIV PCR) and for a research immunology blood draw (PBMC, plasma and serum). A subject may opt to be contacted only by telephone, mail or e-mail to allow collection of the interview information specified above without a follow-up blood draw. If there are any subject deaths in the interval between Week 42 and Week 94, an attempt will be made to obtain information about cause of death. Follow-up testing for vaccine-induced HIV antibody is permitted for up to 5 years after the Week 42 visit (see Section 4.3). Subjects may also be contacted at other times to confirm contact information, provide notification of release of study results and when the information on rAd vaccine dose assignment is ready to be made known to the subject.

4.3 MONITORING FOR HIV INFECTION

It is possible that this vaccination regimen will induce immunologic responses that are detected by standard HIV screening techniques, even though the vaccines will not cause HIV infection. The following steps will be taken to ensure detection of HIV infection and to protect participants from adverse consequences associated with an HIV antibody test that indicates an antibody response to the vaccine:

- Study participants will receive regularly scheduled counseling regarding avoidance of HIV infection in accordance with the most recent CDC HIV Counseling Guidelines.
- Study participants will be screened for HIV infection periodically while participating in the study (see Appendix III for schedule of testing).

- If there is any clinical or laboratory indication of HIV infection, any test required to make a definitive diagnosis, including Western blot analysis, viral load measurement (PCR), or other tests will be performed.

- Confirming tests will be performed as soon as possible once a positive antibody response is identified. Participants will be promptly informed if they are HIV-infected. Participants who are found to have vaccine-induced antibody responses, but with no evidence of HIV infection, will be informed that they are not HIV-infected. Written documentation describing any vaccine-induced antibody response and confirming data will be provided when the study is completed. This should be sufficient evidence that the antibody response as of the date of testing resulted from vaccination and not naturally occurring infection. Participants with vaccine-induced antibody will be provided with the opportunity for HIV antibody testing annually for five years to monitor their serological status. Participants will be counseled regarding the potential for antibody responses and the implications of such responses prior to participation in the study.

4.4 INTERCURRENT HIV INFECTION

The vaccine cannot cause HIV infection. Subjects who become HIV infected due to other causes while participating in the study will be referred for medical care for treatment and management of the disease. They may be given the opportunity to enroll in an appropriate study of acute HIV infection or a long-term follow-up study, if one is available. The NIH investigators will not be responsible for providing ongoing medical care or antiretroviral medications in the event of HIV-1 infection.

4.5 CONCOMITANT MEDICATIONS

Concomitant medications are recorded at screening and every study visit. The concomitant medications eligibility criteria for enrollment continue to apply for the subject to remain eligible for each study injection. If an enrolled subject develops the need for a medication that is prohibited by the eligibility criteria, then further study injections will be discontinued. If an FDA-approved live attenuated vaccine is required for an immediate medical need, then study injections must be discontinued. If an FDA-approved subunit or killed vaccine is required for an immediate medical need, then it must be given at least 14 days before or 14 days after any study injection for the subject to remain eligible for additional study injections. If it will not imperil a subject's health, FDA-approved vaccines should be deferred until at least 30 days after the final study injection. Any subject who receives at least one study injection will continue with the clinical and laboratory evaluations specified by the study through the 12 months of follow-up.

4.6 CRITERIA FOR WITHDRAWAL OF A SUBJECT FROM INJECTION SCHEDULE

Under certain circumstances, a subject will be terminated from participating in further injections. Participants who are discontinued from additional study vaccinations will continue to be followed according to the schedule of safety and immunogenicity evaluations, except that the follow-up evaluations that are specifically for safety follow-up on a vaccination do not need to be completed when a vaccination is not given. Referring to Appendix III, these are the "A", "B" and "C" visits that follow a DNA vaccination and the "A" and "B" visits that follow the rAd vaccination. Specific events that will require withdrawal of a subject from the vaccination

schedule include:

1. HIV infection;
2. Pregnancy;
3. Grade 2 adverse event classified as possibly associated with immunization that does not resolve to baseline in time for the next scheduled immunization;
4. Grade 2 adverse event classified as probably or definitely associated with immunization (with the exception of grade 2 pain/tenderness, fatigue/malaise, nausea, headache, chills or myalgia);
5. Grade 3 or 4 systemic or injection site adverse event classified as possibly, probably or definitely associated with immunization;
6. Type 1 hypersensitivity associated with immunization;
7. Serious intercurrent illness that is not expected to resolve prior to the next scheduled immunization;
8. Treatment with systemic glucocorticoids (e.g., prednisone or other glucocorticoid) or other immunomodulators (other than NSAIDs) for any reason;
9. Medical need for concomitant vaccine during the period of study vaccinations that requires discontinuation from the study vaccination schedule (see section 4.5);
10. Repeated failure to comply with protocol requirements;
11. The IND sponsor, study sponsor or Principal Investigator decides to stop or cancel the study;
12. The IRB or the FDA request that the study be stopped.

4.7 CRITERIA FOR STOPPING STUDY (AS AMENDED NOVEMBER 9, 2005)

The Principal Investigator will closely monitor and analyze study data as they become available and will make determinations regarding the presence and severity of adverse events. The DAIDS Medical Officer will provide an independent review of adverse events that have a bearing on study stopping. Following review with the FDA (on November 9, 2005) of a halt in rAd booster vaccinations because of a Grade 3 fever, it was agreed that the protocol does not need to be halted for the signs and symptoms consistent with the self-limited fever and flu-like syndrome that may occur in the rAd post-vaccination period. Therefore, this protocol section is amended.

Any Grade 2 or Grade 3 post-vaccination reactogenicity adverse events of pain/tenderness, fever, malaise, fatigue, headache, chills, nausea, myalgia, or arthralgia will be reviewed by the IND Sponsor's Medical Officer and the Principal Investigator at weekly safety monitoring review (see Section 8.9) while vaccinations are ongoing. A quarterly safety monitoring report will be submitted to the IND until all subjects have completed at least 4 weeks of follow-up of the rAd booster vaccination.

The administration of study injections and new enrollments will be halted and the IND sponsor promptly notified according to the criteria that follow. Counting of events of the types listed is done for each investigational vaccine separately.

- **One** (or more) subject experiences a Grade 4 adverse event that is assessed as possibly, probably or definitely related to a study vaccine;

  OR

- **One** (or more) subject experiences a Grade 3 adverse event assessed as possibly, probably or definitely related to a study vaccine: this criterion applies to erythema, induration, vomiting, laboratory abnormalities or other clinical adverse experiences, but does not apply to the local or systemic post-vaccination adverse events of pain/tenderness, fever, malaise, fatigue, headache, chills, nausea, myalgia, or arthralgia;

  OR

- **One** (or more) subject experiences Grade 2 erythema or induration at an injection site;

  OR

- **Two** (or more) subjects experience the **same** Grade 2 or higher adverse event assessed as possibly, probably or definitely related to the same vaccine: this criterion applies to vomiting, laboratory abnormalities or other clinical adverse experiences, but does not apply the local or systemic post-vaccination adverse events of pain/tenderness, fever, malaise, fatigue, headache, chills, nausea, myalgia, or arthralgia.

The study injections and enrollments would resume only if review of the adverse events that caused the halt resulted in a recommendation to permit further study injections and study enrollments. The reviews to make this decision will occur as follows:

<u>Grade 2 events that meet the stopping criteria</u>: The IND Sponsor, in consultation with the Principal Investigator, will conduct the review and make the decision to resume or close the study for any Grade 2 events leading to a halt.

<u>Grade 3 or Grade 4 events that meet the stopping criteria</u>: The IND Sponsor, with participation by the Principal Investigator, will consult with the FDA to conduct the review and make the decision to resume or close the study for the Grade 3 and Grade 4 adverse events that meet the criteria for halting the study.

Safety data reports and changes in study status are submitted to the IRB promptly in accordance with Section 5.4 and institutional policy.

5. SAFETY AND ADVERSE EVENT REPORTING

5.1 ADVERSE EVENTS

An adverse event is any unfavorable or unintended change in body structure, body function or laboratory result associated temporally with the use of study treatment, whether or not considered related to the study treatment. Each adverse event will be graded according to the Table for Grading Severity of Adverse Events (see Appendix IV).

5.2 SERIOUS ADVERSE EVENTS (SAE)

The term "Serious Adverse Drug Experience" is defined in 21 CFR 312.32 as follows: "Any adverse drug experience occurring at any dose that results in any of the following outcomes: Death, a life-threatening adverse drug experience, inpatient hospitalization or prolongation of existing hospitalization, a persistent or significant disability/incapacity, or a congenital anomaly/birth defect. Important medical events that may not result in death, be life-threatening, or require hospitalization may be considered a serious adverse drug experience when, based upon appropriate medical judgment, they may jeopardize the subject or require medical or surgical intervention to prevent one of the outcomes listed in this definition. Examples of such medical events include allergic bronchospasm requiring intensive treatment in an emergency room or at home, blood dyscrasias or convulsions that do not result in inpatient hospitalization, or the development of drug dependency or drug abuse."

In Section 5.3 the term "Expedited Adverse Event" (EAE) encompasses the events that would be considered an SAE by the 21 CFR 312.32 definition.

5.3 ADVERSE EVENT REPORTING TO THE IND SPONSOR

Information on adverse events (AEs) is collected by Study Nurses and other clinic staff and entered into a computer database. The Principal Investigator and the Study Coordinator review these data on an ongoing basis.

The expedited adverse event (EAE) reporting requirements and definitions for this study and the methods for expedited reporting of AEs to the DAIDS Regulatory Compliance Center (RCC) Safety Office are defined in "The Manual for Expedited Reporting of Adverse Events to DAIDS" (DAIDS EAE Manual) dated May 6, 2004. The DAIDS EAE Manual is available on the RCC website: http://rcc.tech-res-intl.com/.

AEs reported on an expedited basis must be documented on the DAIDS Expedited Adverse Event Reporting Form (EAE Reporting Form) available on the RCC website: http://rcc.tech-res-intl.com. RCC contact information is provided in Appendix II.

EAE Reporting Level:

This study uses the Standard Level of expedited AE reporting as defined in the DAIDS EAE Manual. Briefly summarized, Standard Level reporting requires completion of an EAE report form for the following types of AEs occurring after exposure to the study agent:

- Result in death regardless of relationship to study agent.
- Are congenital anomalies, birth defects, or fetal losses regardless of relationship to study agent.
- Result in persistent or significant disabilities or incapacities regardless of relationship to study agent.
- Are a suspected adverse drug reaction (i.e., definitely, probably, possibly, or probably not related to study agent) that requires hospitalization, or prolongs existing hospitalization OR requires intervention to prevent significant/permanent disability or death.
- Are life-threatening (including all Grade 4 adverse events) suspected adverse drug reactions (i.e., assessed as definitely, probably, possibly or probably not related to study

agent).

In addition, any event, regardless of grade, which in the judgment of a site investigator represents a serious adverse event, may be reported to the IND sponsor as an EAE.

EAE Reporting Period:

AEs must be reported on an expedited basis at the Standard Level during the protocol-defined EAE Reporting Period, which for this study is from study enrollment until the last required clinical visit at Week 42 or until discontinuation of the subject from study participation for any reason.

After the end of the protocol-defined EAE reporting period stated above, the site must report serious, unexpected, clinical suspected adverse vaccine reactions if the study site staff becomes aware of the event on a passive basis, i.e. from publicly available information.

Study Agents for Expedited Reporting to DAIDS:

The study agents that must be considered when determining relationships of AEs requiring expedited reporting to DAIDS are: VRC-HIVDNA016-00-VP and VRC-HIVADV014-00-VP.

Grading Severity of Events:

The Table for Grading the Severity of Adult Adverse Events is: "The Division of AIDS Table for Grading the Severity of Adult and Pediatric Adverse Events, Version 1.0, Dec 2004" (see Appendix IV).

The EAE report must be reported on the EAE form and submitted by the clinical site to the IND sponsor (DAIDS) through the Regulatory Compliance Center (RCC) Safety Office (RCCSafetyOffice@tech-res.com) as soon as possible, but no later than 3 working days after the clinical site becomes aware of events meeting these criteria. The IND sponsor is responsible for submitting IND safety reports to the FDA, as necessary, per 21 CFR 312.32. DAIDS submits IND safety reports as soon as possible, but no later than 15 days after initial receipt of the information.

5.4 ADVERSE EVENT REPORTING TO THE INSTITUTIONAL REVIEW BOARD

Adverse event reporting requirements to the NIAID Institutional Review Board (IRB) for this protocol are as follows:

- Investigators will submit a completed serious adverse event report to the NIAID IRB within 7 days after becoming aware of a subject death, a potentially life-threatening (grade 4) serious adverse event that is possibly, probably or definitely related to investigational agent, an inpatient hospitalization (other than elective), a persistent or significant disability/incapacity, or a congenital anomaly/birth defect.

- Investigators will submit a completed serious adverse event report to the NIAID IRB within 15 days after becoming aware of any Grade 3 (severe) adverse event that is possibly, probably or definitely related to investigational agent.

- Investigators will report within 15 days on any other event or condition regardless of grade, which in their judgment represents an event reportable to the IRB.

- Investigators will forward all IND safety reports and related FDA communications to the IRB within 15 days of receipt.

- A summary of all adverse events will be reported to the NIAID IRB with submission of a request for continuing review.

5.5 SERIOUS ADVERSE EVENT REPORTING TO THE INSTITUTIONAL BIOSAFETY COMMITTEE

The Institutional Biosafety Committee (IBC) has a responsibility to review research using recombinant DNA for compliance with NIH Guidelines. In keeping with IBC requirements, any SAE reports sent to the IRB will be provided to the IBC at the same time.

6. STATISTICAL CONSIDERATIONS

6.1 OVERVIEW

This study is a single-center, randomized trial to assess the safety and tolerability of a schedule of 3 HIV DNA plasmid vaccinations followed by one either 10^{10} PU or 10^{11} PU HIV adenoviral vector (rAd) booster vaccination by either needle and syringe (N/S) or by Biojector in HIV-uninfected adults. A preliminary assessment of immunogenicity will also be performed.

6.2 OBJECTIVES

The primary objective is to evaluate the safety and tolerability in humans of the prime-boost vaccination regimen. Secondary objectives include evaluating the immunogenicity of the vaccination regimen, the development of adenovirus serotype 5 neutralizing antibody and the social impact of participating in an HIV-1 vaccine trial. The study will provide preliminary information on whether both devices used for DNA vaccinations result in similar safety and immunogenicity and whether pre-enrollment adenovirus serotype 5 antibody (Ad5Ab) titer affects safety of or immune response to the rAd booster vaccination.

6.3 ENDPOINTS

6.3.1 Safety

Assessment of product safety will include clinical observation and monitoring of hematological and chemical parameters. Safety will be closely monitored after injection and evaluated through 18 weeks following the adenoviral vector booster vaccination. See Section 4.2 and Appendix III for details and specified time points. The following parameters will be assessed:

- Local reactogenicity signs and symptoms
- Systemic reactogenicity signs and symptoms
- Laboratory measures of safety
- Adverse and serious adverse experiences

6.3.2 Immunogenicity

The principal immunogenicity endpoints for cellular immune responses are measured at Week 0 (baseline), 4 weeks after third DNA vaccination and 6 weeks after Ad vaccine booster. They will consist of HIV-1-specific T cell responses, as measured by intracellular cytokine staining (ICS) and ELISPOT assays. T cell assays at other study timepoints, as well as HIV-1-specific humoral immune responses as measured by HIV-specific antibody assays will be completed as exploratory evaluations.

6.3.3 Social Impacts

Social impact variables, as measured by questionnaire at the last clinic visit, include any negative experiences or problems the participant experienced due to his/her participation in this study. The following social impacts will be followed during the course of the study: personal relationships, travel or immigration, employment, education, medical or dental care, health insurance, life insurance, housing, military/other government agency and other impacts identified by a participant.

6.4 SAMPLE SIZE AND ACCRUAL

Recruitment will target 40 healthy, HIV-uninfected adult participants between age 18 and 50 years old. The study plan is that all subjects receive three injections with 4 mg of the investigational vaccine, VRC-HIVDNA016-00-VP, by one of two methods, either a needle and syringe or Biojector needleless injection system followed by a booster vaccination with VRC-HIVADV014-00-VP at Week 24. The required clinical follow-up is through Study Week 42. Sample size will be 40 subjects with safety data for the prime-boost regimen. For other evaluations, using a factorial design, the following samples sizes apply:

Biojector DNA vaccine primes: N = 20
Needle and Syringe DNA vaccine primes: N=20
10^{10} PU rAd booster vaccination: N=20
10^{11} PU rAd booster vaccination: N=20

6.4.1 Randomization of Treatment Assignments

The randomization sequence will be obtained by computer-generated random numbers and provided to the study pharmacist by the statistician. Study numbers 01008001 through 01008020 will be used for the randomization of subjects with low Ad5Ab titer (≤1:500) and study numbers 01008021 through 01008040 will be used for the randomization of subjects with high Ad5Ab titer (>1:500); within each stratum study numbers have been randomly assigned for the combination of DNA injection device and rAd dose the subject will receive. Subjects with both high and low Ad5Ab titer will be enrolled simultaneously. The pharmacist and the statistician are responsible for maintaining security of the treatment assignments. To maintain blinding, any discussion of the treatment assignment between the VRC clinicians and the pharmacy staff is prohibited until after the assignments are permitted to be known to all.

To decrease the potential for participant dropouts during the period between randomization and initial vaccination, randomization will occur on Day 0 after the study consent is signed and eligibility is confirmed. The study number is assigned through completion of the eligibility checklist in the electronic study database and will be the next sequential number in the study number sequence for the subject’s Ad5Ab titer category. The assignment to Biojector or

needle/syringe for the DNA vaccinations will become known to subjects and protocol staff shortly after study enrollment, as the device used for DNA vaccinations will be evident. The rAd boost dosage will be blinded until 6 weeks of safety and immunogenicity evaluations after the rAd boost are completed for all subjects who receive an rAd boost. At that point all assessments of vaccine acute reactogenicity and other short term adverse events will be complete and knowledge of the rAd dosage will not influence the reactogenicity assessments.

6.4.2 Power Calculations for Safety

The goal of the safety evaluation for this study is to identify safety concerns associated with injection. Sample size calculations for safety are expressed in terms of the ability to detect serious adverse experiences.

The ability of the study to identify serious adverse experiences is best expressed by the maximum true rate of events that would be unlikely to be observed and the minimum true rate of events that would very likely be observed. Specifically, there is a 90% chance of observing at least 1 serious adverse experience in the 40 volunteers if the true rate of such an event is at least 0.055; there is a 90% chance that we would not observe at least 1 serious adverse experience if the true rate is less than 0.003. Probabilities of observing 0 or 2 or more serious adverse experiences among a sample size N=40 are presented in Table 6.1 for a range of possible true event rates. These calculations provide a more complete picture of the sensitivity of this study design to identify potential safety problems with the vaccine.

Table 6-1: Probability of response for different safety and immunogenicity scenarios

True Event rate	Pr(0/40)	Pr(2+/40)
0.001	0.961	0.001
0.003	0.887	0.007
0.005	0.818	0.017
0.010	0.669	0.061
0.030	0.296	0.338
0.050	0.129	0.601
0.075	0.044	0.812
0.100	0.015	0.920
0.150	0.002	0.988
0.200	<.001	0.999

Table 6-2 gives the upper and lower bounds for 95% exact binomial confidence intervals for all possible numbers of events; since binomial probabilities are symmetric, confidence intervals for numbers of events greater than 20 can be calculated using the proportion of participants without events and subtracting the corresponding 95% confidence interval from 1. For example, if none of the 40 participants receiving the vaccine experience serious adverse experiences to the vaccine, the 95% exact 2-sided upper confidence bound for the rate of such reactions in the population is 0.088, whereas if all 40 had events the interval would be from 0.912 to 1.

Table 6-2: 95% Confidence Intervals for all possible observed rates

	95% CI		95% CI		95% CI
0/40	0, 0.088	7/40	0.073, 0.328	14/40	0.206,0.517
1/40	0.001, 0.132	8/40	0.091, 0.356	15/40	0.227,0.542
2/40	0.006, 0.169	9/40	0.108, 0.385	16/40	0.249, 0.567
3/40	0.016, 0.204	10/40	0.127, 0.412	17/40	0.270, 0.591
4/40	0.028, 0.237	11/40	0.146, 0.439	18/40	0.293, 0.615
5/40	0.042, 0.268	12/40	0.166, 0.466	19/40	0.315, 0.639
6/40	0.057, 0.298	13/40	0.186, 0.491	20/40	0.338, 0.632

6.4.3 Sample Size Calculations for Immunogenicity

The primary goal of this trial regarding immunogenicity outcomes is a preliminary estimation of response rates. The definition of response is based on comparing the percent of responding cells when stimulated to the background levels specific for each person at each time point. A statistical test is used to determine if the percent of responding cells is significantly higher than background; if so this is considered a response at this time point. A 1% false-positive rate is built into the statistical criteria and the methods were validated on both HIV-positive and HIV-negative samples. Table 6-2 is applicable to the immunogenic response rates, and gives the exact 95% confidence interval for any possible number of responses out of the 40 volunteers. For example, if we observe 15 responses among the vaccinees, our 95% exact binomial confidence interval for the true rate will range from 0.227 to 0.542.

There is also interest in estimating the immunological response among the vaccinees who receive each of the two methods of injection, as well as among those receiving each dosage of the rAd vaccine, and among the two strata defined by Ad5Ab titer. The response rates in each of these groups will be based on 20 participants.

Table 6-3 gives exact 95% confidence intervals for all possible numbers of responders out of twenty participants.

Table 6-3: 95% Confidence Intervals for all possible observed rates in strata

	95% CI		95% CI		95% CI
0/20	0, 0.168	7/20	0.154, 0.592	14/20	0.457, 0.881
1/20	0.001, 0.249	8/20	0.191, 0.639	15/20	0.509, 0.903
2/20	0.012, 0.317	9/20	0.231, 0.685	16/20	0.563, 0.943
3/20	0.032, 0.379	10/20	0.272, 0.728	17/20	0.621, 0.968
4/20	0.057, 0.437	11/20	0.315, 0.769	18/20	0.683, 0.988
5/20	0.087, 0.491	12/20	0.361, 0.809	19/20	0.751, 0.999
6/20	0.119, 0.543	13/20	0.408, 0.846	20/20	0.832, 1

This study will not be powered to detect differences between the groups and would be unlikely to declare statistical significance unless the true difference was extreme.

Table 6-4 gives the power to detect a difference for various proportions in two groups of size 20; to have at least 80% power to detect a difference, we would need to expect a difference of approximately 50% between the two groups.

Table 6-4: Power to detect difference in two groups of size 20 based on underlying proportions

	Group 1 proportion						
Group 2 proportion		.4	.5	.6	.7	.8	.9
	.1	.50	.75	.90	.97	.99	>.99
	.2	.19	.40	.65	.84	.96	.99
	.3	.05	.16	.34	.59	.84	.97
	.4		.04	.14	.34	.65	.90
	.5			.04	.16	.40	.74

6.5 STATISTICAL ANALYSIS

Since enrollment is concurrent with receiving the first study vaccination, all participants will have received at least one vaccination and therefore will provide some safety data.

All statistical analyses will be performed using SAS and S-Plus statistical software.

No formal multiple comparison adjustments will be employed for safety endpoints or secondary endpoints.

6.5.1 Analysis Variables

The analysis variables consist of baseline variables, safety variables, immunogenicity and social

impact variables for primary and secondary objective analyses.

6.5.2 Baseline Demographics

Baseline characteristics including demographics and laboratory measurements will be summarized using descriptive statistics.

6.5.3 Safety Analysis

Reactogenicities

The number and percentage of participants experiencing each type of reactogenicity sign or symptom will be tabulated by severity. For a given sign or symptom, each participant's reactogenicity will be counted once under the maximum severity for all assessments.

Adverse Experiences

Adverse experiences (AEs) are coded into MedDRA preferred terms. The number and percentages of participants experiencing each specific adverse event will be tabulated by severity and relationship to treatment. For the calculations in these tables, each participant's adverse experience will be counted once under the maximum severity or strongest recorded causal relationship to treatment.

Adverse experiences following vaccination with VRC-HIVDNA016-00-VP by needle and syringe will be summarized separately from AEs following vaccination with VRC-HIVDNA016-00-VP by Biojector. Adverse experiences occurring after the VRC-HIVADV014-00-VP booster vaccination through 18 weeks after the booster vaccination will be summarized separately.
A complete listing of adverse experiences for each participant will provide details including severity, relationship to treatment type, onset, duration and outcome.

Local laboratory values

Boxplots of local laboratory values will be generated for baseline values and for values measured during the course of the study. Each boxplot will show the 1st quartile, the median, and the 3rd quartile. Outliers, or values outside the boxplot, will also be plotted. If appropriate, horizontal lines representing boundaries for abnormal values will be plotted.

6.5.4 Immunogenicity Analysis

The statistical analysis for immunogenicity will employ the intent-to-treat principle, i.e., all data from enrolled participants will be used. The only exception will be to exclude data from HIV-infected participants at or post infection. If the HIV positivity status of an infected participant is unknown at the time that the first sample for immunogenicity assessments is drawn, then all data from that participant will be excluded from the analysis.

If assay data are qualitative (i.e., positive or negative) then analyses will be performed by tabulating the frequency of positive response for each assay at each time point that an assessment is performed. Binomial response rates will be presented with their corresponding exact 95% confidence interval estimates. Response rates by strata, injection method and Ad boost will be summarized in contingency tables and compared using Fisher's exact test. Missing responses will be assumed to be missing at random, i.e., conditional on the observed data the missingness is independent of the unobserved responses. Graphical descriptions of the longitudinal immune

responses will also be given.

Some immunologic assays have underlying continuous or count-type readout that is often dichotomized into responder/nonresponder categories. For these assays, graphical and tabular summaries of the underlying distributions will be made. These summaries may be performed on transformed data (e.g., log transformation) to better satisfy assumptions of symmetry and homoscedasticity.

6.5.5 Social Impact analysis

Social impacts will be tabulated by type of event and impact on quality of life. The number and percentage of participants experiencing each type of social impact will also be tabulated by impact on quality of life. For this calculation multiple events of the same type for a participant will be counted once under the maximum impact for all post-vaccination visits.

In addition, a listing will be generated of all participants who experienced a major disturbance of their quality of life due to study participation. The listing includes all social impacts experienced by these participants, descriptions of each impact, impact on quality of life and whether or not there was a resolution.

6.5.6 Interim analyses

Interim analyses of immunogenicity will be performed after all ICS assays up to and including 4 weeks after completion of the priming vaccination have been completed on all participants and again 6 weeks after completion of the booster vaccinations have been completed on all participants. The purpose of the reports is to provide basic immunogenicity data to inform those who are making future clinical trial development-related decisions in a timely manner. The device used for DNA vaccination is not blinded. The protocol plan states that the dose of rAd used for each subject will be unblinded when all have completed six weeks of follow-up on the rAd vaccination. The results of this interim immunogenicity analysis will in no way influence the conduct of the VRC 008 trial in terms of early termination or completion of later safety or immunogenicity endpoint assessments.

7. PHARMACY PROCEDURES

7.1 STUDY AGENTS FOR PRIME-BOOST REGIMEN

7.1.1 DNA 6-Plasmid Vaccine, VRC-HIVDNA016-00-VP

The investigational DNA plasmid vaccine, VRC-HIVDNA016-00-VP, is produced under current Good Manufacturing Practices (cGMP) conditions by Vical Incorporated (San Diego, CA). It is composed of six closed circular plasmid DNA macromolecules. Plasmids VRC 4401, VRC 4409 and VRC 4404 are designed to express clade B HIV-1 Gag, Pol, or Nef, respectively. VRC 5736, VRC 5737, and VRC 5738 are designed to express HIV-1 Env glycoprotein from clade A, clade B, and clade C, respectively. The DNA plasmids have been modified to reduce toxicity.

The vaccine is supplied as a 2 mL glass vial containing a clear colorless isotonic sterile solution. Each vial contains 20% (mg) over the amount to be injected of each plasmid as shown in the table below. Each vial also contains GMP grade phosphate buffered saline (PBS). Each vaccine vial contains 1.2 mL as shown in the table below:

Dose/mL	VRC 4401	VRC 4409	VRC 4404	VRC 5736	VRC 5737	VRC 5738	VRC-HIVDNA016-00-VP (mixture in study vials)
4 mg/mL	0.8 mg	0.8 mg	0.8 mg	0.8 mg	0.8 mg	0.8 mg	4.8 mg (1.2 mL)

The vaccine will be shipped to the study pharmacist on dry ice and stored at -20° C or below until use. Vials of vaccine will be removed from the freezer and allowed to equilibrate to room temperature prior to injection.

One 1 mL injection of the 4 mg/mL preparation will be administered for each 4 mg dose.

Vials may be stored for the duration of the study (not to exceed 2 years) at -20°C or below. Vials should not be refrozen after thawing. VRC-HIVDNA016-00-VP will be tested for stability according to ICH Guidelines. Similar plasmids have been shown to be stable for a minimum of 24 months when stored at -30°C ± 10°C and for up to 24 hours when stored at room temperature. Vials are intended for single use only.

7.1.2 Adenoviral Vector Vaccine, VRC-HIVADV014-00-VP

The recombinant adenoviral vector product VRC-HIVADV014-00-VP (rAd) is manufactured by GenVec, Inc (Gaithersburg, MD). It is produced under cGMP conditions by a contractor, Molecular Medicine (San Diego, CA). The rAd vaccine contains four recombinant serotype 5 adenoviral vectors. These vectors contain gene sequences that encode for clade B HIV-1 Gag and Pol as well as clade A, clade B, and clade C Env protein. *In vivo* expression by these vectors produces immunogens that induce an immune response against HIV. The envelope genes were chosen as representative primary isolates from each of the three clades.

Single use vials will be sent unblinded to the NIH Clinical Center pharmacy. The vial label notes a storage temperature of -10° C to -25° C. The product may be stored in a freezer that has temperatures as low as -30°C. However, if deviations in storage temperature below -30°C or above -10° C occur, the site pharmacist must report the storage temperature deviation promptly to the IND sponsor. The product is shipped on dry ice, during which the product temperature is maintained at ≤ -60°C. Prior to shipping, the vials are sealed in Mylar bags to prevent CO_2 from inactivating the adenoviral vector product. The product vials should not be kept on dry ice without this protection. Once the VRC-HIVADV014-00-VP product is received at the clinical site it should be removed promptly from the dry ice package and the Mylar bag and stored as noted above.

The investigational vaccine vials will be provided at two different concentrations: 1×10^{10} PU/mL or 1×10^{11} PU/mL and each vaccine vial will contain 1.2 mL/vial. Vials may be stored for the duration of the study (not to exceed two years).

The lot release form notes the number of particle units (PU) in the final product. The clinical protocols specify the dose in particle units (PU). Particle units are the number of viral particles, active or not, found in the product as determined by spectrophotometry. Particle units, rather than plaque forming units (pfu) or fluorescent forming units (ffu), are used to determine dose because of the potential toxicity and host immune response to the viral particle, regardless of its ability to infect the target cells. It is also a more accurate measure than either pfu or ffu, which

are highly dependent on methodology, and thus, more variable. Furthermore, the FDA and the Recombinant DNA Advisory Committee have recommended the use of "PU".

7.2 STUDY AGENT ADMINISTRATION

All study injections will be administered into the deltoid muscle. Each study injection must be administered within 4 hours after removing the vaccine vial from the freezer.

7.2.1 Administration of VRC-HIVDNA016-00-VP by Biojector

The Biojector 2000 Needle-Free Injection Management System will be used as directed by the company. Neither the material being injected nor the deltoid injection site skin preparation require deviation from standard procedures. The injection site is disinfected and the area allowed to dry completely. The skin around the injection site is held firmly while the syringe is placed against the injection site at a 90° angle. The actuator is pressed and the material is released into the muscle. Continue to hold firmly for 3 seconds. After the injection, the site is covered with a sterile covering and pressure applied with 3 fingers for 1 minute. Biojector utilizes sterile, single-use syringes for variable dose, up to 1.0 mL, medication administration. The study agent is delivered under pressure by a compressed CO_2 gas cartridge that is stored inside the Biojector. When the Biojector's actuator is depressed, CO_2 is released, causing the plunger to push the study agent out of the sterile syringe through the skin and into the underlying tissue. The study agent is expelled through a micro-orifice at high velocity in a fraction of a second to pierce the skin. The CO_2 does not come in contact with the injectate and the syringe design prevents any back splatter or contamination of the device by tissue from the subject.

Testing of the Biojector for administration of vaccines has demonstrated effective immune responses and although its use is associated with some self-limited pain, redness or swelling at the injection site, this method of administration is well tolerated, and offers the advantage of eliminating needle stick accidents in the clinic [11]. This system has FDA clearance for delivering intramuscular injections of vaccine.

7.2.2 Administration of VRC-HIVDNA016-00-VP by Needle and Syringe

Individual syringes will be prepared by the pharmacy and labeled with the subject identifier for transport to the clinic. The clinician administering the injection will select a 21-gauge needle, with a length of 1 or 1.5 inch (depending on subject arm size) in order to ensure intramuscular injection.

Each used needle and syringe is disposed of in the medical waste sharps container and the sharps container is disposed of in the medical pathology waste (MPW) container for incineration.

7.2.3 Administration of VRC-HIVADV014-00-VP by Needle and Syringe

In accordance with the subjects study agent assignment, a 1 mL volume will be withdrawn from the appropriate vial type for administration. The plan for injection administration is to use standard intramuscular injection technique as follows:

- Administer one 1 mL injection of the 1 x 10^{10} PU/mL preparation for each 10^{10} PU dose;
- Administer one 1 mL injection of the 1 x 10^{11} PU/mL preparation for each 10^{11} PU dose.

Individual syringes will be prepared by the pharmacy and labeled with the subject identifier for transport to the clinic. The clinician administering the injection will select a 21-gauge needle, with a length of 1 or 1.5 inch (depending on subject arm size) in order to ensure intramuscular injection.

7.3 STUDY AGENT LABELING

Vials will be individually labeled with the name of the material, dose, pH, volume, lot number, concentration, storage instructions, Investigational Use Statement ("Caution: New Drug – Limited by Federal Law to Investigational Use"), and manufacturer information. If necessary, additional lots of vaccine will be produced.

7.4 PROCEDURES TO PRESERVE BLINDING

Subjects will be randomized to receive rAd vaccine at 10^{10} PU or 10^{11} PU. The subjects, the clinical staff, and the Principal Investigator will be blinded to treatment allocation until all the safety and immunogenicity evaluations through 6 weeks after the rAd injection are completed. The pharmacist with primary responsibility for drug dispensing keeps the randomization code provided by the protocol statistician.

The study pharmacist will be responsible for preparing the syringe with the vaccine dose indicated by the subject's randomization assignment and labeling it with the subject identification. The pharmacist will not be the same individual who is responsible for clinical follow-up.

During conduct of the study the rAd dosage is blinded until all subjects have completed six weeks of follow-up. The blinding of rAd dosage will be broken early only if, in the opinion of the Principal Investigator, immediate unblinding of a subject's dosage assignment is necessitated by an acute safety concern where knowing the dosage received would change the type of medical care needed to best treat the subject's health problem. The PI would request that the Pharmacy provide the rAd dosage information. The PI would promptly notify the DAIDS Medical Officer, Protocol Statistician and the IRB that the subject's dosage assignment had been unblinded earlier than specified by the protocol.

7.5 STUDY AGENT ACCOUNTABILITY

7.5.1 Documentation

The study pharmacist will be responsible for maintaining an accurate record of the codes, inventory, and an accountability record of vaccine supplies for this study. Electronic documentation as well as paper copies will be used.

7.5.2 Disposition

The empty vials and the unused portion of a vial will be discarded in a biohazard containment bag and incinerated or autoclaved. Any unopened vials that remain at the end of the study will be returned to the production facility or discarded at the discretion of the sponsor in accordance with policies that apply to investigational agents. Partially used vials will not be administered to other subjects or used for *in vitro* experimental studies. They will be disposed of in accordance with institutional or pharmacy policy.

8. HUMAN SUBJECT PROTECTIONS AND ETHICAL OBLIGATIONS

8.1 INFORMED CONSENT

The study informed consent is provided in Appendix I. It describes the investigational product to be used and all aspects involved in protocol participation.

Before a subject's participation in the study, it is the investigator's responsibility to obtain written informed consent from the subject, after adequate explanation of the aims, methods, anticipated benefits, and potential hazards of the study and before any protocol-specific procedures or study medications are administered.

The acquisition of informed consent will be documented in the subject's medical records, as required by 21 CFR 312.62. The informed consent form will be signed and personally dated by the subject and the person who conducted the informed consent discussion. The original signed informed consent form will be retained in the medical chart and a copy will be provided to the subject.

8.2 RISKS AND BENEFITS

8.2.1 Risks

VRC-HIVDNA016-00-VP: The risks noted for the DNA vaccine, VRC-HIVDNA016-00-VP, are based on risks of injections, risks of vaccines in general and interim results of a previous Phase 1 study with this vaccine, as well as other investigational HIV-1 DNA vaccines.

Potential side effects resulting from intramuscular injection include stinging, arm discomfort, or redness of the skin at vaccine injection sites. Subjects may exhibit general signs and symptoms associated with administration of a vaccine injection, including fever, chills, rash, aches and pains, nausea, headache, dizziness and fatigue. These side effects will be monitored, but are generally short term and do not require treatment. Study subjects may self administer medications such as acetaminophen, nonsteroidal anti-inflammatory drugs (NSAIDs), or antihistamines as required. Glucocorticoids will not be used in these study subjects; if such medication is required the study subject will receive no further immunizations, but continue to be monitored in follow-up visits.

Potential risks of DNA vaccines include: muscle damage, antibodies to DNA, insertion of the vaccine DNA into genomic DNA (a potential cancer risk), or insertion of the vaccine DNA into a bacteria or virus. Although these risks are possible, they have not been observed to date in laboratory, animal or human testing of DNA plasmid vaccines.

The most extensive human experience with a multiclade DNA vaccine study is with VRC-HIVDNA009-00-VP. In study VRC 004 (03-I-0022) 40 of the 50 subjects enrolled received vaccine and 10 received placebo. This randomized study was unblinded in September 2004. Both placebo and vaccine recipients were noted to have occasional asymptomatic and self-limited changes in glucose, bilirubin, liver enzymes and urine protein. In the vaccine groups, there were three reportable adverse events possibly related to vaccine. These were a grade 3 asymptomatic neutropenia seen on test results from 27 days after 3rd vaccination (4 mg group) that was normal on repeat testing 5 days later, a grade 3 urticaria with onset 4 days after 3rd vaccination (4 mg group) and a grade 2 maculopapular rash with onset 27 days after 2nd vaccination (8 mg group). All resolved without sequelae. Other factors in the occurrence of the

urticaria include concomitant bladder infection, yeast infection and multiple antibiotics. The rash resulted in discontinuation from the vaccination schedule after the 2nd injection and it was clinically consistent with either a drug eruption or a viral exanthem. Informed consents for protocols using similar DNA vaccines should note the potential risk of neutropenia, rash and hives. Extramural randomized, blinded studies with this vaccine are ongoing.

The multiclade DNA vaccine that will be used in this study is VRC-HIVDNA016-00-VP, which has been administered to 15 subjects in the open-label study VRC 007 (04-I-0254). No subject reported fever following vaccination. Reactogenicity was none to mild except that two subjects reported moderate injection site pain and one subject reported moderate nausea and malaise. There has been one grade 3 adverse event (chronic urticaria possibly related to vaccination) requiring expedited reporting to the IND sponsor. To date, there have been two moderate (grade 2) adverse events possibly attributed to vaccine. These were intermittent dizziness of 2 days duration beginning 13 days after the second vaccination in one subject (this subject received the third vaccination without recurrence of symptoms) and asymptomatic hypoglycemia in another subject, first noted at the follow-up visit that was 14 days after the third vaccination. Four of the 44 injections were associated with mild skin lesions (0.5-1.0 cm diameter) at the vaccination site. A small scab formed within a week after immunization and came off after a few days. The skin healed without treatment within a few weeks. One skin biopsy was obtained on day 6 post vaccination. It showed subcutaneous and dermal perivascular lymphocytic inflammation. There were rare eosinophils and rare giant cells noted, and the infiltrate was composed entirely of CD3 positive cells. It included both $CD4^+$ and $CD8^+$. The process appears to be primarily a subcutaneous inflammatory response to vaccination with cutaneous manifestations. There have been no serious adverse events to date.

VRC-HIVADV014-00-VP: There is limited human experience with the adenoviral vector vaccine, VRC-HIVADV014-00-VP. It has previously been administered at the NIH Clinical Center in the first Phase I study in humans. The first extramural study using this vaccine opened in November 2004. At the 10^{10} PU dose none of the subjects in the first Phase I study had fever and the other reactogenicity was mild or none. At the next higher dose (10^{11} PU) four subjects had a flu-like set of symptoms with fever, headache, muscle aches, malaise and chills starting 12-16 hours after vaccination and lasting a few hours. Some of these symptoms were moderate in severity. A few subjects have had nausea. Some subjects have had injection site pain or discomfort in the first few days after a vaccination. These symptoms improved after treatment with over-the-counter medicine.

One subject with a history of intermittent low neutrophil count, had a neutrophil count that was moderately below normal shortly after the study vaccination. This returned to normal without any symptoms of illness. A different subject was noted to have persistent grade 1 ALT starting 25 days after study injection that lasted for about 5 months. An evaluation diagnosed fatty liver (steatohepatitis). There are no clinical symptoms. The condition may have existed prior to study enrollment and the subject's alcohol use and recent weight gain may be contributing factors. One subject with a history of a single seizure three years prior to study enrollment, experienced a seizure 64 days after study injection. This subject now has a diagnosis of epilepsy and is on anticonvulsant therapy. Following review of the subject's medical records and given the timing of the seizure, the seizure was assessed as unrelated to study vaccine. Other subjects have had mild temporary changes in blood or urine tests. It is unknown whether the lab test changes, diarrhea, fatty liver, or seizure were due to vaccine or to other factors or to a combination of the

vaccine with other factors.

In preclinical testing in rabbits, this investigational vaccine was associated with fever the day after injection and decreased food consumption 1-2 days after injection. After a second injection, inflammation in the muscle around the injection site was noted, including tissue around capillaries and lymphatics near the sciatic nerve; however no nerve damage was noted. Transient and asymptomatic changes in cholesterol and triglycerides were not associated with clinical pathology and transient increase in creatine phosphokinase (CPK) was possibly related to the muscle inflammation.

The effect of the study vaccines on a fetus or nursing baby is unknown, so female subjects of child bearing potential will be required to agree to use birth control for sexual intercourse beginning 21 days prior to enrollment and continuing through Week 42. Women who are pregnant or nursing will be excluded from the study.

Either vaccine may cause a positive HIV antibody test using the standard screening test. A positive or indeterminate test may have a negative employment and social impact. Western blot analysis and HIV PCR or other testing will be done to either exclude or confirm HIV infection. ELISA, Western Blot, and PCR results will be discussed with the study subject as they become available.

Blood drawing may cause pain, bruising, and, rarely, infection at the site where the blood is taken.

Subjects may believe that this vaccine provides protection, and therefore practice riskier behavior. They will receive extensive counseling throughout the study to address this potential problem.

8.2.2 Benefits

It is unknown if any benefit will result from study participation. Others may benefit from knowledge gained in this study that may aid in the development of an HIV vaccine.

8.3 INSTITUTIONAL REVIEW BOARD

A copy of the protocol, proposed informed consent form, other written subject information, and any proposed advertising material will be submitted to the IRB for written approval.

The investigator must submit and, where necessary, obtain approval from the IRB for all subsequent protocol amendments and changes to the informed consent document. The investigator will notify the IRB of deviations from the protocol and serious adverse events.

The investigator will be responsible for obtaining IRB approval of the annual Continuing Review throughout the duration of the study.

8.4 PROTOCOL REGISTRATION

The Division of AIDS, NIAID is the IND sponsor for this protocol. Protocol registration must occur before subjects are enrolled in this study. The Institutional Review Board (IRB) must approve the protocol and consent form. The protocol must be submitted to the Institutional Biosafety Committee (IBC). Approval letters from both the IRB and IBC must be submitted to the Division of AIDS Regulatory Compliance Center (RCC) Protocol Registration Office with

the initial protocol registration. Subsequent protocol amendments must also be registered with and approved by the RCC Protocol Registration Office.

8.5 SUBJECT CONFIDENTIALITY

The investigator must ensure that the subject's anonymity is maintained. Subjects will not be identified in any reports on this study. All records will be kept confidential to the extent provided by federal, state and local law. Medical records are made available for review when required by the Food and Drug Administration or other authorized users, such as the vaccine manufacturer, only under the guidelines set by the Federal Privacy Act. Direct access includes examining, analyzing, verifying, and reproducing any records and reports that are important to the evaluation of the study. The investigator is obligated to inform the subjects that the above named representatives will review their study-related records without violating the confidentiality of the subjects.

8.6 PLAN FOR USE AND STORAGE OF BIOLOGICAL SAMPLES

Stored study research samples are labeled by a code (such as a number) that only the VRC Clinic team can link to the subject. All stored research samples are logged into the VRC Laboratory Information Management System (LIMS) database and uses of these samples are documented in the LIMS. The requirement to maintain subject confidentiality is included in the study informed consent document.

8.7 SUBJECT IDENTIFICATION AND ENROLLMENT OF STUDY PARTICIPANTS

All study activities will be carried out at the Clinical Center at the National Institutes of Health. Study subjects will be recruited through on-site and off-site advertising done for the screening protocol, VRC 000 (02-I-0127). Effort will be made to include women and minorities in proportions similar to that of the community from which they are recruited. Because this Phase I study is designed to establish safety of the vaccine in healthy adults, enrollment will be limited to persons at least 18 years of age, and no older than 50 years of age.

8.7.1 Participation of Children

Children are not eligible to participate in this clinical trial because it does not meet the guidelines for inclusion of children in research. These guidelines (45 CFR 46, Subpart D, 401-409), state the Department of Health and Human Services protections for children who participate in research. Generally, healthy children can be studied when the research is considered as "not greater than minimal risk." Children can be involved in research with greater than minimal risk only when it presents the prospect of direct benefit to the individual child or is likely to yield generalizable knowledge about the child's disorder or condition.

8.8 COMPENSATION

Subjects will be compensated for time and inconvenience in accordance with the standards for compensation of the Clinical Research Volunteer Program. The compensation per visit will be $100 for visits that include injections and blood drawing, $70 for visits that include blood drawing but no injection, and $30 for visits that include injection site inspection and photo (including removal of skin biopsy suture, if needed). The approximate total compensation for the subject will be between $1050 and $1140, based on the projected 15-18 clinic visits and four

injections through Week 42. Subjects who consent to a skin biopsy will be compensated $70 per skin biopsy. Those who return at Week 94 for a long-term follow-up blood draw will be compensated $70 for that visit.

8.9 Safety Monitoring

Close cooperation between the designated members of the Protocol Team will occur to evaluate and respond to individual adverse events in a timely manner. Designated team members (Principal Investigator, Medical Officer, Protocol Specialist, Study Coordinator and other study clinicians) will review the summary study safety data reports on a weekly basis through 4 weeks after the last subject receives the third study injection in order to be certain that the vaccine has an acceptable safety profile and will continue to monitor the study safety data reports on a monthly basis through completion of the last Week 42 visit. The DAIDS Medical Officer will provide an independent review of adverse events that have a bearing on study stopping. A quarterly safety monitoring report will be provided to the FDA as requested during a conference call on November 9, 2005 (see Section 4.7).

9. ADMINISTRATIVE AND LEGAL OBLIGATIONS

9.1 Protocol Amendments and Study Termination

Protocol Amendments must be made only with the prior approval of the National Institute of Allergy and Infectious Diseases' Division of AIDS and Vaccine Research Center. Agreement from the investigator must be obtained for all protocol amendments and amendments to the informed consent document. All study amendments will be submitted to the IRB for approval.

The Division of AIDS, National Institute of Allergy and Infectious Diseases, the Vaccine Research Center, the Principal Investigator and the Food and Drug Administration reserve the right to terminate the study. The investigator will notify the IRB in writing of the study's completion or early termination.

9.2 Study Documentation and Storage

The investigator will maintain a list of appropriately qualified persons to whom trial duties have been delegated.

Source documents are original documents, data, and records from which the subject's data are obtained. These include but are not limited to hospital records, clinical and office charts, laboratory and pharmacy records, diaries, microfiches, radiographs, and correspondence.

The investigator and staff are responsible for maintaining a comprehensive and centralized filing system of all study-related (essential) documentation, suitable for inspection at any time by representatives from the National Institute of Allergy and Infectious Diseases' Division of AIDS and Vaccine Research Center, IRB, FDA, and/or applicable regulatory authorities. Elements include:

- Subject files containing completed informed consent forms, and supporting copies of source documentation (if kept)
- Study files containing the protocol with all amendments, Investigator Brochures, copies of all correspondence with the IRB and the National Institute of Allergy and Infectious Diseases' Division of AIDS and Vaccine Research Center

In addition, all original source documentation must be maintained and be readily available.

All essential documentation should be retained by the institution for the same period of time required for medical records retention. The FDA requires study records to be retained for up to two years after marketing approval or refusal (21 CFR 312.62). No study document should be destroyed without prior written agreement between the National Institute of Allergy and Infectious Diseases' Division of AIDS and Vaccine Research Center and the investigator. Should the investigator wish to assign the study records to another party or move them to another location, they must notify the National Institute of Allergy and Infectious Diseases' Division of AIDS and Vaccine Research Center in writing of the new responsible person and/or the new location.

9.3 STUDY MONITORING, DATA COLLECTION AND DATA MONITORING

9.3.1 Study Monitoring

The National Institute of Allergy and Infectious Diseases' Division of AIDS and Vaccine Research Center regulatory authority inspectors or their authorized representatives are responsible for contacting and visiting the investigator for the purpose of inspecting the facilities and, upon request, inspecting the various records of the trial, provided that subject confidentiality is respected.

Site visits by study monitors will be made in accordance with the IND Sponsor (DAIDS) policy to monitor the following: study operations, the quality of data collected in the research records, the accuracy and timeliness of data entered in the database, and to determine that all process and regulatory requirements are met.

Site investigators will allow the study monitors, the NIAID IRB, and the FDA to inspect study documents (e.g., consent forms, drug distribution forms, case report forms) and pertinent hospital or clinic records for confirmation of the study data.

9.3.2 Data Collection

Clinical research data will be collected in a secure electronic data management system through a contract research organization, EMMES (Rockville, MD). Extracted data without patient identifiers will be sent to the Protocol Statistician for statistical analysis.

9.4 LANGUAGE

All written information and other material to be used by subjects and investigative staff must use vocabulary and language that are clearly understood.

9.5 POLICY REGARDING RESEARCH-RELATED INJURIES

The Clinical Center will provide short-term medical care for any injury resulting from participation in this research. In general, the National Institutes of Health, the Clinical Center, or the Federal Government will provide no long-term medical care or financial compensation for research-related injuries.

10. REFERENCES

1. CDC, *Increases in HIV diagnoses--29 States, 1999-2002.* MMWR Morb Mortal Wkly Rep, 2003. **52**(47): p. 1145-8.

2. UNAIDS, *AIDS Epidemic Update December 2003.* 2003.

3. WHO, *Treating 3 Million by 2005: The WHO Strategy*. 2003, World Health Organization: Geneva, Switzerland. p. 1-53.

4. *Approaches to the development of broadly protective HIV vaccines: challenges posed by the genetic, biological and antigenic variability of HIV-1: Report from a meeting of the WHO-UNAIDS Vaccine Advisory Committee Geneva, 21-23 February 2000.* AIDS, 2001. **15**(6): p. W1-W25.

5. Nabel, G., W. Makgoba, and J. Esparza, *HIV-1 Diversity and Vaccine Development.* Science, 2002. **296**(5577): p. 2335.

6. Osmanov, S., et al., *Estimated global distribution and regional spread of HIV-1 genetic subtypes in the year 2000.* J Acquir Immune Defic Syndr, 2002. **29**(2): p. 184-90.

7. Amara, R.R., et al., *Control of a mucosal challenge and prevention of AIDS by a multiprotein DNA/MVA vaccine.* Science, 2001. **292**(5514): p. 69-74.

8. Shiver, J.W., et al., *Replication-incompetent adenoviral vaccine vector elicits effective anti-immunodeficiency-virus immunity.* Nature, 2002. **415**(6869): p. 331-5.

9. Casimiro, D.R., et al., *Comparative immunogenicity in rhesus monkeys of DNA plasmid, recombinant vaccinia virus, and replication-defective adenovirus vectors expressing a human immunodeficiency virus type 1 gag gene.* J Virol, 2003. **77**(11): p. 6305-13.

10. Sullivan, N.J., et al., *Development of a preventive vaccine for Ebola virus infection in primates.* Nature, 2000. **408**(6812): p. 605-9.

11. Jackson, L.A., et al., *Safety and immunogenicity of varying dosages of trivalent inactivated influenza vaccine administered by needle-free jet injectors.* Vaccine, 2001. **19**(32): p. 4703-9.

12. Simonsen, L., et al., *Unsafe injections in the developing world and transmission of bloodborne pathogens: a review.* Bull World Health Organ, 1999. **77**(10): p. 789-800.

13. Levine, M.M., *Can needle-free administration of vaccines become the norm in global immunization?* Nat Med, 2003. **9**(1): p. 99-103.

14. Mathei, C., P. Van Damme, and A. Meheus, *Hepatitis B vaccine administration: comparison between jet-gun and syringe and needle.* Vaccine, 1997. **15**(4): p. 402-4.

15. Williams, J., et al., *Hepatitis A vaccine administration: comparison between jet-injector and needle injection.* Vaccine, 2000. **18**(18): p. 1939-43.

16. Epstein, J.E., et al., *Safety, tolerability, and lack of antibody responses after administration of a PfCSP DNA malaria vaccine via needle or needle-free jet injection, and comparison of intramuscular and combination intramuscular/intradermal routes.* Hum Gene Ther, 2002. **13**(13): p. 1551-60.

17. Fisch, A., et al., *Immunogenicity and safety of a new inactivated hepatitis A vaccine: a clinical trial with comparison of administration route.* Vaccine, 1996. **14**(12): p. 1132-6.

18. Manam, S., et al., *Plasmid DNA vaccines: tissue distribution and effects of DNA sequence, adjuvants and delivery method on integration into host DNA.* Intervirology, 2000. **43**(4-6): p. 273-81.

19. Mumper, R.J. and Z. Cui, *Genetic immunization by jet injection of targeted pDNA-coated nanoparticles.* Methods, 2003. **31**(3): p. 255-62.

20. Aguiar, J.C., et al., *Enhancement of the immune response in rabbits to a malaria DNA vaccine by immunization with a needle-free jet device.* Vaccine, 2001. **20**(1-2): p. 275-80.

21. Wang, R., et al., *Induction of CD4(+) T cell-dependent CD8(+) type 1 responses in humans by a malaria DNA vaccine.* Proc Natl Acad Sci U S A, 2001. **98**(19): p. 10817-22.

22. Piedra, P.A., et al., *Incidence and prevalence of neutralizing antibodies to the common adenoviruses in children with cystic fibrosis: implication for gene therapy with adenovirus vectors.* Pediatrics, 1998. **101**(6): p. 1013-9.

23. Horwitz, M.S., *Adenoviruses*, in *Fields Virology*, D.M.K.a.P.M. Howley, Editor. 2001, Lippincott Williams and Wilkins: Philadelphia. p. 2301-2326.

24. Pacini, D.L., A.M. Collier, and F.W. Henderson, *Adenovirus infections and respiratory illnesses in children in group day care.* J Infect Dis, 1987. **156**(6): p. 920-7.

25. D'Ambrosio, E., et al., *Neutralizing antibodies against 33 human adenoviruses in normal children in Rome.* J Hyg (Lond), 1982. **89**(1): p. 155-61.

26. Chirmule, N., et al., *Immune responses to adenovirus and adeno-associated virus in humans.* Gene Ther, 1999. **6**(9): p. 1574-83.

27. Kostense, S., et al., *Adenovirus types 5 and 35 seroprevalence in AIDS risk groups supports type 35 as a vaccine vector.* Aids, 2004. **18**(8): p. 1213-6.

28. Vogels, R., et al., *Replication-deficient human adenovirus type 35 vectors for gene transfer and vaccination: efficient human cell infection and bypass of preexisting adenovirus immunity.* J Virol, 2003. **77**(15): p. 8263-71.

29. Emini, E.A., *A potential HIV-1 vaccine using a replication-defective adenoviral vaccine vector*, in *9th Conference of Retroviruses and Opportunistic Infections*. 2002: Seattle, Washington.

30. Isaacs, R. *Impact of pre-existing immunity on the immunogenicity of adenovirus serotype 5-based vaccines*. in *AIDS Vaccine 2004*. 2004. Lausanne, Switzerland.

31. Aste-Amezaga, M., et al., *Quantitative adenovirus neutralization assays based on the secreted alkaline phosphatase reporter gene: application in epidemiologic studies and in the design of adenovector vaccines.* Hum Gene Ther, 2004. **15**(3): p. 293-304.

32. Sprangers, M.C., et al., *Quantifying adenovirus-neutralizing antibodies by luciferase transgene detection: addressing preexisting immunity to vaccine and gene therapy vectors.* J Clin Microbiol, 2003. **41**(11): p. 5046-52.

33. Betts, M.R., J.P. Casazza, and R.A. Koup, *Monitoring HIV-specific CD8+ T cell responses by intracellular cytokine production.* Immunol Lett, 2001. **79**(1-2): p. 117-25.

34. Helms, T., et al., *Direct visualization of cytokine-producing recall antigen-specific CD4 memory T cells in healthy individuals and HIV patients.* J Immunol, 2000. **164**(7): p. 3723-32.

35. Malenbaum, S.E., D. Yang, and C. Cheng-Mayer, *Evidence for similar recognition of the conserved neutralization epitopes of human immunodeficiency virus type 1 envelope gp120 in humans and macaques.* J Virol, 2001. **75**(19): p. 9287-96.

36. Mascola, J.R., et al., *Human immunodeficiency virus type 1 neutralization measured by flow cytometric quantitation of single-round infection of primary human T cells.* J Virol, 2002. **76**(10): p. 4810-21.

37. Peng, B. and M. Robert-Guroff, *Deletion of N-terminal myristoylation site of HIV Nef abrogates both MHC-1 and CD4 down-regulation.* Immunol Lett, 2001. **78**(3): p. 195-200.

38. Liang, X., et al., *Development of HIV-1 Nef vaccine components: immunogenicity study of Nef mutants lacking myristoylation and dileucine motif in mice.* Vaccine, 2002. **20**(27-28): p. 3413-21.

39. Chakrabarti, B.K., et al., *Modifications of human immunodeficiency virus envelope glycoprotein enhance immunogenicity for genetic immunization.* J Virol, 2002. **76**(11): p. 5357-5368.

40. Seth, P., *Adenoviruses: basic biology to gene therapy*. Medical intelligence unit. 1999, Austin, TX: R.G. Landes Co.

41. Brough, D.E., et al., *A gene transfer vector-cell line system for complete functional complementation of adenovirus early regions E1 and E4.* J Virol, 1996. **70**(9): p. 6497-501.

42. Rasmussen, H., et al., *TNFerade Biologic: preclinical toxicology of a novel adenovector with a radiation-inducible promoter, carrying the human tumor necrosis factor alpha gene.* Cancer Gene Ther, 2002. **9**(11): p. 951-7.

APPENDIX I
STUDY INFORMED CONSENT FORM

INSTITUTE: Vaccine Research Center, National Institute of Allergy and Infectious Diseases

STUDY NUMBER: 05-I-0148 PRINCIPAL INVESTIGATOR: Andrew Catanzaro, M.D.

STUDY TITLE: VRC 008: A Phase I Clinical Trial of a Prime-Boost HIV-1Vaccination Schedule: Multiclade DNA Vaccine, VRC-HIVDNA016-00-VP, Followed by Multiclade Adenoviral Vector Vaccine, VRC-HIVADV014-00-VP, in Uninfected Adult Volunteers

Latest IRB Review:
Latest Amendment Approved:

Study Consent

INTRODUCTION

We invite you to take part in a research study at the National Institutes of Health (NIH).

First, we want you to know that:

> Taking part in NIH research is entirely voluntary.
>
> You may choose not to take part, or you may withdraw from the study at any time. In either case, you will not lose any benefits to which you are otherwise entitled. However, to receive care at the NIH, you must be taking part in a study or be under evaluation for study participation.
>
> You may receive no benefit from taking part. The research may give us knowledge that may help people in the future.

Second, some people have personal, religious or ethical beliefs that may limit the kinds of medical or research treatments they would want to receive (such as blood transfusions). If you have such beliefs, please discuss them with your NIH doctors or research team before you agree to the study.

Now we will describe this research study. Before you decide to take part, please take as much time as you need to ask any questions and discuss this study with anyone at NIH, or with family, friends or your personal physician or other health professional.

PURPOSE OF THE STUDY

The main purpose of this study is to see if a vaccination schedule in which all study participants receive two different kinds of experimental HIV vaccines is safe and whether it causes any side effects. The schedule includes 3 injections (shots) with one vaccine and 1 injection with another vaccine. It is called a prime-boost schedule. Other goals of the study are:

- To see whether the experimental vaccines cause an immune response.
- To see if injection of the first vaccinations using a needle and syringe is similar in safety and immune response to giving the injections with a needleless injection device.

- To see if people who already have antibodies to adenovirus (part of the second vaccine product) still have an immune response to the second vaccine.
- To monitor the social impact of being in an HIV vaccine study. Social harms are such things as problems with insurance, health care, friends, family, employment, housing, education, or government agencies.

The two experimental HIV vaccines in this study are known as VRC-HIVDNA016-00-VP and VRC-HIVADV014-00-VP. We call the first one "the DNA vaccine" and the second one the "rAd vaccine." The experimental vaccines have been given to people before, but only in research studies. This is one of the first two studies that will give both vaccines to people in a "prime-boost" schedule. "Experimental" means that the study vaccines have not been approved by the Food and Drug Administration (FDA) for treating or preventing HIV infection. The FDA allows them to be used in research studies only. It is not known if the study vaccines prevent HIV infection.

You are eligible to participate in this study because you have completed the screening process, completed an assessment of understanding, are HIV-negative, are between 18 and 50 years old, do not have any significant medical problems and you are willing to donate blood samples for future research.

Forty (40) people will participate in this study at the NIH Clinical Center in Bethesda, Maryland. The study visits will take about 42 weeks to complete. There is one long-term follow-up at Week 94. While on the study, you will be checked for vaccine side effects. You will be treated at the National Institutes of Health if any side effects occur.

You will be told of any new information learned during this study that might cause you to change your mind about staying in the study. At the end of the study, you will be told when study results may be available and how to learn about them.

STUDY VACCINES

Vaccines are substances used to try to create an immune response (the body's natural defenses) to prevent or resist an infection. Many vaccines are made of proteins and injected into a muscle. Proteins are natural substances that the body uses as building blocks.

There is no live HIV virus in the study vaccines. You cannot get HIV infection or AIDS from the study vaccines or from the proteins that may be made from the vaccines.

The DNA vaccine is the first type of vaccine you will get. The DNA provides the code (instructions) that allows cells to make proteins. The DNA in this vaccine was made in a laboratory using synthetic DNA. It codes for parts of four HIV proteins, known as Gag, Pol, Nef and Env. The DNA will instruct your muscle cells to make these parts of HIV protein. The investigators will do blood tests to see if the body has an immune response to the vaccine proteins.

The rAd vaccine is the second type of vaccine you will get. It is a modified adenovirus that will carry DNA into your muscle cells. The rAd vaccine has codes for parts of the three HIV proteins called Gag, Pol, and Env. The manufactured DNA has been packaged in an adenovirus shell that is missing some of the usual adenovirus genes. The adenovirus used in this vaccine has been changed so that it cannot reproduce in a human body.

Adenovirus is a common virus that causes upper respiratory infections (such as the common cold), eye infection (conjunctivitis), urine infection or diarrhea. You cannot get an adenovirus infection from the rAd vaccine because of the way it has been changed. You cannot infect someone else with the study vaccine adenovirus. An adenovirus with DNA inserted in a laboratory is called a vector or adenoviral vector.

An important feature of the rAd vaccine is that the adenoviral vector allows the muscle cells to make the Gag, Pol and Env proteins for several days. This mimics the way the immune system sees virus proteins during a virus infection.

In order to create the adenoviral vector, cultures derived from human fetal kidney cells were used. The cell culture was started in the 1980s. Production of the rAd vaccine does not require getting new fetal kidney cells. There are not any human cells in the rAd vaccine injection.

The NIH, including some members of the Vaccine Research Center scientific staff, developed the investigational vaccines being used in this research study. The results of this study could play a role in whether the FDA will approve the vaccines for sale at some time in the future. If approved, the future sale of the vaccines could lead to payments to the NIH and to some NIH/VRC scientists. By U.S. law, government scientists are required to receive such payments for their inventions. You will not receive money or other compensation should this occur. Please discuss with your study doctor any questions you may have about these issues.

STUDY PROCEDURES

In the study you will receive three injections of a 4 milligram (mg) dose of the DNA vaccine. The injections (shots) will be given into muscle in your upper arms. You may be injected with the DNA vaccine with either a needleless injection device or with the commonly used needle and syringe. You will get one injection of the rAd vaccine in the upper arm with a needle and syringe. You may get a dose of 10^{10} PU or a dose of 10^{11} PU of the rAd vaccine. A PU is a “particle unit.” If you get 10^{10} PU of the rAd vaccine it means you will be getting ten billion (10,000,000,000) units. The injection schedule is shown in the table below.

**Number of People**	**Day 0**	**Week 4 (±7 days)**	**Week 8 (±7 days)**	**Week 24 (-7 to +14 days)**
40	DNA vaccine	DNA vaccine	DNA vaccine	rAd vaccine
	(at least 21 days between DNA vaccine injections)			

If you enroll, you will be given a study number. The study will have 40 subjects. The study will enroll 20 people with high levels of adenovirus antibodies and 20 people with low levels of

adenovirus antibodies. There are 40 study numbers; half of the study numbers are reserved for each group of 20. People in both groups may enroll at the same time. Your antibody level for adenovirus (type 5) was measured when you were screened. Whether you have high or low levels depends on if you had an adenovirus (type 5) infection in the past, how long ago it was, and other factors. To complete your enrollment, a study nurse will enter your adenovirus antibody level and your eligibility information into a computer and it will assign your study number.

Before the study started each study number was randomly assigned (like flipping a coin) to the DNA vaccine injection device and to the rAd vaccine dose that goes with the study number. Whether you have a high or a low adenovirus antibody levels, you have an equal chance of receiving your three DNA injections with a needle and syringe or with a needleless injection device called a "Biojector 2000". You will find out shortly after enrolling whether the needle and syringe or the Biojector will be used for your three DNA vaccine injections. The Biojector is a hand-held system that delivers the vaccine through the skin without the use of a needle. This system has FDA clearance for delivering vaccine injections into muscles.

Whether you have a high or a low adenovirus antibody levels, you also have an equal chance of receiving either the 10^{10} PU rAd dose or the 10^{11} PU rAd dose at Week 24. Neither you nor the study staff will know which dose of rAd vaccine you will get at Week 24. Several weeks after the last person in the study gets the rAd vaccine you and the study staff will be told which dose of the rAd vaccine you received.

You will get your first vaccination on the day you enroll in the study. This is called Day 0. All injections will be given in an upper arm (deltoid) muscle. Your right and left arms will be alternated each time you have an injection. The clinic staff will observe you for at least 30 minutes after each vaccination. You will be asked to complete a diary card at home for 5 days after each vaccination. This will require that you record your temperature and symptoms and look at the injection site each day for 5 days. The diary card must be turned in to the clinic at the first clinic visit that occurs after all 5 days are completed.

After each of the three DNA vaccinations you must come in for a clinic visit about 3 days later. As instructed at that visit, you must either come in or call the clinic about 7 days after the vaccination.

After the rAd vaccination you must call a study nurse one or two days after the vaccination. The telephone call is a required appointment. If you do not contact a study nurse for the required telephone call, a study nurse will call you.

After any injection, you must come to the clinic to be seen if you have a rash, hives, fever of 101.6˚F or higher, or substantial difficulty in daily activities (such as going to work or taking care of yourself). You will also need to come to the clinic for any problem which the nurse or doctor thinks should be checked by exam or blood tests or urine tests. It is very important that you follow the instructions given to you by the clinic staff.

It is important for you to know that in the first study of the experimental DNA vaccine there were 15 subjects and 44 injections were given. After 4 of the 44 injections, a small red bump formed followed by a scab. The scab formed at the injection site within about a week. These healed without treatment within a few weeks. A photo will be taken of your arm before each DNA vaccination. After each DNA vaccination, the study nurse will look carefully at your arm at follow-up visits. The nurse will measure any redness or swelling and look to see if there is a bump, skin changes, or a scab forming. The nurse will take a photograph of your injection site. If your injection site develops a bump or scab, then more photos may be taken on each visit until your skin is healed. Study investigators will use the photographs to learn more about this type of vaccine reaction.

You may be asked to have a skin biopsy of your injection site if a bump or scab is forming. This is for research purposes to better understand how the skin is reacting to the DNA vaccine. You may refuse to have a skin biopsy. You will not be asked to have more than two skin biopsies for research purposes during the study. You may have more skin biopsies if needed for health care purposes. A skin biopsy is a medical procedure for removing a small piece of skin. It is explained in more detail in the skin biopsy consent form. Before a skin biopsy can be done, you will need to sign a separate consent for the skin biopsy procedure on the day of the skin biopsy.

You will have about 15-18 clinic visits over 42 weeks after you enroll in this study. The exact number depends on how many times the clinic needs to see your vaccination site. The four vaccination visits will each take about 4 hours to complete. Clinic visits with a blood collection will usually take about 2 hours. Some visits may be shorter.

At each visit, you will be checked for any health changes or problems. You will be asked how you are feeling and if you have taken any medications. This includes pills, injections, over-the-counter medications and herbal supplements, skin medications, inhalers and any other form of medications. It is important to talk to a study nurse or doctor before getting a flu vaccine or any other vaccine while in this study. The study staff will help you schedule other vaccinations so that they fit in with the study plan and your health needs are met. You may have to stop the study vaccinations if you get certain medicines or get other vaccinations. If it is not an emergency, call a study nurse or doctor before starting new medicines or getting injections of any type.

Urine samples will be collected and blood will be drawn at some study visits for checking on your health. You will be told promptly if any of your test results show a health problem. Some blood samples will be used to study your immune response to the vaccine. Results of immune response tests are not tests used to check on your health and will not be given to you during the study.

The amount of blood drawn will vary from about 1 tablespoon (15 mL) to about 10 tablespoons (150 mL), depending on the visit. You might also be asked to have laboratory tests between regular visits if needed to evaluate a change in your health. The total amount of blood drawn during the 42 weeks of study clinic visits will be about 6 cups (1350 mL). No more than a total of about two cups (450 mL) will be drawn over any six-week period during the study.

You will be tested for HIV several times and asked questions about your sexual behavior and drug use. Throughout the study you will be counseled on HIV risk reduction. You will also be asked about any social effects you may have experienced from your participation in this study. The vaccines used in this study cannot cause HIV infection. If you become infected with HIV during this study for other reasons, you will not receive any additional study injections. You will be referred for medical care. Medical treatment for HIV infection is not part of this study. You will be asked to continue with study follow-up visits through the full 42 weeks of study.

If you have either serious side effects from the vaccine or learn of any new health problems while on the study, the study doctor may decide that you should not receive any more vaccinations. However, you will be asked to continue your study follow-up visits.

The clinic visit schedule for 42 weeks is shown below. You must also call the clinic one or two days after rAd vaccination or any time you have a health problem.

Day or Week of Study	Day 0	Wk 1	Wk 2	Wk 4	Wk 5	Wk 6	Wk 8	Wk 9	Wk 10	Wk 12	Wk 24	Wk 26	Wk 28	Wk 30	Wk 42
Assessment of Understanding and Informed Consent	X														
Vaccinations	X			X			X				X				
Photo of arm	X	X		X	X		X	X							
**Phone call		X			X			X			X				
5-day Diary card	X			X			X				X				
Medical history; If needed, physical exam	X	X	X	X	X	X	X	X	X	X	X	X	X	X	X
Vital signs & weight	X	X	X	X	X	X	X	X	X	X	X	X	X	X	X
Lymph node exam	X	X	X	X	X	X	X	X	X	X	X	X	X		
Blood Samples	X		X	X		X	X		X	X	X	X	X	X	X
Pregnancy Test (for females)	X			X			X				X				X
HIV Testing	X									X	X			X	X
Urine Sample	X		X	X		X	X		X		X	X			
Counseling on HIV Tests, Reducing Risk; Pregnancy (as needed)	X	X	X	X	X	X	X	X	X	X	X	X	X	X	X
Social Impact Questionnaire															X

**** Clinic visit required if any symptoms need to be checked.**

You have a choice of an extra clinic visit or contact by telephone, e-mail or mail to answer questions about your health at Week 94. If you come to the clinic you will be asked to allow blood samples to be collected for research and HIV testing.

MONITORING OF THE STUDY

This study will be monitored by a group of physicians and scientists at the Clinical Center. This group will review the information from the study and will pay close attention to harmful reactions. If it is decided that significant reactions have occurred, further injections may be delayed or canceled.

HIV TESTING

As part of your participation in this study, it will be necessary to test your blood for the presence of antibodies to the Human Immunodeficiency Virus (HIV), the virus that causes Acquired Immune Deficiency Syndrome (AIDS). If antibodies are present, other tests will be done to determine if you have HIV infection. In order to perform the test, a small amount of blood (approximately 2 teaspoons) will be withdrawn from one of your arms with a needle. You may experience some slight discomfort at the needle entry site and there may be some bruising. In addition, there is a very small risk of you fainting or of infection at the needle entry site. If you are diagnosed as having HIV infection, you should be aware of the following Clinical Center HIV Testing Policy:

1. Your physician will notify you promptly of the HIV test results.

2. Your physician and/or the Clinical Center HIV counselor will offer you, and any current and/or ongoing sexual partner(s) (spouses are generally considered to be current or ongoing sexual partners) or needle-sharing partner(s) you identify, information on the meaning of the test results and how to prevent the spread of the infection.

3. Because the virus may be transmitted in several ways, it is important that you inform sexual and/or needle-sharing partner(s) that any, or all, of them may have been exposed to the HIV virus and encourage them to be tested. If you request it, staff at the Clinical Center will assist you in notifying your partner(s) and arrange counseling for them through an HIV counselor.

4. The results of your HIV test and/or documentation of the diagnosis of AIDS will become a part of your Clinical Center medical record and, as such, will be protected from unauthorized disclosure by the Federal Privacy Act of 1974. In general, access to your medical record will be restricted to those health care professionals directly involved in your care or in the conduct of ongoing biomedical research, and information is not usually released to other third parties without your permission or that of your designated representative. However, there are some particular routine uses of such information of which you should be aware.

   (a) If you are unwilling or unable to notify your partner(s), the Clinical Center is responsible for attempting to contact and inform them of their possible exposure to the virus. Reasonable attempts will be made to protect your identity including withholding your name when notifying any partner(s) of their possible exposure. Some notification or counseling of current and/or ongoing partners may be carried out through arrangements with, or referral to, local public health agencies.

(b) The Clinical Center may report certain communicable diseases, such as HIV infection, to appropriate State and Federal government agencies.

i. For Clinical Center patients who are Maryland residents, the Clinical Center reports by "Patient Unique Identifier Number" (rather than by name) newly obtained HIV-positive results from its laboratory to the Maryland Department of Health and Mental Hygiene. Patient Unique Identifier Number is: last four digits of social security number, birth month, birth day, birth year, race, and gender.

ii. For Clinical Center patients who are Maryland residents, the Clinical Center reports by name new cases of AIDS to the Maryland Department of Health and Mental Hygiene.

iii. For Clinical Center patients who are not Maryland residents, the Clinical Center reports HIV-positive results and/or AIDS to the patient's primary care physician.

If you have any questions regarding the HIV testing or the information provided above, you are encouraged to discuss them with your physician or you may call a Clinical Center HIV counselor at 301-496-2381.

GENETIC TESTING

Some of the blood drawn from you as part of this study will be used for genetic tests. Some genetic tests can help researchers study how health or illness is passed on to you by your parents or from you to your children. In vaccine research some genetic tests are done to see if different types of immune response to a vaccine seem to be related to genetic differences in people. Some of the genetic tests will be done in a research lab from your stored samples. Genetic tests done in a research lab will not be in your medical record. Tests that are done in a research lab will not have your name on the sample given to the research lab. In the future, genetic research tests to help understand how vaccines work may be done on your DNA using stored samples.

Some genetic tests are done in a regular medical laboratory. HLA type is a genetic test ordered through the NIH Clinical Center medical laboratory. HLA type results will be in your medical record at the NIH Clinical Center.

HLA Testing

Some of the blood drawn from you as part of this study will be used for a test of HLA type.
HLA type is a genetic test of markers of the immune system. HLA type is usually used to match bone marrow or organ transplants. For research, HLA testing is sometimes used to try to identify factors associated with response to a vaccine, progression of a disease or related conditions. Determining HLA type is necessary to be able to perform certain research studies.
Some HLA types have been associated with an increased risk of certain diseases like arthritis and other rheumatologic problems. Simply having those HLA types, however, doesn't mean you will develop these diseases. Genetic testing can also be used to determine if people are directly related. These tests sometimes show that people were adopted or that their biological parent is

someone other than their legal parent. If these facts were not known previously they could be troubling. Additional genetics counseling and advice is available from the National Institutes of Health to help you understand the nature and implications of genetic findings about you and your family.

Our policy is to not give such information to you unless we believe it has direct medical or reproductive implications for you or your family. By agreeing to participate in this study, you do not waive any rights that you may have regarding access to and disclosure of your records. For further information on those rights, you can contact the principal investigator of this study.

Any genetic information collected or discovered about you or your family will be confidential. Results of HLA testing will become a part of your medical record at NIH. Medical records containing this information are maintained in a secure manner. Genetic information about you will not be revealed to others, including your relatives, without your permission. We will not release any information about you or your family to any insurance company or employer unless you sign a document allowing release of information. Instances are known in which genetic information has been obtained or requested when a person applies for health insurance or a job.

STORED SAMPLES

During your participation on this study blood samples will be collected from you, as already explained. We will store these samples for future research to learn more about HIV and HIV vaccines, the immune system, and/or other medical conditions.
The results from the research done with your stored samples will not be given to your health care provider and will not be put in your medical record. This is because the test results, unlike routine medical testing, will be experimental or preliminary. The relevance of these tests to your care is unknown. At your request however, the results of any research tests will be discussed with you or your physician.

Labeling of Stored Samples

Your stored samples will be labeled by a code (such as a number) that only the study team can link to you. Any identifying information about you will be kept confidential to the extent permitted by law.

Risks from Stored Samples

The greatest risk is the unplanned release of information from your medical records. The chance that this information will be given to an unauthorized person without your permission is very small. Possible problems with the unplanned release of information include discrimination when applying for insurance and employment. Similar problems may occur if you disclose information yourself or agree to have your medical records released.

Future studies

In the future, other investigators (at NIH or outside of NIH) may wish to study your stored samples. When the study team shares your materials, they may share it with no identifying information or with a code. Some information about you, such as your gender, age, health history, or ethnicity may also be shared with other investigators. Any future research studies

using your samples will be reviewed by the investigator's Institutional Review Board (IRB), a special committee that oversees medical research studies to protect the rights and welfare of human subject volunteers.

Your stored materials will be used only for research and will not be sold. The research done with your materials may be used to develop new products in the future but you will not receive payment for such products.

POSSIBLE STUDY RISKS

Injection Risks

It is possible that you may have some side effects from the injections. You will receive your DNA vaccinations in two ways, depending on how you are randomly assigned. You may get injections in the standard way, with a needle and syringe. You may get the injection with a needle-less system called a Biojector. A needleless system uses the pressure of carbon dioxide instead of a needle to inject the vaccine through your skin and into the muscle. Different kinds of needleless systems have been used since 1947 to deliver vaccines and other types of drugs. You will receive your rAd vaccine injection in the standard way, with a needle and syringe. With either type of injection you may have mild discomfort. There may be stinging, arm discomfort, pain, soreness, redness and swelling. These symptoms may last for several days. There is a risk of fainting and a very small chance of infection at the injection site. You will be asked to record and report any side effects. You may need to make extra visits to the clinic for evaluation of side effects. You may use over-the-counter (nonprescription) pain medications, if needed.

General Vaccine Risks

The possible risks for vaccines in general include fever, chills, rash, aches and pains, nausea, headache, dizziness, and fatigue. We know these side effects do occur with other vaccines. The side effects don't usually last long. As with all vaccines or drugs, you could have an immediate allergic reaction, including a rash, hives, or even difficulty breathing. Allergic reactions can be life threatening; therefore, the clinic staff will watch you for at least 30 minutes after each immunization and provide any needed treatment. There may be other side effects, even serious ones that we don't know about yet. Therefore, it is important that you report any side effects to the clinic staff as soon as they occur.

DNA Vaccine Risks

The same DNA vaccine that is in this study was given to 15 people before in the first study in people. The first study started in August 2004 and is also a Vaccine Research Center study at the NIH. All of the risks and side effects are not known. One person developed hives (urticaria) around the time of the second vaccine injection. Hives are a kind of allergic reaction. The hives may have been related to vaccination or to other allergies. One person was found to have a moderately low blood sugar 14 days after the 3rd vaccination, but did not have any symptoms. A different person complained of moderate dizziness for a day on the 13th day after the 2nd

vaccination. This person did not have dizziness after the 3rd vaccination. It is not known if these problems were related to vaccination.

An unexpected finding in the first study was that following 4 of the 44 injections a small red bump and then a scab formed at the injection site. The scab was less than ½ inch across. It was not deep and it was not infected. The scab came off after a few days. The skin healed without needing any treatment.

- One person had a scab after the third vaccination, but not after the first and second.
- One person had a scab after the second vaccination, but not after the first and third.
- One person had a scab after the first and the third vaccination, but not after the second.

The VRC had another study with a similar DNA vaccine. It started in November 2002. That study included 40 people who got vaccine injections and 10 who got placebo (sterile salt water) injections. Laboratory tests that were sometimes temporarily out of the normal range included: blood glucose (sugar), liver tests (bilirubin or liver enzymes), a red blood cell test (hemoglobin) and protein in the urine. It is not known if these changes in laboratory tests were related to vaccine, due to other causes (such as patterns of eating, drinking or an illness) or are due to day-to-day variation in laboratory test results. Changes in lab tests were also seen in people who got placebo injections. None of these temporary changes needed treatment. The following is a list of other conditions possibly related to study vaccinations:

- One person was found to have a very low white blood cell count at 27 days after the third vaccination. The person remained feeling well and the white blood cell count returned to normal without treatment in less than a week.
- One person developed a skin rash after the second vaccine injection, which cleared up without treatment. The cause was unknown, but the third vaccine injection was not given.
- One person developed hives (urticaria) after the third vaccine injection. The hives may have been related to vaccination, related to an infection the subject had, or related to other medicines the subject was taking. The hives were treated and cleared up.

Two other studies with the similar DNA vaccine are still ongoing. One started in the United States in December 2003 and one started in Uganda in January 2005. No new risks of vaccine have been identified.

Possible risks related to DNA vaccines include: muscle damage; antibodies to DNA leading to illness; and insertion of the vaccine DNA into the body's DNA (leading to cancer) or into the DNA of a bacteria or virus in your body. None of these possible risks of DNA vaccines have been seen in laboratory tests or in animals or humans so far, but you need to be aware of these possible risks. Study participants will not be monitored for DNA insertion but will be monitored for clinical signs and symptoms of these effects. During the study some blood will be stored during the study in case additional safety tests are needed.

Adenoviral Vector (rAd) Vaccine Risks

The rAd vaccine has been given to people who have already received an experimental DNA vaccine in other studies. The first rAd booster study started in November 2004 at sites around

the United States and the second one started at the VRC Clinic, NIH in January 2005. This is one of two other rAd booster studies at the VRC Clinic, NIH. The first study of the rAd vaccine alone in people started at the VRC Clinic in July 2004.

After a rAd vaccination, some people have a flu-like condition with fever, headache, muscle aches, tired feeling and chills. It starts about 12-16 hours after vaccination and lasts a few hours. A few people have had nausea. Some people have injection site pain or discomfort in the first few days after a vaccination. The flu-like symptoms and injection site pain or discomfort may be treated with an over-the-counter medicine for pain and fever.

The following is a list of other conditions possibly related to study vaccinations:

- One person had a white blood cell count that was moderately below normal shortly after vaccination. This returned to normal without any symptoms of illness.
- One person had diarrhea for a day shortly after vaccination.
- One person had a mild change in a blood test for liver function that was done 25 days after vaccination. When this mild change did not return to normal, more evaluation was done and the person was found to have a "fatty liver." The condition may have existed before enrollment in the study. In this person, other factors such as increased alcohol use and recent weight gain may be causes of the fatty liver.
- One person with a history of a single seizure about three years before enrolling in the study had a seizure 64 days after the study vaccination. The seizure was considered unrelated to the study vaccination because it happened more than 2 months after vaccination in a person with a history of having a seizure about 3 years before enrolling in the study.
- Other people have had mild temporary changes in blood or urine tests.

It is not known if these problems were due to the vaccine or due to other causes or due to a combination of the vaccine with other causes.

Animals have tolerated a vaccination schedule with a DNA vaccine given first followed by the rAd vaccine. A schedule of the rAd vaccine alone was tested in rabbits. A schedule of a similar DNA vaccine followed by the rAd vaccine was also tested in rabbits. In both schedules some rabbits had a fever the day after rAd vaccine injection. Some rabbits ate less food than usual for 1-2 days after rAd vaccine injection. In some rabbits there was inflammation in the tissue near the injection site.

<u>Other Adenoviral Vectors</u>: Other adenoviral vector vaccines and products have been given to humans in other studies that tested treatment of cancer and inherited conditions. In one study a volunteer died from a reaction to an adenovirus vector after he was given a large dose directly into the main liver blood vessel for the purpose of treating an inherited condition. In the study in which you will be participating much lower doses are used and the injection is into your arm muscle.

During the study, regular blood tests and check-ups will be performed to check for possible side effects. Some blood will be stored during the study in case additional safety tests are needed.

Adenovirus antibodies: You may develop antibodies to adenovirus type 5 from the rAd vaccine. It is possible you would not be able to receive (or have a reduced response to) future products that used an adenoviral vector. Currently there are no products approved by the FDA that use an adenoviral vector.

Risks from Blood Drawing
Blood will be drawn from a vein in your arm using a needle. Blood drawing may cause pain and bruising and, rarely, infection at the place where the blood is taken. Sometimes drawing blood causes people to feel lightheaded or even faint.

HIV Vaccine Risks
You will be counseled about HIV exposure during the study. If you have questions, please ask the clinical staff. Getting the experimental vaccines in this study may mean that you cannot be in other experimental HIV vaccine studies later. It is also possible that receiving the experimental HIV vaccine may alter your response to future HIV vaccines and may make them either more or less effective. If you are exposed to HIV through sex or drug use after receiving the study injection, your risk of becoming HIV infected is unknown. Please do not do anything that might expose you to HIV.

You should be aware that some people who received experimental HIV vaccines in the past became infected with HIV through sex or drug use. We know that HIV infection and AIDS can develop even in a person who has received a test vaccine if he/she is exposed to HIV. If you are exposed to HIV through sex or drug use after receiving the study vaccines, your risk of becoming infected with HIV and developing AIDS is unknown. Please inform the VRC Clinic any time you think that you may have been exposed to HIV. If you do get infected, we do not know what effect the study vaccines may have on the disease. The time that it takes for you to become sick from HIV/AIDS may be the same, longer or shorter than usual. You will be educated and counseled about HIV exposure often during the study. If you have questions, please ask the clinic staff.

Risk of Developing a "False" Positive HIV Test
At the time you enroll in the study you must have a negative HIV antibody test. An HIV antibody test (called an ELISA or Western Blot) is the usual way to test for HIV infection. After the study vaccinations, it is likely that you will test positive for HIV antibody test from the study vaccines. However, it will be possible, by using tests for the presence of HIV virus (called PCR or viral load testing), to show when a positive result on the HIV antibody test is NOT because of an HIV infection. A positive antibody test in a person who is not HIV infected is called a "false positive" test. If you do have a false positive HIV antibody test caused by the experimental vaccine, it is unknown how long the test will be positive. Antibodies resulting from a vaccine do not always continue to be present long term. If you have a false positive antibody test at the end of the study you will be offered the chance to be retested once per year for five years so that you can find out if it changes back to negative. You may be subjected to the social risks of having your HIV test appear positive. For this reason you are advised to have all HIV testing done at

the VRC Clinic while you are participating in the study. Counseling about HIV tests, including social problems related to false positive results, is offered at all clinic visits. You may also call the clinic at other times if you have questions or concerns.

Any time you have a positive HIV antibody test in the future you must also have an HIV viral load test. Otherwise you will not know if the positive HIV antibody test is from the study vaccines or from HIV infection.

You will not be able to donate blood while you are participating in the study and for at least one year after the last study injection. You may not be able to donate blood ever again if you have a false positive test when you try to donate blood. Please be sure you have a negative HIV antibody test before trying to donate blood.

If you have a false positive antibody response on HIV tests, you may also have difficulties with:

- Health insurance
- Life Insurance
- Medical or dental care
- Travel to other countries or immigration
- Employment
- Education
- Housing
- Military services or other government agencies
- Personal relationships

If you have problems like these, the staff at the clinic will try to help you work through them. If your blood tests look HIV positive because of study vaccinations you will be offered a letter that shows you joined this study and that describes the antibody response caused by the vaccine. Even so, this letter or other help offered by the VRC Clinic may not solve a social problem caused by a false positive HIV antibody test.

It is also possible that others may learn that you are taking part in this study and assume that you are at risk of HIV infection because of sexual behavior or drug use. This may result in some people treating you differently.

Risks from Pregnancy

We do not know the possible effects of the study vaccines on the fetus or nursing infant. Therefore, women who are able to become pregnant must have a negative pregnancy test before each immunization. Women must also agree to practice adequate birth control beginning at least 21 days prior to receiving the first injection until the last study visit at Week 42, or not be able to

have children. Adequate methods of birth control include: condoms, male or female, with or without a spermicide; diaphragm or cervical cap with spermicide; intrauterine device; contraceptive pills, Norplant, or Depo-Provera; male partner who has previously undergone a vasectomy for which there is documentation. If you are pregnant, breast-feeding or want to become pregnant during the next 42 weeks, you cannot participate. You must notify the clinic staff immediately upon learning that you have become pregnant during this study. If you become pregnant, you will receive no further injections. However, you will be asked to continue with study follow-up visits through the full 42 weeks of study visits, as well as to report the outcome of the pregnancy.

Other Risks

Since the study vaccines have not been administered to many humans, their safety and toxicity is uncertain. It is possible that new and unexpected side effects may develop in humans that were not observed in animal testing or previous clinical trials. You will be made aware of significant health effects of the vaccines and serious side effects that occur in other volunteers. You will be updated during the trial as needed.

POSSIBLE BENEFITS

This study may be of no direct benefit to you because no one knows if vaccines against HIV work. However, you and others may benefit in the future from the information that will be learned from the study.

COSTS TO YOU FOR YOUR PARTICIPATION

You do not have to pay for the vaccines, research clinic visits, examinations or laboratory tests that are part of this study. All medical costs outside this study will be paid by you or your health insurance carrier (if you have insurance).

PAYMENT TO YOU FOR YOUR PARTICIPATION

You will be compensated $70 for each visit with blood drawing but no injection. You will be compensated $100 for each visit that includes an injection and blood drawing. If you have a skin biopsy there is additional compensation of $70 per biopsy above the usual compensation for the type of visit. You will be compensated $30 for the visits to photograph and look at the injection site, including removal of skin biopsy stitches, if needed. The approximate total compensation for the 42 weeks of study is between $1050 and $1140 without any skin biopsies. Actual compensation is based on the number of study visits you attend, number of study injections you receive and if you have any skin biopsies. You will be paid throughout the study after each reimbursable visit. If you have blood drawn at Week 94 you will be compensated $70 for this extra long-term visit.

REASONS FOR REMOVING YOU FROM THE STUDY WITHOUT YOUR CONSENT

You may be asked to leave the study for several different reasons, including:

- You don't keep appointments or follow study procedures
- The study sponsor or study doctor decide to stop or cancel the study

- The regulatory board known as the “Institutional Review Board” or the FDA decide that the study should be stopped

If you agree to take part in this study, it is important for you to keep all your appointments. However, if you don't want to stay in the study, you can leave at any time. You will not lose any benefits that you would have had if you had not joined the study. If the study vaccinations are not completed for any reason you will be asked to continue with follow-up visits.

ALTERNATIVES

You may choose to not participate in any HIV vaccine study. You may be eligible for other studies, including those testing other experimental HIV vaccines. Your study doctor can discuss the risk and benefits of alternative studies.

COMMUNITY RESOURCES

You may also be interested in contacting local volunteer panels of individuals from the general public that were organized to assist and advise AIDS vaccine trials in the metropolitan Washington DC area, the Capital Area Vaccine Effort (CAVE) and the Community Advisory Board (CAB). Information is available at the Internet site http://www.aidsvaccine.org.

OTHER PERTINENT INFORMATION

1. Confidentiality. When results of an NIH research study are reported in medical journals or at scientific meetings, the people who take part are not named and identified. In most cases, the NIH will not release any information about your research involvement without your written permission. However, if you sign a release of information form, for example, for an insurance company, the NIH will give the insurance company information from your medical record. This information might affect (either favorably or unfavorably) the willingness of the insurance company to sell you insurance.

The Federal Privacy Act protects the confidentiality of your NIH medical records. However, you should know that the Act allows release of some information from your medical record without your permission, for example, if it is required by the Food and Drug Administration (FDA), members of Congress, law enforcement officials, or other authorized people.

2. Policy Regarding Research-Related Injuries. The Clinical Center will provide short-term medical care for any injury resulting from your participation in research here. In general, no long-term medical care or financial compensation for research-related injuries will be provided by the National Institutes of Health, the Clinical Center, or the Federal Government. However, you have the right to pursue legal remedy if you believe that your injury justifies such action.

3. Payments. The amount of payment to research volunteers is guided by the National Institutes of Health policies.

4. Problems or Questions. If you have any problems or questions about this study or about any research-related injury, contact the Principal Investigator, Andrew Catanzaro, M.D. at 301-402-8604, Subinvestigator, Barney S. Graham, M.D., Ph.D. at 301-594-8468 or Study Coordinator Ingelise Gordon, RN 301-451-8715 or 1-800-NIH-BEEP ext 14881.

If you have any questions about your rights as a research subject, you may call the Clinical Center Patient Representative at 301-496-2626.

5. Consent Document. Please keep a copy of this document in case you want to read it again.

COMPLETE APPROPRIATE ITEM(S) BELOW:

A. Adult Study Participant's Consent	B. Parent's Permission for Minor Participant.
I have read the explanation about this study and have been given the opportunity to discuss it and to ask questions. I hereby consent to take part in this study.<br><br>Time<br><br>Signature of Adult Participant/Legal Representative Date	I have read the explanation about this study and have been given the opportunity to discuss it and to ask questions. I hereby give permission for my child to take part in this study.<br>(Attach NIH 2514-2, Minor's Assent, if applicable.)<br><br>Signature of Parent(s)/Guardian Date

C. Child's Verbal Assent (If Applicable)
The information in the above consent was described to my child and my child agrees to participate in the study.

Signature of Parent(s)/Guardian Date

THIS CONSENT DOCUMENT HAS BEEN APPROVED FOR USE FROM XXXXXX THROUGH XXXXXX.

Time: Time:

Signature of Investigator/Person Obtaining Consent Date Signature of Witness Date

APPENDIX II
CONTACT INFORMATION

Principal Investigator:
Andrew Catanzaro, M.D.
Vaccine Research Center, NIAID, NIH
40 Convent Drive, MSC 3017
Bethesda, MD 20892-3017
301-402-8604, 1-800-NIH-BEEP ext 14881

Subinvestigators:
Barney S. Graham, M.D., Ph.D. 301-594-8468
Joseph Casazza, M.D., Ph.D. 301-594-8627
Julie Martin, D.O. 301-594-8559

Study Coordinators
Ingelise Gordon, RN 301-451-8715
VRC 008 Study Coordinator

Lasonji Holman, RN, NP
Sarah A. Hubka, RN, MSN, NP
Brenda Larkin, RN, BSN, CCRC
Laura Novik, RN, MA, CCRC
Steve Rucker, RN

DAIDS Medical Officer:
Chuen-Yen Lau, M.D. 301-451-2779
6700 B Rockledge Dr., Bethesda, MD 20892

Protocol Statistician:
Martha Nason, Ph.D. 301-451-5134
Biostatistical Research Branch, NIAID, NIH

Data Coordinating Center:
Vaccine Research Center, NIAID, NIH and
EMMES Corporation, Rockville, MD

Study Site:
National Institutes of Health Clinical Center
12 West Outpatient Clinic
Bethesda, MD 20892

Site And Data Monitoring:
PPD Development
Wilmington, NC

Scientific and Laboratory Collaborators:
Gary Nabel, M.D., Ph.D.
Vaccine Research Center, NIAID, NIH
40 Convent Drive, MSC 3017
Bethesda, MD 20892

Laboratory of Immunology
VRC/NIAID/NIH:

Robert Bailer, Ph.D., 301-594-8481
Daniel Douek, M.D, Ph.D., 301-594-8484
Richard Koup, M.D., 301-594-8585
John Mascola, M.D., 301-594-8490
Mario Roederer, Ph.D., 301-594-8491

Vaccine Manufacturers:
Vical, Incorporated
373 Towne Centre Dr., Suite 100
San Diego, CA 92121-3027

GenVec, Incorporated
65 Watkins Mill Road
Gaithersburg, MD 20878

Pharmacy Affairs Branch:
Judith Starling, R.Ph., Hope DeCederfelt, R.Ph.
Pharmaceutical Development Section
Clinical Center, Building 10/1N257
Bethesda, MD 20892
301-496-4363

VRC Production and Regulatory Affairs:
Phillip Gomez III, Ph.D., 301-594-8485
Judy Stein, MPH, MBA, 734-763-7753
Charla Andrews, Sc.M., 301-594-8488

VRC Protocol Section:
Mary E. Enama, M.A., PA-C, 301-594-8501
Richard Jones, 301-451-8543

DAIDS, Regulatory Compliance Center:
SAE Phone: 1-800-537-9979 or 301-897-1709
SAE Fax: 1-800-275-7619 or 301-897-1710
SAE e-mail: RCCSafetyOffice@tech-res.com

APPENDIX III
SCHEDULE OF EVALUATIONS

		**VRC 000 Screen**	**VRC 008 DNA Prime Vaccinations and Follow-up Visits**												
Visit		*01	02	02B	02C	02D	03	03B	03C	03D	04	04B	04C	04D	05
Week of Study			Wk 0	W 1	W1	W 2	W 4	W 5	W5	W 6	W 8	W 9	W9	W 10	W 12
[1]Day of Study			D 0	D 3	D 7	D 14	D 28	D 31	D 35	D 42	D 56	D59	D 63	D 70	D 84
**Clinical**															
VRC000 Screening consent		X													
VRC 008 AoU & Consent			X												
[2] Physical exam		*X	[X]	[X]	[X]	[X]	[X]	[X]	[X]	[X]	[X]	[X]	[X]	[X]	[X]
Complete or interim Med Hx;Vital signs,weight		*X	X	X	X	X	X	X	X	X	X	X	X	X	X
Lymph node assessment		X	X	X	X	X	X	X	X	X	X	X	X	X	X
Study Injections (photo of arm prior to injection)			X				X				X				
[3] "B" visit: inject site photo, [optional skin biopsy] "C" visit: telephone only if no lesion				X	X			X	X			X	X		
5-day Diary Card			start		return		start		return		start		return		
[4] Counsel HIV; pregnancy		X	X	X	X	X	X	X	X	X	X	X	X	X	X
Urinalysis		*X	X			X	X			X	X			X	
CBC, differential, platelets	Lav.	*3	3			3	3			3	3			3	3
PT, PTT	Blue	*5													
[5] Preg test: urine (or serum)		X	X				X				X				
Screening: Chem 20; fasting glucose, LDL, chol. Other visits: creatinine, ALT	SST	*4	4			4	4			4	4			4	4
HBsAg, Anti-HCV, HCV PCR	SST	*8													
HLA class I, II antigens	ACD					20									
ELISA/Western Blot	SST	*4	4												4
HIV PCR	Lav	3	3												3
T cell FACS	Lav	3	3												3
Anti-dsDNA, RPR, Immunoglobulins	SST	4													
**Research**															
Adenovirus Serology	SST	4	X												
HIV-specific antibody	SST		16								16				16
ICS and ELISPOT; PBMC & Plasma for Storage	EDTA	60	80							60	80			60	80
Serum Storage	SST	24	16				8			24	16			24	16
**Daily Volume (mL)**		**122**	**129**			**27**	**15**			**91**	**119**			**91**	**129**
**Cumulative Volume (mL)**		**122**	**251**			**278**	**293**			**384**	**503**			**594**	**723**

* VRC 000 Screening evaluations may be completed over several screening visits. Each evaluation must be in the specified window for the test. Pregnancy test on Day 0 must be used for eligibility. If clinical assessment on Day 0 suggests significant changes since the screening visit, then physical exam, hematology, ALT, creatinine and urinalysis done on Day 0 must be used for eligibility.

[1] Day 0=day of enrollment and first injection; Day 0 evaluations prior to first injection are the baseline for assessing adverse events subsequently. There must be least 21 days between DNA injections. Schedule the "D" visit for 14(±3) days after each DNA injection and Visit 05 for Week 12±7 days. Week 24 to end of study is shown on the next page of this table.

Note: In the study database, "A" visits are the evaluations (vital signs and injection site assessment) completed between 30 to 45 minutes after a study injection.

[2] Screening visit includes a physical exam; during other visits a physical exam is done if indicated by interim history or laboratory test results (shown as [X] in the table).

[3] Clinic visit is required for all "B" visits at 3(±1) days after DNA vaccinations. "C" visit is 7(±1) days after DNA vaccination and may be telephone contact if no evidence of skin lesion formation. It must be clinic visit if evidence of skin lesion formation. Continue to photo injection site until skin is healed. Skin biopsy is optional, but no more than two skin lesion biopsies per subject for research purposes. Preferred timing of biopsy is at earliest visit with evidence of a skin lesion. Additional skin biopsies may be done, with subject consent, if needed for clinical care purposes.

[4] Counseling at Screening and on Day 0 and offered on each subsequent visit

[5] Negative pregnancy test results must be confirmed for women of reproductive potential prior to administering study injections.

		**Adenoviral Vector Vaccine (rAd) Boost and Follow-up Visits**						**Long-term Follow-up**
Visit		06	06B	06C	07	08	09	10
Week of Study		W 24	W24	W26	W28	W30	W42	W94
[1]Day of Study		D168	D170	D182	D196	D210	D294	D658
**Clinical**	**Tube Type**							
[2] Physical exam		[X]		[X]	[X]	[X]	[X]	
Complete/interim Med Hx; Vital signs and Weight		X		X	X	X	X	
Lymph node assessment		X		X	X			
Study Injections		X						
[3] Phone evaluation (clinic visit if needed)			X					
5-day Diary Card		start		return				
Social Impact Assessment							X	
[4] Counsel HIV; pregnancy		X		X	X	X	X	
Urinalysis		X		X				
CBC, differential, platelets	Lav.	3		3	3	3	3	
[5] Pregnancy test: urine (or serum)		X					X	
Creatinine, ALT	SST	4		4	4	4	4	
[6] Long-term follow-up by clinic visit, phone, e-mail or mail								X
ELISA/Western Blot	SST	4				4	4	[4]
HIV PCR	Lav	3				3	3	[3]
T cell FACS	Lav	3				3	3	
**Research**								
Adenovirus Serology	SST	X			X		X	
HIV-specific antibody	SST	16			16		16	[16]
ICS and ELISPOT; also PBMC & Plasma for Storage	EDTA	80			80	80	80	[80]
Serum Storage	SST	16			16	16	16	[16]
**Daily Volume (mL)**		**129**		**7**	**119**	**113**	**129**	**[119]**
**Cumulative Volume (mL)**		**852**		**859**	**978**	**1091**	**1220**	

[1] Day 0 through Week 12 visits are shown on the previous page. Schedule the rAd vaccination for Week 24 (day 168 with a -7 or +14 day window). The telephone evaluation (visit 06B) is 1 or 2 days after the rAd injection and visit 06C is 14±3 days after the rAd injection; other follow-up visits are Visit 07 (Week 28±7 days), visit 08 (Week 30±7 days) and visit 09 (Week 42±14 days). If the rAd injection occurs on day 176 through 182 then visit 07 and 08 may be adjusted further to keep the interval after the rAd injection close to 4 weeks and 6 weeks post injection, respectively.
Note: In the study database, "A" visits are the evaluations (vital signs and injection site assessment) completed between 30 to 45 minutes after a study injection.
[2] Physical exam is done if indicated by interim history or laboratory test results (shown as [X] in the table).
[3] A clinic visit is required if there is rash, urticaria (hives), fever of 38.7˚C (Grade 2) or higher, or significant impairment in the activities of daily living (ADL)
[4] Counseling offered on each visit.
[5] Negative pregnancy test results must be confirmed for women of reproductive potential prior to administering study injection.
[6] Long-term follow-up Visit 10 is Week 94 (±28 days). The brackets [ ] indicate that blood samples for HIV testing and research immunology are optional. The protocol also allows subjects with vaccine-induced antibody, who choose to do so, to return for HIV testing annually for five years after visit 09.

APPENDIX IV
TABLE FOR GRADING SEVERITY OF ADVERSE EVENTS

The Division of AIDS Table for Grading the Severity of Adult and Pediatric Adverse Events, Version 1.0, Dec 2004

The table for Grading Severity of Adverse Events in this protocol is found on the Division of AIDS Regulatory Compliance Center (RCC) website:

http://rcc.tech-res-intl.com/eae.htm

A complete copy of this 20 page table will be provided to the IRB for reference with initial review of the protocol. The table cannot be changed except by the IND Sponsor, DAIDS.

The full text of the table will also be included in the Protocol Manual for reference by the study clinicians who are assessing adverse events.

APPENDIX V
SKIN BIOPSY INFORMED CONSENT FORM

INSTITUTE: Vaccine Research Center, National Institute of Allergy and Infectious Diseases

STUDY NUMBER: 05-I-0148 PRINCIPAL INVESTIGATOR: Andrew Catanzaro, M.D.

STUDY TITLE: VRC 008: A Phase I Clinical Trial of a Prime-Boost HIV-1Vaccination Schedule: Multiclade DNA Vaccine, VRC-HIVDNA016-00-VP, Followed by Multiclade Adenoviral Vector Vaccine, VRC-HIVADV014-00-VP, in Uninfected Adult Volunteers

Latest IRB Review:
Latest Amendment Approved:

Skin Biopsy Consent

INTRODUCTION

We invite you to take part in a research study at the National Institutes of Health (NIH).

First, we want you to know that:

> Taking part in NIH research is entirely voluntary.
>
> You may choose not to take part, or you may withdraw from the study at any time. In either case, you will not lose any benefits to which you are otherwise entitled. However, to receive care at the NIH, you must be taking part in a study or be under evaluation for study participation.
>
> You may receive no benefit from taking part. The research may give us knowledge that may help people in the future.

Second, some people have personal, religious or ethical beliefs that may limit the kinds of medical or research treatments they would want to receive (such as blood transfusions). If you have such beliefs, please discuss them with your NIH doctors or research team before you agree to the study.

Now we will describe this research study. Before you decide to take part, please take as much time as you need to ask any questions and discuss this study with anyone at NIH, or with family, friends or your personal physician or other health professional.

PURPOSE OF THE STUDY

You are participating in a study of two experimental HIV vaccines. These vaccines are known as VRC-HIVDNA016-00-VP and VRC-HIVADV014-00-VP. We call the first one “the DNA vaccine” and the second one the “rAd vaccine.” The DNA vaccine sometimes will cause a red

bump on the arm at the vaccination site that forms a scab after a few days. This consent is to offer you the choice of having a skin biopsy or not having a skin biopsy of your DNA vaccination site. The skin biopsy is for research purposes to better understand how the skin is reacting to the DNA vaccine.

A skin biopsy is a medical procedure. A skin biopsy removes a small piece of skin. The purpose is to look at the skin under a microscope. A separate consent for the skin biopsy is needed for two reasons. First, you may choose to not have a skin biopsy. Second, you should understand the way a skin biopsy is done and the risks of a skin biopsy before deciding whether to have one.

You are eligible to have a skin biopsy because you recently had a vaccination in the VRC 008 study. As many as forty (40) people will receive vaccinations in the VRC 008 study. Some of the 40 people may have two skin biopsies, some may have one skin biopsy and some may not have any skin biopsies. It would be unusual for anyone to have more than two biopsies during this study. However, more than two skin biopsies may be recommended if needed for clinical care purposes. If you have a skin biopsy, it will be done at the National Institutes of Health Clinical Center. You will also be treated there if any side effects of the skin biopsy occur.

You will be told of any new information learned during this study that might cause you to change your mind about having a skin biopsy. Your skin biopsy report will be given to you if you wish to have a copy.

STUDY PROCEDURES

A skin biopsy is done in an outpatient clinic. You will receive a medicine (such as lidocaine with epinephrine) on your upper arm to reduce pain. This is called an anesthetic. The anesthetic may be given by injection or applied to the skin. The anesthetic may sting briefly. If you have ever had an allergic reaction to a numbing medicine, such as novocaine or lidocaine, which are commonly used by dentists or surgeons, do not sign this consent until the details of your allergic reaction have been discussed with a study doctor.

After the skin is numb, a sharp, hollow instrument will be used to remove a small, circular piece of skin. The circle will be about the size of a pencil eraser. This biopsy method is called a "punch biopsy." After the punch biopsy, the skin may be closed with one or two stitches. You must follow the directions given to you for keeping the area clean until it heals. You must return to the clinic for the appointment given to you to have the stitches removed several days later. Careful methods and clean equipment will be used to prevent infection.

Microscope slides will be made in the laboratory from the piece of skin. The slides may have different stains and tests done to understand more about skin reactions to the study vaccine. The slides and photos of the slide may be kept as a record of the skin biopsy. At the NIH Clinical Center the skin biopsy sample and any slides made from it will be labeled with your name when it is part of your medical record or the hospital laboratory records. If photos of the slides are

used in reports of study results they will be identified only with a number or code, but will not be labeled with your name. The results of your skin biopsy and photos from the slides will be recorded in the study records labeled only with your study identification number.

RISKS OF SKIN BIOPSY

Any time the skin is opened there is a chance of infection. Infection after a skin biopsy is rare and care will be taken to try to prevent infection. You may have pain or discomfort during the skin biopsy or while it is healing. You may have a very small amount of bleeding right after the skin biopsy. You may have a small scar. If you have skin that tends to form large scars of the type called "keloids", then you will have risk of a keloid forming at the biopsy area. A history of keloid formation increases the risk for keloid scarring from the skin biopsy. If you have a known history of keloid scarring you are advised to inform the study staff and not have a skin biopsy that is only for research purposes.

Rarely, the anesthetic medicine used to numb the skin may cause an allergic reaction. The anesthetic may also interact with certain other medicines to cause serious side effects. Be sure to review all your medications and drug use with the study staff before agreeing to have a local anesthetic. Certain antidepressant medicines and illicit drugs such as cocaine are among the drugs that can react with anesthetics to cause side effects.

BENEFITS

You may have no benefit from the skin biopsy. If your skin has a medical condition you may learn more about the cause of the problem.

COSTS TO YOU FOR YOUR PARTICIPATION

There will be no charge to you or your health insurance company for the skin biopsy or care of the skin biopsy. However, the costs of any other medical care during the period will be charged to you or your insurance company.

PAYMENT TO YOU FOR YOUR PARTICIPATION

You will be paid $70 above the usual compensation for a visit that includes a skin biopsy. The skin biopsy stitches will be removed about 7-10 days later at one of the visits described in the study consent that include a photograph. The compensation for a photo visit is $30 whether or not a stitch also needs to be removed at the same time.

ALTERNATIVES

You may choose to not have a skin biopsy.

OTHER PERTINENT INFORMATION

1. Confidentiality. When results of an NIH research study are reported in medical journals or at scientific meetings, the people who take part are not named and identified. In most cases, the NIH will not release any information about your research involvement without your written permission. However, if you sign a release of information form, for example, for an insurance company, the NIH will give the insurance company information from your medical record. This information might affect (either favorably or unfavorably) the willingness of the insurance company to sell you insurance.

The Federal Privacy Act protects the confidentiality of your NIH medical records. However, you should know that the Act allows release of some information from your medical record without your permission, for example, if it is required by the Food and Drug Administration (FDA), members of Congress, law enforcement officials, or other authorized people.

2. Policy Regarding Research-Related Injuries. The Clinical Center will provide short-term medical care for any injury resulting from your participation in research here. In general, no long-term medical care or financial compensation for research-related injuries will be provided by the National Institutes of Health, the Clinical Center, or the Federal Government. However, you have the right to pursue legal remedy if you believe that your injury justifies such action.

3. Payments. The amount of payment to research volunteers is guided by the National Institutes of Health policies.

4. Problems or Questions. If you have any problems or questions about this study or about any research-related injury, contact the Principal Investigator, Andrew Catanzaro, M.D. at 301-402-8604, Subinvestigator, Barney S. Graham, M.D., Ph.D. at 301-594-8468 or Study Coordinator Ingelise Gordon, RN 301-451-8715 or 1-800-NIH-BEEP ext 14881.

If you have any questions about your rights as a research subject, you may call the Clinical Center Patient Representative at 301-496-2626.

5. Consent Document. Please keep a copy of this document in case you want to read it again.

COMPLETE APPROPRIATE ITEM(S) BELOW:

A. Adult Study Participant's Consent	B. Parent's Permission for Minor Participant.
I have read the explanation about this study and have been given the opportunity to discuss it and to ask questions. I hereby consent to take part in this study.<br>______ Time	I have read the explanation about this study and have been given the opportunity to discuss it and to ask questions. I hereby give permission for my child to take part in this study.<br>(Attach NIH 2514-2, Minor's Assent, if applicable.)
Signature of Adult Participant/Legal Representative ______ Date	Signature of Parent(s)/Guardian ______ Date

C. Child's Verbal Assent (If Applicable)
The information in the above consent was described to my child and my child agrees to participate in the study.

Signature of Parent(s)/Guardian ______ Date

THIS CONSENT DOCUMENT HAS BEEN APPROVED FOR USE FROM XXXXXX THROUGH XXXXXX.

Time: ______	Time: ______
Signature of Investigator/Person Obtaining Consent ______ Date	Signature of Witness ______ Date
